# Supplementary material for: A phase 1b randomised controlled trial of a glucagon-like peptide-1 and glucagon receptor dual agonist IBI362 (LY3305677) in Chinese patients with type 2 diabetes
Source: Nat Commun. 2022 Jun 24;13:3613. doi: 10.1038/s41467-022-31328-x (PMC9232612; doi:10.1038/s41467-022-31328-x)
Supplement: Supplementary file 1 — Supplementary Information [file 41467_2022_31328_MOESM1_ESM.pdf]

A phase 1b randomised controlled trial of a glucagon-like peptide-1 and glucagon receptor dual agonist IBI362 (LY3305677) in Chinese patients with type 2 diabetes

Supplementary Information

1. Supplementary Figures
2. Supplementary Tables
3. Supplementary Note 1: Clinical Study Protocol
4. Supplementary Note 2: Statistical Analysis Plan

Supplementary Figures

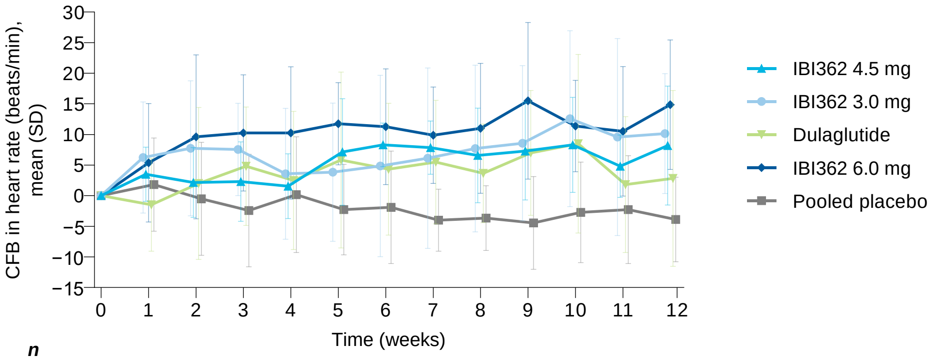

n													
IBI362 3.0 mg	8	8	8	7	7	7	7	7	7	7	7	7	7
IBI362 4.5 mg	8	8	7	7	7	7	7	7	7	7	7	6	5
IBI362 6.0 mg	8	8	8	8	8	8	8	8	8	8	8	8	8
Pooled placebo	12	12	12	12	11	11	11	11	11	11	11	11	10
Dulaglutide	6	6	6	6	6	6	6	6	6	6	6	6	6

Fig. S1: Change from baseline in heart rate over time.

Data are from standard 12-lead electrocardiogram, measured at 1 hour before each dose in the morning from week 0 to week 11, and 168 hours after the last dose (week 12). Data are plotted as means +/- SD. CFB = change from baseline; SD = standard deviation. Source data are provided as a Source Data file.

a b

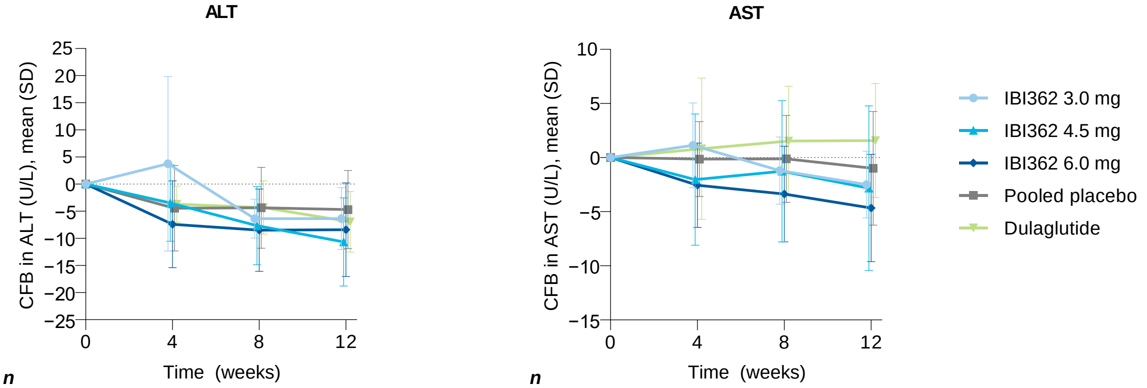

n				
IBI362 3.0 mg	8	7	7	7
IBI362 4.5 mg	8	7	7	5
IBI362 6.0 mg	8	8	8	8
Pooled placebo	12	11	11	10
Dulaglutide	6	6	6	6

n				
IBI362 3.0 mg	8	7	7	7
IBI362 4.5 mg	8	7	7	5
IBI362 6.0 mg	8	8	8	8
Pooled placebo	12	11	11	10
Dulaglutide	6	6	6	6

Fig. S2: Change from baseline in ALT and AST levels over time.

a. CFB in ALT levels over time. **b**. CFB in AST levels over time. Data are plotted as means +/- SD. ALT = alanine aminotransferase; AST = aspartate aminotransferase; CFB = change from baseline; SD = standard deviation. Source data are provided as a Source Data file.

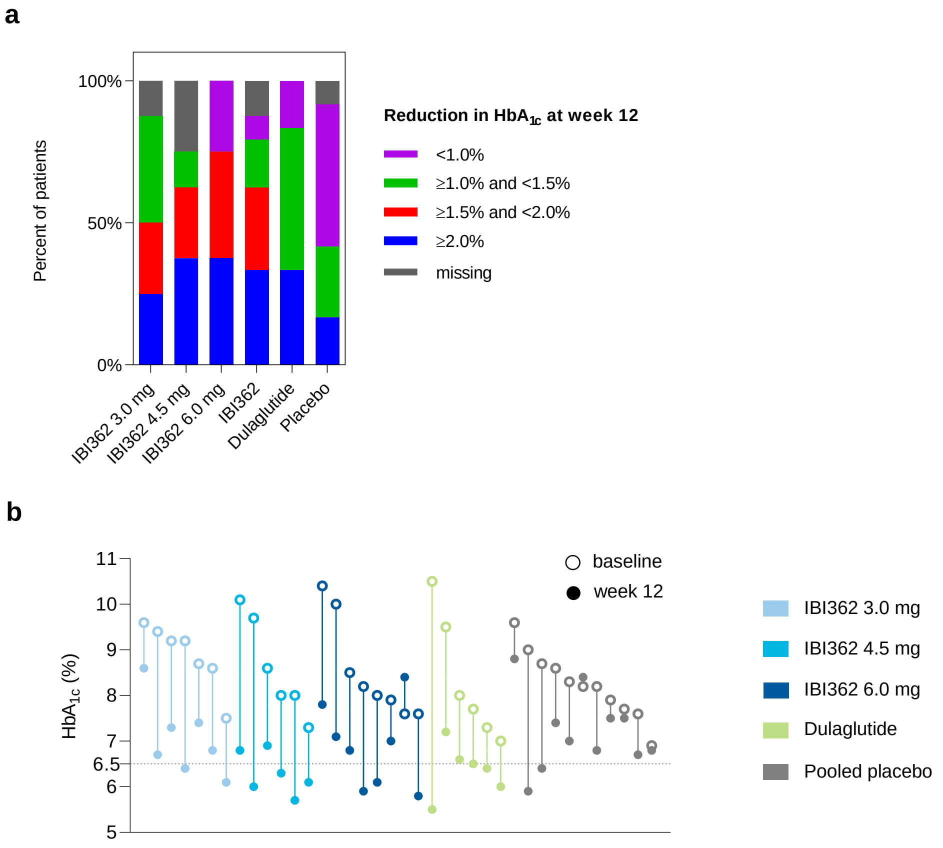

Fig. S3: Percent of patients achieving indicated HbA_{1c} reduction at week 12 and HbA_{1c} levels at baseline and week 12 of each patient.

a. Percent of patients achieving indicated HbA_{1c} reduction at week 12. **b.** HbA_{1c} levels at baseline and week 12 of each patient. CFB = change from baseline; HbA_{1c} = glycated haemoglobin A_{1c}. Source data are provided as a Source Data file.

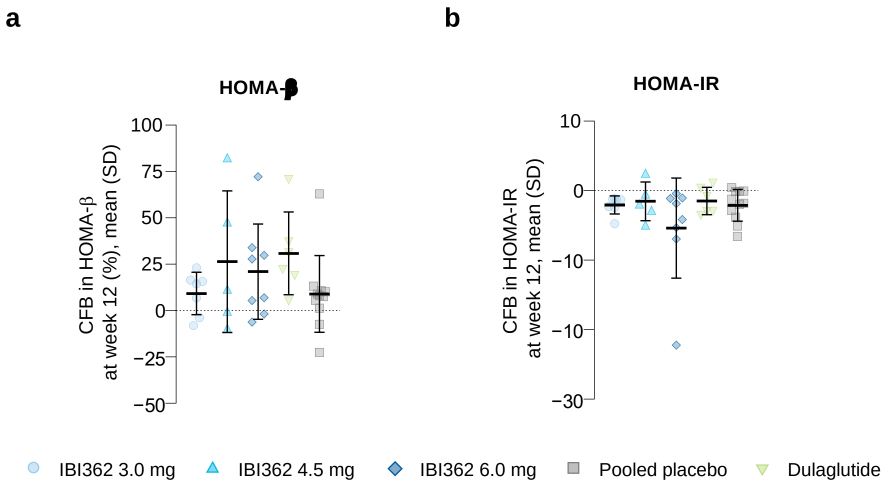

Fig. S4: Change from baseline in HOMA-β and HOMA-IR at week 12.

a. CFB in HOMA-β at week 12**. b.** CFB in HOMA-IR at week 12**.** Data are plotted as means +/- SD. IBI362 3.0 mg *n* = 7; IBI362 4.5 mg *n* = 5; IBI362 6.0 mg *n* = 8; Pooled placebo *n* = 11; Dulaglutide *n* = 6. CFB = change from baseline; SD = standard deviation. Source data are provided as a Source Data file.

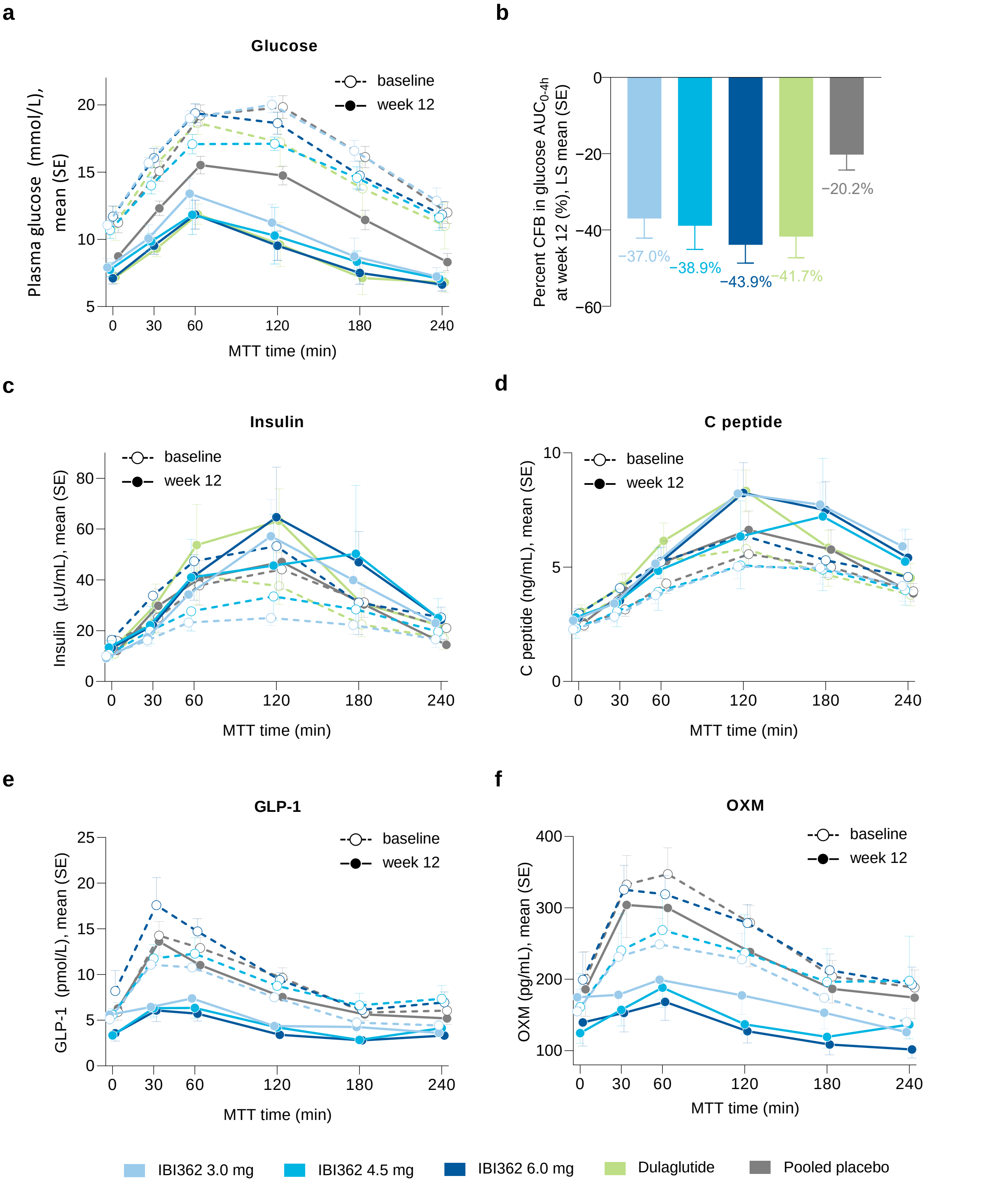

Fig. S5: Excursion of glucose, insulin, C peptide, GLP-1 and OXM during MTT at baseline and at week 12.

a. Plasma glucose concentrations excursion during MTT at baseline and at week 12. **b**. Percent change from baseline to week 12 in post-MTT glucose $AUC_{0\text{-}4\ h}$. **c-d**. Excursion of insulin (c) and C peptide (d) during MTT at baseline and at week 12. **e-f**. Excursion of GLP-1 (e) and OXM (f) during MTT at baseline and at week 12. Data in **a, c, d ,e, f** are plotted as means +/- SEM. Baseline: IBI362 3.0 mg *n* = 8; IBI362 4.5 mg *n* = 8; IBI362 6.0 mg *n* = 8; Pooled placebo *n* = 12; Dulaglutide *n* = 6. Week 12: IBI362 3.0 mg *n* = 7; IBI362 4.5 mg *n* = 5; IBI362 6.0 mg *n* = 8; Pooled placebo *n* = 11; Dulaglutide *n* = 6. Data in **b** are plotted as LS means +/- SEM from an MMRM model, with LS means at week 12 shown alongside. IBI362 3.0 mg *n* = 8; IBI362 4.5 mg *n* = 8; IBI362 6.0 mg *n* = 8; Pooled placebo *n* = 12; Dulaglutide *n* = 6. CFB = change from baseline; GLP-1 = glucagon like peptide-1; MMRM = mixed-effect model for repeated measures; MTT = mixed meal test; OGIS = oral glucose insulin sensitivity; OXM = oxyntomodulin; SE = standard error of the mean. Source data are provided as a Source Data file.

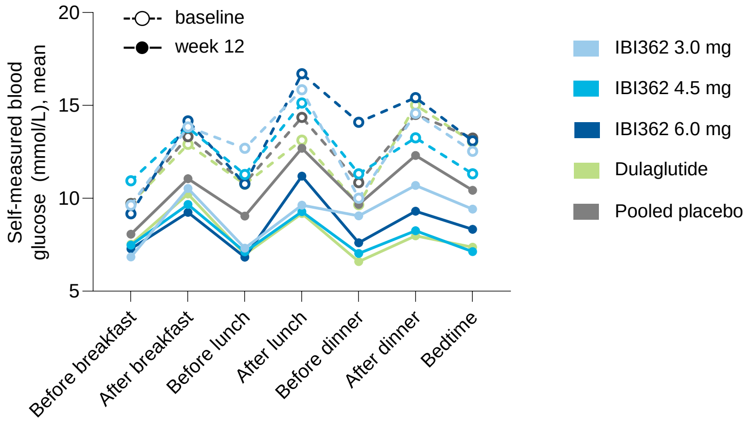

Fig. S6: Seven-point self-measured blood glucose profile at baseline and week 12.

Source data are provided as a Source Data file.

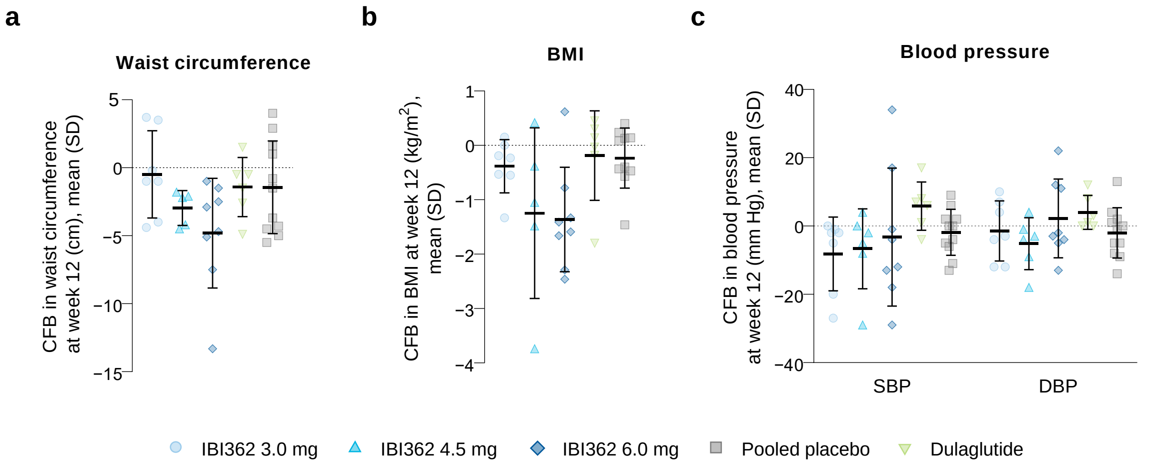

Fig. S7: Change from baseline in waist circumference, BMI and blood pressure at week 12.

a. CFB in waist circumference at week 12**. b.** CFB in BMI at week 12**. c.** CFB in blood pressure at week 12**.** Data are plotted as means +/- SD. **a.** IBI362 3.0 mg *n* = 7; IBI362 4.5 mg *n* = 5; IBI362 6.0 mg *n* = 8; Pooled placebo *n* = 11; Dulaglutide *n* = 6. **b**. IBI362 3.0 mg *n* = 7; IBI362 4.5 mg *n* = 5; IBI362 6.0 mg *n* = 8; Pooled placebo *n* = 10; Dulaglutide *n* = 6. **c**. IBI362 3.0 mg *n* = 7; IBI362 4.5 mg *n* = 6; IBI362 6.0 mg *n* = 8; Pooled placebo *n* = 11; Dulaglutide *n* = 6. BMI = body-mass index; CFB = change from baseline; DBP = diastolic blood pressure; SBP = systolic blood pressure; SD = standard deviation. Source data are provided as a Source Data file.

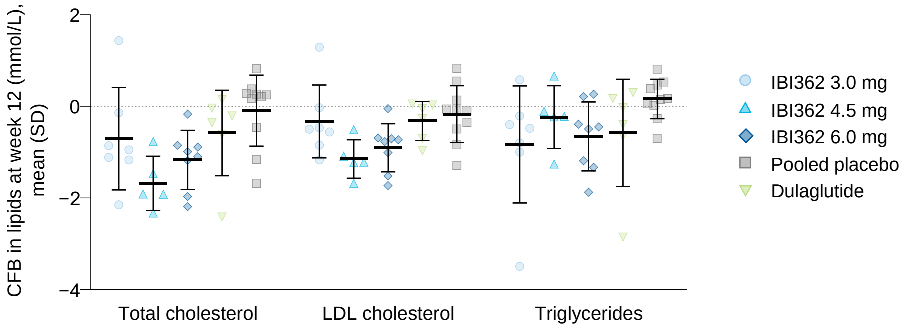

Fig. S8: Change from baseline in lipids levels at week 12.

Data are plotted as means +/- SD. IBI362 3.0 mg *n* = 7; IBI362 4.5 mg *n* = 5; IBI362 6.0 mg *n* = 8; Pooled placebo *n* = 10; Dulaglutide *n* = 6. CFB = change from baseline; LDL = low density lipoprotein; SD = standard deviation. Source data are provided as a Source Data file.

Supplementary Tables

	IBI362 3.0 mg (*n* = 8)	IBI362 4.5 mg (*n* = 8)	IBI362 6.0 mg (*n* = 8)
C_{max} (ng/mL)	89.5 (23.8)	109.1 (22.7)	170.2 (17.4)
T_{max} (h)	48.2 (47.2-119.8)	72.2 (23.3-144.2)	84 (23.8-119.4)
$AUC_{0\text{-}168h}$ (ng·h/mL)	12265.9 (22.6)	16474.2 (29.4)	22135.6 (9.8)

Data are geometric mean (CV%) for C_{max} and AUC, median (range) for T_{max}. $AUC_{0\text{-}168h}$ = area under the concentration versus time curve from time zero to 168 hours; C_{max} =maximum observed drug concentration; CV% = coefficient of variation; N = number of subjects; T_{max} = time of C_{max}.

Table S1: Non-compartmental pharmacokinetic parameters following the first dose

	IBI362 3.0 mg (*n* = 8)	IBI362 4.5 mg (*n* = 8)	IBI362 6.0 mg (*n* = 8)	Dulaglutide (*n* = 6)	Pooled placebo (*n* = 12)
**HbA_{1c}**					
CFB at week 12 (%), LS mean (SE)	−1.46 (0.43)	−2.23 (0.43)	−1.66 (0.41)	−1.98 (0.48)	−0.87 (0.34)
ETD versus placebo, LS mean (90% CI)	−0.59 (−1.52, 0.35)	−1.35 (−2.30, −0.41)	−0.79 (−1.71, 0.13)	−1.11 (−2.12, −0.10)	
p value	0.2941	0.0209	0.1562	0.072	
**Fasting plasma glucose**					
CFB at week 12 (mmol/L), LS mean (SE)	−3.38 (0.49)	−3.82 (0.53)	−4.07 (0.48)	−3.85 (0.55)	−2.52 (0.40)
ETD versus placebo, LS mean (90% CI)	−0.85 (−1.92, 0.21)	−1.30 (−2.42, −0.18)	−1.54 (−2.60, −0.49)	−1.32 (−2.47, −0.17)	
p value	0.185	0.058	0.0185	0.06	
**Body weight**					
Percent CFB at week 12 (%), LS mean (SE)	−0.9 (1.3)	−5.0 (1.3)	−5.4 (1.2)	−0.9 (1.4)	−1.1 (1.0)
ETD versus placebo, LS mean (90% CI)	0.1 (−2.6, 2.8)	−4.0 (−6.7, −1.2)	−4.4 (−7.0, −1.7)	0.2 (−2.8, 3.1)	
p value	0.9434	0.0204	0.0093	0.9139	

LS means, SE and 90% CI are from an MMRM model. All p values are based on two-sided *t*-test from the MMRM model and not adjusted for multiple comparisons. CFB = change from baseline; CI = confidence interval; ETD = estimated treatment difference; HbA_{1c} = glycated haemoglobin A_{1c}; LS = least squares; SE = standard error of the mean.

Table S2: Main efficacy endpoints

MedDRA system organ class<br>Preferred term*	IBI362<br>3.0 mg (*n* = 8)	IBI362<br>4.5 mg (*n* = 8)	IBI362<br>6.0 mg (*n* = 8)	Dulaglutide<br>(*n* = 6)	Pooled placebo<br>(*n* = 12)
**Any treatment-emergent adverse event**	**6 (75.0)**	**8 (100)**	**7 (87.5)**	**4 (66.7)**	**9 (75.0)**
**Gastrointestinal disorders**	**2 (25.0)**	**5 (62.5)**	**4 (50.0)**	**2 (33.3)**	**2 (16.7)**
Diarrhoea	2 (25.0)	3 (37.5)	2 (25.0)	2 (33.3)	0
Nausea	1 (12.5)	1 (12.5)	2 (25.0)	1 (16.7)	1 (8.3)
Gastrooesophageal reflux disease	0	2 (25.0)	0	0	1 (8.3)
Abdominal distension	0	2 (25.0)	0	0	0
Vomiting	0	1 (12.5)	0	1 (16.7)	0
Toothache	0	1 (12.5)	0	0	0
Abdominal discomfort	0	0	1 (12.5)	0	0
Dyspepsia	0	0	0	0	1 (8.3)
**Metabolism and nutrition disorders**	**3 (37.5)**	**4 (50.0)**	**3 (37.5)**	**2 (33.3)**	**3 (25.0)**
Decreased appetite	0	4 (50.0)	2 (25.0)	1 (16.7)	0
Hypokalaemia	1 (12.5)	0	2 (25.0)	0	1 (8.3)
Hypoglycaemia	1 (12.5)	0	1 (12.5)	0	0
Dyslipidaemia	1 (12.5)	0	0	0	0
Hyperuricaemia	0	1 (12.5)	0	1 (16.7)	1 (8.3)
Hypertriglyceridaemia	1 (12.5)	0	0	0	0
Hypercholesterolaemia	0	1 (12.5)	0	0	0
Diabetic ketosis	0	0	0	0	1 (8.3)
**Cardiac disorders**	**2 (25.0)**	**1 (12.5)**	**2 (25.0)**	**1 (16.7)**	**1 (8.3)**
Atrioventricular block first degree	0	0	2 (25.0)	0	0
Myocardial ischaemia	2 (25.0)	0	0	0	0
Ventricular extrasystoles	0	1 (12.5)	0	1 (16.7)	0
Arrhythmia	1 (12.5)	0	0	0	0
Palpitations	0	0	0	1 (16.7)	1 (8.3)
Sinus bradycardia	0	0	0	1 (16.7)	0
Sinus arrhythmia	0	0	0	1 (16.7)	0
**Infections and infestations**	**1 (12.5)**	**2 (25.0)**	**1 (12.5)**	**0**	**1 (8.3)**
Upper respiratory tract infection	0	2 (25.0)	0	0	0
Urinary tract infection	1 (12.5)	0	1 (12.5)	0	0
Bacterial vulvovaginitis	1 (12.5)	0	0	0	0
Gingival abscess	0	0	0	0	1 (8.3)
**Investigations**	**0**	**1 (12.5)**	**2 (25.0)**	**0**	**3 (25.0)**
Lipase increased	0	0	2 (25.0)	0	1 (8.3)
QRS axis abnormal	0	1 (12.5)	0	0	0
Protein urine present	0	0	0	0	1 (8.3)
Muscle enzyme increased	0	0	0	0	1 (8.3)
**General disorders and administration site conditions**	**1 (12.5)**	**0**	**1 (12.5)**	**1 (16.7)**	**0**
Asthenia	0	0	1 (12.5)	0	0
Chest discomfort	1 (12.5)	0	0	1 (16.7)	0
**Respiratory, thoracic and mediastinal disorders**	**1 (12.5)**	**1 (12.5)**	**0**	**0**	**1 (8.3)**
Dysphonia	0	1 (12.5)	0	0	0
Cough	0	1 (12.5)	0	0	0
Pulmonary mass	1 (12.5)	0	0	0	0
Laryngeal pain	0	0	0	0	1 (8.3)
**Musculoskeletal and connective tissue disorders**	**0**	**1 (12.5)**	**0**	**1 (16.7)**	**1 (8.3)**
Periarthritis	0	1 (12.5)	0	0	0
Intervertebral disc protrusion	0	0	0	0	1 (8.3)
Back pain	0	0	0	1 (16.7)	0
**Nervous system disorders**	**0**	**1 (12.5)**	**0**	**0**	**2 (16.7)**
Migraine	0	1 (12.5)	0	0	0
Neuropathy peripheral	0	1 (12.5)	0	0	0
Dizziness	0	0	0	0	1 (8.3)
Facial paralysis	0	0	0	0	1 (8.3)
**Reproductive system and breast disorders**	**1 (12.5)**	**0**	**0**	**0**	**0**
Scrotal dermatitis	1 (12.5)	0	0	0	0
**Skin and subcutaneous tissue disorders**	**0**	**0**	**1 (12.5)**	**0**	**0**
Rash	0	0	1 (12.5)	0	0
**Hepatobiliary disorders**	**1 (12.5)**	**0**	**0**	**0**	**0**
Hepatic function abnormal	1 (12.5)	0	0	0	0
Cholecystitis	1 (12.5)	0	0	0	0
Cholelithiasis	1 (12.5)	0	0	0	0
**Renal and urinary disorders**	**0**	**0**	**1 (12.5)**	**0**	**0**
Proteinuria	0	0	1 (12.5)	0	0
**Immune system disorders**	**0**	**0**	**0**	**0**	**1 (8.3)**
Food allergy	0	0	0	0	1 (8.3)
**Eye disorders**	**0**	**0**	**0**	**0**	**1 (8.3)**
Meibomianitis	0	0	0	0	1 (8.3)
**Ear and labyrinth disorders**	**0**	**0**	**0**	**0**	**1 (8.3)**
Ear pain	0	0	0	0	1 (8.3)

Data are presented as number of patients (%).
By the Medical Dictionary for Regulatory Activities (version 24.0) system organ class and preferred term

Table S3: All treatment-emergent adverse events

Supplementary Note 1:

Clinical Study Protocol

Parts of the study protocol have been redacted as the protocol includes unpublished data.

Clinical Study Protocol

**Study Title:**	A Multiple Dose Study to Assess the Tolerability, Pharmacokinetics and Pharmacodynamics of IBI362 in Chinese Patients with Type 2 Diabetes and Poor Glycemic Control
**Protocol Number:**	CIBI362A101
**Version number and date:**	V1.1/Jan 15, 2021
**Product name:**	Glucagon-like peptide-1/glucagon receptor dual agonist
**Study Phase:**	Phase 1b
**Sponsor:**	Innovent Biologics<br>168 Dongping Street, Suzhou Industrial Park, Jiangsu Province, China
**Sponsor Contact:**	Lei Qian, 021-31837215

Confidentiality Statement

This document contains confidential information of Innovent Biologics, Inc. The contents of this document may not be disclosed to anyone other than the investigator (s), study advisor (s) or related personnel, and institutional review board/independent ethics committee. The information in this document may not be used for any purpose other than the evaluation or conduct of this clinical investigation without the written permission of the sponsor.

Investigator Signature Page

Study Title: A Multiple Dose Study to Assess the Tolerability, Pharmacokinetics and Pharmacodynamics of IBI362 in Chinese Patients with Type 2 Diabetes and Poor Glycemic Control

Protocol No.: CIBI362A101

This protocol is a trade secret of Innovent Biologics (Suzhou) Co., Ltd. I have read and fully understand this protocol and commit to conduct this study in accordance with the requirements of this protocol and Good Clinical Practice, and comply with applicable laws and regulations, as well as the Declaration of Helsinki. Meanwhile, I promise not to disclose any confidential information in this study to any third party without the written consent of Innovent Biologics (Suzhou) Co., Ltd.

Guidance for Investigator: Please sign and date this signature page, print the Investigator's name, title and name of site conducting the study and return to Innovent Biologics (Suzhou) Co., Ltd. after signing.

I have read all the contents of this study protocol and promise to carry out this study as required:

Signature of Investigator:

Printed Name:

Title of Investigator:

Name/Address of Site:

Sponsor Signature Page

Study Title: A Multiple Dose Study to Assess the Tolerability, Pharmacokinetics and Pharmacodynamics of IBI362 in Chinese Patients with Type 2 Diabetes and Poor Glycemic Control

Project No.: CIBI362A101

[redacted]	[redacted]	[redacted]	[redacted]
[redacted]	[redacted]	____________________	____________________
[redacted]	[redacted]	____________________	____________________

Synopsis

**Protocol no.**	CIBI362A101
**Sponsor**	Innovent Biologics
**Study drug**	IBI362
**Active ingredient**	Glucagon-like peptide-1/glucagon receptor dual agonist
**Study title**	A multiple dose study to assess the tolerability, pharmacokinetics and pharmacodynamics of IBI362 in Chinese patients with type 2 diabetes and poor glycemic control
**Study phase**	Phase 1b
**Study objectives**	**Primary objectives:**<br>• To investigate the safety and tolerability of multiple subcutaneous injections of IBI362 in patients with type 2 diabetes inadequately controlled by lifestyle intervention or metformin, and to determine the safe dose range.<br>**Secondary objectives:**<br>• To investigate the pharmacokinetic/ pharmacodynamics (PK/PD) parameters of multiple subcutaneous injections of IBI362 in patients with type 2 diabetes inadequately controlled by lifestyle intervention or metformin.
**Study design**	This study will be the first to evaluate the safety, tolerability and PK/PD of IBI362 administered as multiple injections in Chinese patients with type 2 diabetes. A multicenter, randomized, double-blind, placebo-controlled trial design will be used. The investigators and patients are blinded to the study drug IBI362 and placebo, and dulaglutide will be set as an open-label active reference. The study will be planned to include 42 patients with type 2 diabetes who failed to control HbA1c after at least 2 months of lifestyle intervention or stable dose of metformin (≥1000 mg/day or maximum tolerated dose). The study will be divided into three cohorts, which will be Cohort 1 (n = 14), Cohort 2 (n = 14) and Cohort 3 (n = 14). Patients in each cohort will be randomized 8:4:2 to IBI362 group (n = 8), placebo group (n = 4), or dulaglutide 1.5 mg group (n = 2). The active control, dulaglutide, will be administered as 1.5 mg once-weekly for 12 weeks in Cohorts 1, 2 and 3, and the dosing schedules for IBI362 and placebo are described below:<br>**Cohort 1:** Patients randomized to IBI362 or placebo initiate treatment with 1.0 mg once-weekly with dose escalations every 4 weeks by 1 mg until the maintenance dose (3.0 mg) will be achieved.

	**Cohort 2:** Patients randomized to IBI362 or placebo initiate treatment with 1.5 mg once-weekly with dose escalations every 4 weeks by 1.5 mg until the maintenance dose (4.5 mg) will be achieved.<br>If 3.0 mg or 4.5 mg is not tolerated in Cohort 2, the dose should be adjusted according to the criteria in Table 1.<br>**Cohort 3:** Dosing should not be started until the patients in Cohort 2 complete the 4-week tolerability evaluation of 1.5 mg. If 1.5 mg is not well-tolerated, 2.0 mg and higher doses in Cohort 3 will not be explored. Patients randomized to IBI362 or placebo initiate treatment with 2.0 mg once-weekly with dose escalations every 4 weeks by 2.0 mg until the maintenance dose (6.0 mg) will be achieved.<br>The study will consist of a 3-week screening period, a 12-week double-blind treatment period, and an 8-week follow-up period.<br>**Discontinuation Criteria: If any of the following criteria are met, the investigator and the sponsor should immediately initiate a discussion to decide whether to terminate dose escalation.**<br>• Occurrence of at least one serious treatment-emergent adverse event related to IBI362 (except for expected adverse events related to the pharmacological properties of IBI362, such as hypoglycemia, etc.).<br>• No less than 4 patients experience symptomatic hypoglycemic events (plasma glucose levels < 3.0 mmol/L) at a specific dose level, and those events are considered to be related to IBI362.<br>• No less than 2 patients receiving IBI362 experience persistent (more than one week) symptoms of acute pancreatitis.<br>• No less than 2 patients experience severe IBI362-related, non-serious adverse events at the same dose, regardless of whether the event is associated with the same organ or system.
**Sample size**	42 patients
**Inclusion criteria**	1. A diagnosis of type 2 diabetes, as defined by WHO (1999) for at least 6 months.<br>2. Male or female, aged 18 to 75 years at signing informed consent.<br>3. Blood glucose will be inadequately controlled by lifestyle intervention alone or with stable dose of metformin (≥ 1000 mg/day or maximum tolerated dose) within 2 months prior to screening.<br>4. 7.5% ≤HbA1c≤11.0% by local laboratory at screening.<br>5. $20 \leq$ BMI ≤ 35 kg/m ^{2} (BMI = weight (kg)/height (m^2)) .<br>6. Maintain a stable diet and regular exercise lifestyle during the study.

	7. Willing to sign the Informed Consent Form (ICF) and able to participate in the trial and comply with all trial requirements.
**Exclusion criteria**	1. Type 1 diabetes, gestational diabetes, or other types of diabetes.<br>2. Ketoacidosis or lactic acidosis within 6 months prior to screening.<br>3. History of severe hypoglycemic episodes within 6 months prior to screening, defined as symptoms of neuroglycopenia that require supportive care from others, or complete ignorance of hypoglycemia or lack of awareness of hypoglycemic symptoms. Patients who are unable to communicate and understand the symptoms of hypoglycemia and appropriate treatment should also be excluded from the trial at the discretion of the investigator.<br>4. Acute myocardial infarction, unstable angina pectoris, coronary artery bypass grafting, coronary intervention (except diagnostic angiography), transient ischemic attack (TIA), cerebrovascular accident, acute and chronic heart failure within 6 months before screening.<br>5. Abnormal 12-lead ECG (e.g., QTcF > 450 ms, PR interval < 120 ms, or PR interval > 220 ms, second-degree and third-degree atrioventricular block, ventricular conduction delay, i.e., QRS > 120 ms, right bundle branch block, left bundle branch block, Wolff-Parkinson-White syndrome) at screening that increase the risk of the patient or may cause confusion in the analysis of ECG data (QT); or taking any drug that may affect the QT interval at the discretion of the investigator, e.g., antiarrhythmic drugs class IA and III, cisapride, macrolide antibiotics and psychotropic drugs (phenothiazines [pyridazine, chlorpromazine, pyridazine], butyrophenones [droperidol, haloperidol] and loperamide).<br>6. Previous diagnosis of long QT syndrome.<br>7. Uncontrolled blood pressure at screening with systolic blood pressure > 140 mmHg or < 90 mmHg and diastolic blood pressure > 90 mmHg or < 50 mmHg.<br>8. Heart rate < 50 bpm or > 90 bpm at screening.<br>9. Active or untreated malignancy within 5 years prior to screening, or in clinical remission of malignancy (except for patients with basal and squamous cell carcinoma of the skin, carcinoma in situ of the cervix, and papillary thyroid carcinoma without postoperative recurrence).<br>10. Previous history of acute or chronic pancreatitis, or serum lipase/amylase > 2 × upper limit of normal at screening, or fasting triglycerides > 5.65 mmol/L (500 mg/dl). If the patient is on lipid-

	modifying therapy, the dose must be stable for 30 days prior to screening.<br>11. Clinically symptomatic liver disease, acute or chronic hepatitis, or transaminases (ALT and AST) and alkaline phosphatase (ALP) > 2 × upper limit of normal and total bilirubin > upper limit of normal at screening.<br>12. Calcitonin ≥ 15 ng/L at screening.<br>13. EGFR < 60 mL/min/1.73 m^2 at screening, estimated using the modified MDRD formula: eGFR = 175 × [(serum creatinine (μmol/L)/88.4)] $^{-1\ 234}$ × [age (years)] $^{-0\ 179}$ × 0.79 (female) or × 1 (male).<br>14. History or presence of psychiatric disorder at screening that would make participation in this study inappropriate at the discretion of the investigator.<br>15. Have Diagnosed of gastroparesis or received any form of obesity surgical procedure, or had abnormal gastric emptying considered clinically significant by the investigator.<br>16. Known history of regular drug abuse.<br>17. Positive human immunodeficiency virus (HIV) antibody and/or HIV infection at screening, or positive syphilis antibody.<br>18. History of hepatitis B and/or positive hepatitis B surface antigen at screening or positive hepatitis C (HCV) antibody at screening.<br>19. Previous diagnosis of Gilbert's syndrome.<br>20. International normalized ratio (INR) of prothrombin time greater than the upper limit of normal at screening.<br>21. History of medullary thyroid C-cell carcinoma, MEN (multiple endocrine neoplasia) syndrome 2A or 2B, or relevant family history.<br>22. Diagnosed with autonomic neuropathy, manifested as: urinary retention, resting tachycardia, orthostatic hypotension, and diabetic diarrhea.<br>23. Significant weight change with a percentage change > 5% within 3 months prior to screening.<br>24. Donation of ≥ 400 mL of blood or excessive blood loss or bone marrow transplantation within 3 months before screening, or presence of hemoglobinopathy, hemolytic anemia, sickle cell anemia, or hemoglobin < 110 g/L (men) or < 100 g/L (women).<br>25. Hyperthyroidism or hypothyroidism confirmed by clinical assessment and/or TSH abnormality that, in the opinion of the investigator, may increase the risk of the patient.

	26. Use of antidiabetic drugs other than metformin within 2 months prior to screening.<br>27. Use or planned to use weight-lowering medications such as Liraglutide, Orlistat, Sibutramine HCl, Phenylpropanolamine, Chlorphenindole, Phenylbutamine, Chlorocarbazone HCl, Fintramine, Fintramine/Topyrate, Bupropion, Naltrexone/Bupropion within 3 months prior to screening during the trial.<br>28. Chronic use of glucocorticoids (cumulative or continuous use > 2 weeks) within 1 year before screening, or use of glucocorticoids (excluding topical, intraocular, intranasal, intra-articular, inhalation) within 4 weeks before screening.<br>29. Ongoing central nervous stimulant use (e.g., methylphenidate hydrochloride) at screening, with the exception of caffeinated beverages.<br>30. Known history of hypersensitivity to study drugs or drug components.<br>31. Participation in a clinical trial of any drug or medical device within 3 months prior to screening (randomized and received medication).<br>32. Female with childbearing potential, except those who are sterilized or amenorrheic, who are unwilling to inform their sexual partners of their participation in this clinical study and do not take effective contraceptive measures during the study. Male who are unwilling to inform their female sexual partners of the participation in the clinical study and unwilling to take effective contraceptive measures during the study.<br>33. Pregnant or lactating women or women who are preparing for pregnancy or breastfeeding during the study period<br>34. Any clinically significant abnormal laboratory value that may interfere with the interpretation of efficacy and safety data at the discretion of the investigator in this study.<br>35. Patients with any other factors that may affect the cooperation, efficacy, or safety evaluation at the discretion of the investigator, such as the presence of psychiatric disorders.<br>36. Average weekly alcohol intake of more than 21 units for men and 14 units for women, or unwillingness to stop drinking 24 hours before the day of medication and throughout the study (1 unit = 360 ml of beer, or 150 ml of red wine, or 45 ml of distilled spirits/liquor).
**Study drug, dose, and regimen**	**IBI362 and placebo: 2 mg/vial**<br>**Dulaglutide: 1.5 mg/vial;**

	**Mode of administration: Subcutaneous injection, once-weekly.**<br>• Cohort 1:1.0 mg for 4 weeks + 2.0 mg for 4 weeks + 3.0 mg for 4 weeks;<br>• Cohort 2:1.5 mg for 4 weeks + 3.0 mg for 4 weeks + 4.5 mg for 4 weeks;<br>• Cohort 3:2.0 mg for 4 weeks + 4.0 mg for 4 weeks + 6.0 mg for 4 weeks;<br>• Active control: Dulaglutide 1.5 mg QW for 12 weeks.
**Endpoints**	**Primary endpoints:**<br>• **Safety and tolerability:** During dose escalation, the incidence and severity of adverse events at each dose level, including chief complaint, physical examination, laboratory tests (hematology, blood chemistry, lipid panel, coagulation function, urinalysis, myocardial enzymes, serum amylase and/or lipase, thyroid function, calcitonin, etc.), vital signs (pulse, respiration, blood pressure, body temperature), 12-lead ECG and other examinations; treatment compliance; and record the name, clinical characteristics, severity, start and stop time, supportive care and outcomes of adverse events, correlation with the study drugs;<br>• **Maximum tolerated dose:** If the dose exploration is stopped when the criteria for stopping dose escalation are met at a certain dose level, the previous dose is the maximum tolerated dose. If the criteria are still not met when the maximum exploratory dose is reached, the maximum tolerated dose is greater than or equal to the maximum escalation dose.<br>**Secondary endpoints:**<br>• To evaluate the pharmacokinetic parameters of IBI362 in patients with type 2 diabetes, including but not limited to: time to maximum concentration (T_{max}), maximum concentration (C_{max}), area under the curve (AUC), volume of distribution (Vd), half-life ($T_{1/2}$), clearance (CL), accumulation ratio (AR), etc.;<br>• To assess the pharmacodynamics parameters of IBI362 after multiple doses: changes of fasting blood glucose, glucagon, insulin, fasting C-peptide, hemoglobin A1c (HbA1c), oxytomodulin (OXM) and GLP-1 after administration;<br>• Changes of fasting blood glucose, insulin and glucagon at steady state in each dose level during dose escalation;<br>• Changes from baseline in each parameter of MTT test after multiple dose administration;<br>• Changes of self-measure blood glucose after multiple doses;

	• The incidence of anti-IBI362 antibody (ADA) and neutralizing antibody (NAb) in serum before and after administration.<br>**Exploratory endpoints:**<br>• To evaluate the effect of IBI362 on weight loss (changes in fasting body weight, waist circumference and BMI);<br>• To assess the improvement of the pancreatic function: changes in homeostatic model assessment (HOMA) of β-cell function and insulin resistance (IR).
**Statistical methods**	**This study is a multicenter, randomized, double-blind, placebo-controlled study, with dulaglutide as an active control.**<br>**Sample Size:**<br>The primary objective of this study is to preliminarily evaluate the safety and tolerability of different doses of IBI362. No formal statistical hypothesis testing or formal sample size estimation are performed. Enrollment is planned in 3 cohorts. A total of 14 patients are planned to be enrolled in each cohort and randomized at a ratio of 8:4:2 to IBI362, placebo, and active control.<br>**Efficacy Analysis:**<br>In general, descriptive statistical analysis will be performed for efficacy endpoints. Continuous efficacy variables will be describe using means and 95% confidence intervals for patients receiving IBI362, placebo and active control in each cohort. A two-sample t-test will be used to calculate and provide the corresponding point estimate and 95% confidence interval of the difference, as well as p-value of IBI362 versus placebo for each dose group. LOCF will be used for missing value imputation. For the categorical efficacy endpoints, the ratio and their 95% confidence interval within each cohort will be calculated using Clopper-Pearson; the difference between IBI362 and placebo will be compared using the chi-square test and the corresponding 95% confidence interval will be calculated. Missing values for categorical endpoints will be imputed using the default non-response method.<br>**Safety analysis:**<br>All AEs will be classified according to Medical Dictionary for Regulatory Activities (MedDRA) system organ class, MedDRA preferred term and adverse event grade. The number and percentage of patients with adverse events in each category (including causality, severity, SAE, etc.) will be summarized, and events in each category will be further summarized by MedDRA system organ class and preferred term.<br>**Laboratory tests:**

	Hematology, blood chemistry, lipid panel, coagulation function, urinalysis, myocardial enzymes, serum amylase and/or lipase, thyroid function, calcitonin, vital signs (pulse, respiration, blood pressure, body temperature), 12-lead ECG and other indicators will be summarized using mean ±standard deviation, maximum, minimum and median to describe measured values and changes before and after treatment. Cross table will be used to describe normal and abnormal changes before and after treatment.<br>Urinalysis: cross table will be used to describe normal and abnormal changes before and after treatment.<br>The proportion of patients with clinically significant abnormal changes will be described, and whether the abnormality is clinically significant will be judged by the investigator.<br>**Pharmacokinetics and pharmacodynamics:**<br>PK parameters, including but not limited to T_{max}, C_{max}, AUC, Vd, $T_{1/2}$, CL, and AR, will be summarized for each cohort separately.<br>Pharmacodynamics parameters will be summarized, including: fasting blood glucose, glucagon, insulin, fasting C-peptide, HbA1c, endogenous OXM, and GLP-1 measurements at baseline and at various time points after drug administration, and changes from baseline at each time point will be summarized.<br>**Exploratory Analyses:**<br>Changes from baseline in HOMA-β, HOMA-IR, fasting weight, waist circumference, and BMI will be summarized at each time point after treatment.

Study Design

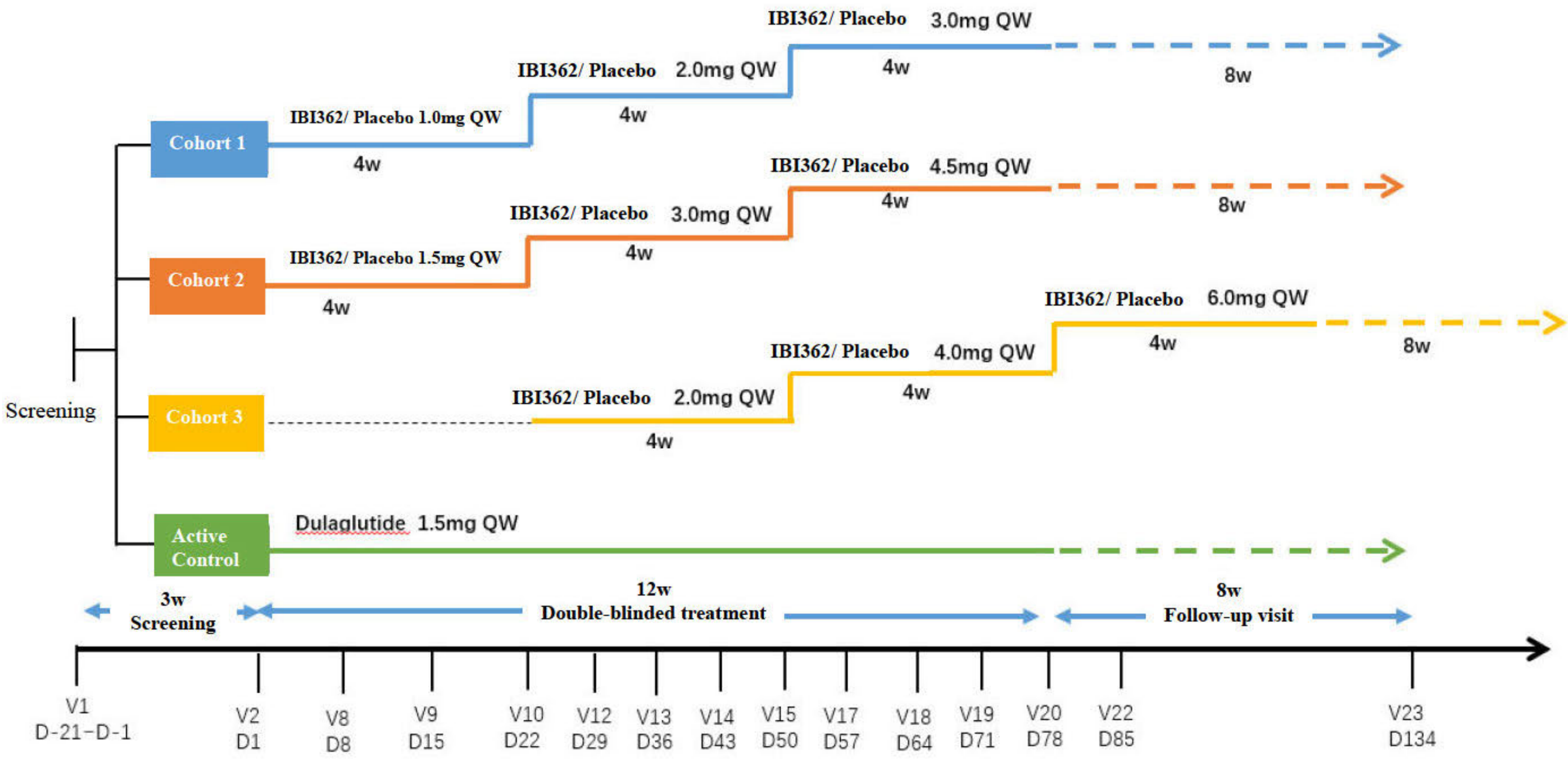

Table 1. Dose adjustment reference table in escalation process

**Weeks**		**1**	**2**	**3**	**4**	**5**	**6**	**7**	**8**	**9**	**10**	**11**	**12**				
Cohort 1 (N = 12)	IBI362/Placebo	1.0 mg QW				2.0 mg QW				3.0 mg QW							
	Dulaglutide	1.5 mg QW															
**Weeks**		**1**	**2**	**3**	**4**	**5**	**6**	**7**	**8**	**9**	**10**	**11**	**12**				
Cohort 2 (N = 12)	IBI362/Placebo	1.5 mg QW				3 mg QW				4.5 mg QW							
	Dulaglutide	1.5 mg QW															
**Weeks**		**1**	**2**	**3**	**4**	**5**	**6**	**7**	**8**	**9**	**10**	**11**	**12**				
Cohort 2 (backup 1) *	IBI362/Placebo	1.5 mg QW				3 mg	Discontinued	2.25 mg	2.25 mg	3 mg QW							
**Weeks**		**1**	**2**	**3**	**4**	**5**	**6**	**7**	**8**	**9**	**10**	**11**	**12**				
Cohort 2 (backup 2) #	IBI362/Placebo	1.5 mg QW				3 mg QW				4.5 mg	Discontinued	3.75 mg	3.75 mg				
**Weeks**		**- 4**	**- 3**	**- 2**	**- 1**	**1**	**2**	**3**	**4**	**5**	**6**	**7**	**8**	**9**	**10**	**11**	**12**
Cohort 3 & (N = 12)	IBI362/Placebo					2.0 mg QW				4.0 mg QW				6.0 mg			
	Dulaglutide					1.5 mg QW											

Notes:

* If patients in Cohort 2 cannot tolerate 3.0 mg during dose escalation, the dose should be reduced to 2.25 mg after 1-week break in Cohort 2 (backup1), and subsequent dose escalation should be performed as Cohort 2 (backup1);

# If patients in Cohort 2 cannot tolerate 4.5 mg during the escalation, the dose should be discontinued for 1 week and reduced to 3.75 mg in Cohort 2 (backup2), and the dose should be maintained until the end of the trial;

& Patients in Cohort 3 should not start until patients in Cohort 2 complete the 4-week tolerance study at 1.5 mg. If patients in Cohort 2 cannot tolerate 1.5 mg, patients in Cohort 3 will not explore 2.0 mg or higher.

Intolerance criteria for a single patient:

1. If venous plasma glucose < 3.0 mmol/L or venous plasma glucose does not reach the criteria of < 3.0 mmol/L, but the symptoms of hypoglycemia are obvious and cannot be resolved spontaneously, repeated measurement of blood glucose is required before the next dose. If the criteria are still met, the patient is considered to be intolerant at this dose;
2. Other severe intolerance considered by the investigator to be related to the study drug occurred, and the investigator consider it necessary to stop the trial after discussion with the sponsor, and at this case the patient is considered to be intolerable at this dose.

Table 2. Schedule of Activities

Stage	Screening Period[1]	Baseline	Treatment Period[2]																					Safety Follow-up	Early Termination
Study duration (days)	- 21 to -2	- 1	1	2	3	4	5	6	8	15	22	24	29	36	43	50	52	57	64	71	78	80	85	134	
Study duration (weeks)	- 3 to - 1	- 1	1						2	3	4		5	6	7	8		9	10	11	12		13	20	
Window (days)	/		/	/	/	/	/	/	±2	±2	±2	/	±2	±2	±2	±2	/	±2	±2	±2	±2	/	±2	±3	
Informed Consent	X																								
Inclusion/ exclusion criteria	X																								
Demographics/ past medical history/ previous treatment	X																								
Vital Signs[3]	X	X	X	X	X	X	X	X	X	X	X	X	X	X	X	X	X	X	X	X	X	X	X	X	X
Physical Examination[4]	X		X						X	X	X		X	X	X	X		X	X	X	X		X		X
Height	X																								
Weight[5]	X	X	X										X					X					X		X
Waist circumference[6]	X	X	P						P	P	P		P	P	P	P		P	P	P	P		X		X
Laboratory Tests[7]	X									X			X					X					X		X
12-lead ECG[8]	X	X	P, 12h	X	X	X			P	P	P	X	P, 12h	P	P	P	X	P, 12h	P	P	P, 12h	X	X		X
Serum Virology[9]	X																								
Serum calcitonin	X																						X		X
Blood/urine pregnancy test[10]	X												X					X					X		X
Thyroid Function[11]	X																								
SMG training, distribution of patient diary cards and glucometer[12]	X																								
Seven point fingertip capillary blood glucose[13]		X											X					X					X		

Stage	Screening Period [1]	Baseline	Treatment Period [2]																				Safety Follow-up	Early Termination
Study duration (days)	- 21 to -2	- 1	1	2	3	4	5	6	8	15	22	24	29	36	43	50	52	57	64	71	78	80	85	134	
Study duration (weeks)	- 3 to - 1	- 1	1						2	3	4		5	6	7	8		9	10	11	12		13	20	
Window (days)	/		/	/	/	/	/	/	±2	±2	±2	/	±2	±2	±2	±2	/	±2	±2	±2	±2	/	±2	±3	
Glycosylated hemoglobin [14]	X		•										•					•					•		•
Adverse events/concomitant medication	X	X	X	X	X	X	X	X	X	X	X	X	X	X	X	X	X	X	X	X	X	X	X	X	X
Study Drug Administration			X						X	X	X		X	X	X	X		X	X	X	X				
Immunogenicity [15]			•										•					•					•	•	•
PK sampling [16]			•	•	•	•	•	•	•			•	•	•			•	•	•		•	•	•		•
PD sampling [16]		•			•				•				•					•			•	•	•		•
Infusion Reactions			X						X	X	X		X	X	X	X		X	X	X	X				

Notes:

1. If the interval between the screening examination and first dose is more than 5 days, re-confirmation should be performed within 5 days before dosing, including vital signs, physical examination, hematology, blood biochemistry, coagulation function, urinalysis, blood lipid, ECG and pregnancy test.
2. The dose should be adjusted according to the patient's tolerance throughout the treatment period. See the dose escalation adjustment table for details.
3. Vital Sign measurements should be completed within 2 hours prior to administration on the day of the visit.
4. Physical examination: Skin and mucosa should be observed for abnormalities at the injection site after drug administration. Other than the examination time points listed in the table, the local skin and mucosa at the injection site should be observed for abnormalities, including but not limited to skin erythema/redness, swelling, pain/tenderness, congestion/bleeding, at immediately, 20 minutes, 40 minutes and 60 minutes after administration.
5. Body weight: The patients should wear single clothes after fasting and defecation during each measurement. The same patient should use the same weight scale each time and avoid strenuous exercise before measurement.
6. Waist circumference should be measured before administration (P), measure the circumference of the midpoint line between the lowest rib and the two horizontal lines at the upper edge of iliac crest. Patients stood with their feet together, arms on both sides of the body. The patient needs to relax and an end-tidal reading is taken. During the treatment period, each measurement will be duplicated, and the average of the two measurements will be recorded in the eCRF as the source document. Try to use the same flexible ruler for the same patient at each time, avoid overstretching the flexible ruler and measuring with clothing.
7. Laboratory tests include:

1) Hematology: white blood cell count (WBC), red blood cell count (RBC), platelet (PLT), hemoglobin concentration (HGB), hematocrit (HCT), white blood cell differential count (neutrophils, basophils, eosinophils, monocytes and lymphocytes);
2) Blood biochemistry: aspartate aminotransferase (AST), alanine aminotransferase (ALT), total bilirubin (TBIL), direct bilirubin (DBIL), albumin (ALB), total protein, glutamyl transpeptidase (GGT), alkaline phosphatase (ALP), lactate dehydrogenase (LDH), serum potassium, serum sodium, serum calcium, serum chloride, uric acid (UA), urea, creatinine (Cr);

3) Blood lipids: total cholesterol (TC), triglyceride (TG), high-density lipoprotein cholesterol (HDL-C), low-density lipoprotein cholesterol (LDL-C);
4) Coagulation function: prothrombin time (PT), activated partial thromboplastin time (APTT), international normalized ratio (INR);
5) Urinalysis: urine pH, urine protein, urine glucose, urine red blood cells, urine white blood cells;
6) Myocardial enzyme spectrum: creatine kinase (CK), creatine kinase isoenzyme (CK-MB);
7) Amylase and lipase;

8. 12-lead electrocardiogram (ECG): For Day (D) 1/D29/D57/D78 visits, ECG should be performed once before administration (P) and at 12 hours ± 30 minutes after administration, and for other visits, ECG should be performed only once before administration or on the day of visit. Any clinically significant findings from ECGs observed by the investigator may additionally perform ECG per the actual situation.
9. Serum virology: including human immunodeficiency virus (HIV) antibody, hepatitis B (5 items), hepatitis C (HCV) antibody and syphilis antibody.
10. Serum pregnancy test is preferred and urine pregnancy may be substituted if the site is unable to do so.
11. Thyroid function parameters include: thyroid stimulating hormone (TSH), free T3 (FT3) and free T4 (FT4).
12. Eligible patients will be trained in self-monitoring of blood glucose (SMG), and a patient diary card and glucometer will be dispensed.
13. Seven points fingertip capillary blood glucose refers to glucose collected from 7 finger stick at fasting, 2 hours after breakfast (± 30 min), before lunch, 2 hours after lunch (± 30 min), before dinner, 2 hours after dinner (± 30 min), and at bedtime, and the collection date will be completed within 2 days before D-1, D29, D57, and D85 visits. Fasting state will be defined as a 10-hour fast.
14. Blood samples for HbA1c will be collected at screening, within 1 h prior to dosing on D1 of Week 1, within 1 h prior to dosing on D29 of Week 5, within 1 h prior to dosing on D57 of Week 9, and at 168 h ± 12 h (D85) after dosing on D78 of Week 12. If a patient fails to complete the study and withdraws from the study prematurely, samples will be collected at the early termination visit. Two (2) mL will be collected at each collection point and the detailed procedures will be described in the central laboratory manual.
15. Immunogenicity sample will be collected in Week 1 (within 1 hour before the start of infusion), Week 5 (within 1 hour before infusion on D29), Week 9 (within 1 hour before infusion on D57), Week 13 (168 hours ± 12 hours after Week 12 administration) and safety follow-up visit. If a patient withdraws prematurely. An immunogenicity sample should also be collected at early termination visit as far as possible. Collect 5 mL at each blood collection point. Refer to the central laboratory manual for the detailed operation process.
16. The PK/PD sampling points are detailed in Table 3.
    ● Central laboratory test.

Table 3. PK/PD Sampling Schedule

Weeks of dosing	- 1W	1W									2W	4W	5W			6W	8W	9W			10W	12w		13W
Dosing Days	D-1	D1				D2	D3	D4	D5	D6	D8	D24	D29			D36	D52	D57			D64	D78	D80	D85
Blood sampling time		- Within 1 hour	4h	8h	12h	24h	48h	72h	96h	120h	- Within 1 hour	48h	- Within 1 hour	4h	8h	Within 1 hour	48h	- Within 1 hour	4h	8h	- Within 1 hour	- Within 1 hour	48h	Give the drug for 168 hours
Blood collection time window	-	-	± 10 min	± 20 min	± 30 mi	± 1h	± 2h	± 3h	± 4h	± 5h	-	± 2h	-	± 10 min	± 20 min	-	± 2h	-	± 10 min	± 20 min	-	-	± 2h	± 12h
Pre-dose	√	√									√		√			√		√			√			
Drug administration			√	√	√	√	√	√	√	√		√		√	√		√		√	√				√
PK Sampling 1		●	●	●	●	●	●	●	●	●	●	●	●	●	●	●	●	●	●	●	●	●	●	●
PD Sampling 2							●				●		●					●				●		●
Standard meal test (MTT) 3, 4	●																						●	

Notes:

1. PK sampling time points: Week 1 (intensive PK) PK sampling points: within 1 h before administration, 4 h ± 10 min, 8 h ± 20 min, 12 h ± 30 min, 24 h ± 1 h (Day 2), 48 h ± 2 h (Day 3), 72 h ± 3 h (Day 4), 96 h ± 4 h (Day 5), 120 h ± 5 h (Day 6) and within 1 h before administration on Day 8. Week 4: D22 for 48h ± 2h. Sparse sampling in Week 5: within 1 h before administration on Day 29, 4 h ± 10 min, 8 h ± 20 min after administration and within 1 h before administration on Day 36. Week 8: D50 48h ± 2h. Sparse sampling in Week 9: within 1 h before administration on Day 57, 4 h ± 10 min, 8 h ± 20 min after administration and within 1 h before administration on Day 64. Sparse sampling at Week 12: Within 1h before administration, 48h ± 2h after administration (D80) and 168h ± 12h after administration (D85) on D78. If a patient fails to complete the study and withdraws from the study prematurely, PK samples will be collected at early termination visit. Three (3) mL samples will be collected at each blood collection site and detailed procedures will be provided in the central laboratory manual.

2. PD sampling (insulin/C-peptide/plasma glucose/glucagon/OXM/GLP-1) points: Week 1: within 48 hours ± 2 hours after dosing (Day 3), within 1 hour before dosing on Day 8; Week 5: within 1 hour before dosing on Day 29; Week 9: within 1 hour before dosing on Day 57; Week 12: within 1 hour before dosing on D78 and 168 hours ± 12 hours after dosing (D85). PD samples will be collected at the early termination visit, if a patient withdraw early due to failure to complete the study. All PD sampling will be performed in the fasted state. A total of 9 mL will be collected from each blood collection site, and the detailed operation process will be described in the central laboratory manual.

3. Standard meal test (MTT) collection measures included: insulin, C-peptide, plasma glucose, glucagon, endogenous OXM, and endogenous GLP-1. The sampling points include: fasting, 30 min, 1h, 2h, 3h and 4h. A total of 9 mL blood will be collected from each blood collection point. See the manual of the central laboratory for the detailed operation process. Fasting state refers to fasting for 10 hours. [The standard meal is instant noodles. The master Kang roast beef noodles (bucket noodles) purchased by the sponsor in a unified manner are removed from the oil bag. The noodles are soaked in boiled water and then removed. The seasoning bag is added and mixed well. The time is counted from the first bite of eating. The noodles are eaten within 10 minutes, and 200 mL of water is drunk; or the instant noodles are directly opened with 200 mL of boiled water to ensure that the noodles are eaten and the water or soup is drunk]. Visit days when MTT test is performed: 1 day prior to Cycle 1 administration, Day 80 (D80).

4. If the patient experiences drug-related adverse events during dose escalation and meets the protocol-specified tolerance criteria, dose adjustment is required at the investigator's discretion, and a standard meal test should be performed 48 hours after the first dose after dose adjustment.

● Test by central laboratory

CONTENTS

Synopsis 4

Study Design 12
Table 1. Dose adjustment reference table in escalation process 13
Table 2. Schedule of Activities 14
Table 3. PK/PD Sampling Schedule 17
List of Abbreviations 23

1 Study background 25

1.1 Background 25
1.1.1 GLP-1R/GCGR dual agonist 25
1.1.2 Type 2 diabetes mellitus 26
1.1.3 Type 2 diabetes mellitus complicated with obesity 26
1.2 Study Rationale 27
1.3 Potential Risks and Benefits of Treatment Options 28
1.3.1 Potential risks of IBI362 based on preclinical safety studies 29
1.3.2 Potential risks of IBI362 based on clinical safety studies 29
1.3.3 Potential benefits of IBI362 based on clinical safety studies 32

2 Study objectives and endpoints 33

2.1 Study objectives 33
2.1.1 Primary objective 33
2.1.2 Secondary objectives 33
2.2 Study Endpoints 33
2.2.1 Primary endpoint 33
2.2.2 Secondary endpoints 34
2.2.3 Exploratory Endpoints 34

3 Overall Study Design 34

3.1 Design Rationale 34
3.1.1 Dose Selection Principle 34
3.2 Phase 1b/2 Study Design 36
3.2.1 Overall Study Design 36
3.2.2 Measures to control bias 37
3.2.3 Discontinuation Criteria 37

4 Study Population 37

4.1 Inclusion criteria 37
4.2 Exclusion Criteria 38
4.3 Restrictions during the study 40
4.4 Patient Discontinuation/Withdrawal 40
4.4.1 Treatment discontinuation 40
4.4.2 Withdrawal 41
4.4.3 Withdrawal procedures 42
4.5 Patients Withdrawal 42

5. Study Drug and Other Therapies 42

5.1 Treatment Allocation 42

5.1.1 Treatment allocation 42
5.1.2 Randomization and blinding 42
5.2.1 Physical and Chemical Characteristics of Drug Substance 43
5.2.2 Study Drug Strength and Manufacturer 43
5.2.3 Storage 44
5.2.4 Mode of administration 44
5.3 Concomitant Therapy 44
5.3.1 Prohibited Drugs 44
5.4 Administration During Pregnancy, Childbearing Age, or Lactation 45
5.4.1 Pregnancy 45
5.4.2 Subjects with Childbearing Potential 45
5.4.3 Nursing women 45
5.5 Treatment Compliance 45
5.6 Drug Return and Destruction 45
5.7 Documentation of Study Drug 46
5.8 Complaint handling 46

6 STUDY ASSESSMENTS AND PROCEDURES 47

6.1 Inclusion Procedures 47
6.1.1 Inclusion of patients 47
6.1.2 Screening period and Baseline Period 47
6.2 Double-Blind Treatment Period 48
6.3 Safety Follow-up 48
6.4 Early Withdrawal Visits 48

7 STUDY ASSESSMENTS 49

7.1 Safety and Tolerability Assessments Metrics 49
7.2 Safety and Other Assessments 49
7.2.1 Laboratory Tests 49
7.2.2 Physical examination 51
7.2.3 Vital Signs 52
7.2.4 ECG 52
7.2.5 Fasting Body Weight Measure 53
7.2.6 Pregnancy Testing 53
7.3 PK/PD Measurements 53
7.3.1 PK Blood Sampling Points 53
7.3.2 PD (Insulin/C-peptide/Plasma Glucose/Glucagon/OXM/GLP-1) Blood Collection Points: 54
7.3.3 HbA1c Blood Sampling Points: 54
7.3.4 PK/PD Measurements 55
7.4 Immunogenicity Assessment Indicators 55
7.4.1 Immunogenicity Blood Sampling Points 55
7.4.2 Immunogenicity indicators 55

8. safety reporting and adverse event management 55

8.1 Definition of Adverse Events 55
8.2 Serious Adverse Events Definition of 56
8.3 Adverse Events of Special Interest (AESI) 56
The above listed AESIs need to be reported to the sponsor within 24 hours of awareness (i.e. drugsafety@innoventbio.com), if the AESI meets the SAE criteria

and is also reported according to the SAE reporting process 57
8.3.1 Gastrointestinal Reactions 57
8.3.2 Acute Pancreatitis 58
8.3.3 Hepatic dysfunction events 59
8.3.4 Hypoglycemic Events 60
8.3.5 Severe adverse events that are related to the study drug and are not serious adverse events, regardless of whether the event is associated with the same organ or system. 61
8.4 Adverse Events Severity Assessment 61
8.5 Causal relationship between adverse events and investigational drug 61
8.6 Recording of Adverse Events 62
8.6.1 Timely collection of adverse events 62
8.6.2 Follow-up of Adverse Events 63
8.6.3 Contents of adverse event records 63
8.7 Expedited reporting of SAEs and pregnancies 64
9 Data analysis/statistical methods 65
9.1 Statistical Hypotheses 65
9.2 Estimation of Sample Size 65
9.3 Statistical Analysis Population 65
9.4 Statistical Analysis 66
9.4.1 General Methods 66
9.4.2 Efficacy Analysis 66
9.4.3 Safety Analysis 66
9.4.4 Immunogenicity 67
9.4.5 Analysis of kinetic and pharmacodynamic parameters 67
9.4.6 Exploratory Analysis 67
9.5 Multiplicity Comparison 67
10 Quality assurance and quality control 67
11 Data management and storage 68
12 Ethics 68
12.1 Ethics Committee 68
12.2 Ethical Conduct in the Study 69
12.3 Patient Informed Consent 69
12.4 Protocol Violations 69
13 Publication policy 70
14 Protocol Revision History 71
15 REFERENCES 72

List of Tables

Table 1 Summary of Adverse Events in I8P-MC-OXAA Dose Escalation Patients ... 29
Table 2 Summary of Adverse Events Occurring in Patients with Multiple Dose Escalation in I8P-MC-OXAB Study ... 31
Table 3 Routine laboratory safety assessments ... 51

List of Figures

Fig. 1. I8P-MC-OXAB study: Change from baseline in fasting plasma glucose ... 32
Fig. 2. I8P-MC-OXAB Study: Change from Baseline in Body Weight ... 33

List of Abbreviations

Abbreviation	Full name
ADA	Anti-drug antibody
AE	Adverse Event
AESI	Adverse Event of Special Interest
ALB	Albumin
ALP	Alkaline phosphatase
ALT	Alanine transaminase
APTT	Activated partial thromboplastin time
AR	Accumulation Ratio
AST	Aspartate amino transferase
AUC	Area Under Curve
CK	Creatine Kinase
CL	Clearance
Cmax	Maximum concentration
Cr	Creatinine
DBIL	Direct bilirubin
EC	Ethics Committee
ECRF	Electronic case report form
FPG	Fasting plasma Glucose
FT3	Free Triiodothyronine
FT4	Free Thyroxine
GCGR	Glucagon receptor
GCP	Good clinical practice
GGT	Gamma-glutamyltransferase
GLP-1	Glucagon-like peptide-1
GLP-1R	Glucagon-like peptide-1 receptor
GMP	Good manufacturing practice
HbA1c	Hemoglobin A1c
HCT	Hematocrit
HCV	Hepatitis C virus
HDL-C	High density lipoprotein cholesterol
HGB	Hemoglobin
HIV	Human Immunodeficiency Virus
HOMA-Beta	Homeostasis model assessment-Beta
HOMA-IR	Homeostasis model assessment-insulin resistance
HR	Heart Rate
ICF	Informed consent form
ICH	International Conference on Harmonization
IDF	International Diabetes Federation
INR	International normalized ratio
LDH	Lactate dehydrogenase
LDL-C	Low density lipoprotein cholesterol
MedDRA	Medical Dictionary for Regulatory Activities
MTD	Maximum tolerated dose
NAb	Neutralizing Antibody
NIA	National Institute on Aging
OXM	Oxyntomodulin
PLT	Platelet

Abbreviation	Full name
PT	Prothrombin time
RBC	Red Blood Cell
SAE	Serious Adverse Event
SOPs	Standard Operation Procedure
T2DM	Type 2 diabetes mellitus
TBIL	Total bilirubin
TC	Total cholesterol
TEAEs	Treatment Emergent Adverse Event
TG	Triglyceride
TIA	Transient ischemic attack
TSH	Thyroid Stimulating Hormone
UA	Uric Acid
Vd	Volume of distribution
WBC	White blood cell
WT	Wild type

1 Study background

1.1 Background

IBI362 is a long-acting synthetic peptide similar to mammalian gastrin (OXM) that utilizes fatty acyl side chains to prolong the duration of action, allowing once-weekly dosing. When administered exogenously, OXM can improve glucose tolerance and lead to weight loss [1]. In humans, this hormone is thought to exert its biological effects by activating glucagon-like peptide-1 receptor (GLP-1R) and glucagon receptor (GCGR) [2]. As an OXM analog, the effects of IBI362 are thought to be mediated through the binding and activation of GLP-1R and GCGR. In addition to the effects of GLP-1R agonists on promoting insulin secretion and lowering blood glucose, IBI362 has now been found to exert a weight-loss effect possibly through its effect on GCGR.

The results of single and multiple dose escalation trials of IBI362 showed that it had good safety and tolerability, and also found that it could significantly reduce body weight in addition to hypoglycemic effect. The sponsor plans to develop IBI362 as glucose-reducing and weight-reducing medications for patients with type 2 diabetes.

1.1.1 GLP-1R/GCGR dual agonist

Glucagon-like peptide-1 (GLP-1) is a peptide hormone secreted by the intestine, which has multiple mechanisms designed to reduce blood glucose and weight, including glucose-dependent increased insulin secretion, inhibition of glucagon secretion, delay of gastric emptying, and inhibition of central appetite.

Glucagon is a hormone secreted by pancreatic alpha cells and consists of a single chain polypeptide of 29 amino acids in length. Glucagon exerts its physiological effects by specifically binding to the glucagon receptor (GCGR) on the surface of kidney, activating intracellular adenylate cyclase, and increasing intracellular cAMP levels. Glucagon is a hormone that promotes catabolism, and short-term injection of glucagon can promote glycogenolysis and gluconeogenesis, so that the blood glucose increased. However, it has been found that long-term activation of GCGR by glucagon injection can reduce appetite, stimulate fatty acid metabolism, and significantly increase energy expenditure in adipose tissue [3].

OXM is a peptide hormone secreted by human intestinal L-cells following nutrient ingestion. While OXM has low affinity compared to GLP-1 and glucagon, OXM is reported to be a dual agonist in cell lines expressing human GLP-1R and GCGR-mediated cAMP accumulation [4-9]. OXM acts as a dual agonist of glucagon-like peptide-1 receptor (GLP-1R) and glucagon receptor (GCGR), which exert the effects as the combination of GLP-1 and glucagon and may be more effective than GLP-1R agonists in the treatment of obesity. Concomitant injection of OXM in humans significantly reduce body weight and appetite and increase energy expenditure.

1.1.2 Type 2 diabetes mellitus

Type 2 diabetes mellitus (T2DM) is a disease dominated by disorders of blood glucose metabolism, which is characterized by chronic elevated blood glucose resulting from insulin resistance and progressive decline in pancreatic β-cell function. Poorly controlled hyperglycemia can lead to irreversible microvascular and macrovascular complications such as reduced visual acuity, blindness, renal insufficiency, peripheral neuropathy, myocardial infarction, stroke, and amputation. In 2011, the International Diabetes Federation (IDF) announced that the number of people with diabetes worldwide has reached 370 million, 80% of whom are in developing countries and are expected to have nearly 5.5 million in 2030 [10]. From 2007 to 2008, the Chinese Diabetes Society carried out an epidemiological survey of diabetes in 14 provinces and cities across the country using the current diagnostic criteria for diabetes in China, and the results showed that the prevalence of diabetes in the population over 20 years of age in China was 9.7%, and the total number of adult diabetic patients reached 92.4 million [11]. In 2010, another epidemiological survey study using the diagnostic criteria of American Diabetes Association was carried out nationwide, that is, combined blood glucose and glycosylated hemoglobin for the diagnosis of diabetes. The results showed that the prevalence rate of diabetes in Chinese adults was 11.6%. On average, 1 in 10 adults has diabetes, of which type 2 diabetes accounts for about 90% of the total number of diabetic patients, and its cases are still increasing [12]. Diabetes mellitus has a high incidence, occult and serious complications, which seriously threatens human health.

At present, it is believed that the pathophysiology and pathogenesis of type 2 diabetes are mainly because of decreased pancreatic β cell function and insulin resistance. With the progression of the disease, the function of β cells decreases year by year until failure. Traditional oral hypoglycemic agents cannot fundamentally solve the problem. Since insulin injection can mitigate the stress of pancreatic β cells to a certain extent instead of directly protecting β cells, it cannot reverse the process. In addition, the weight gain, hypoglycemia (such as insulin, sulfonylureas) and other adverse reactions caused by some hypoglycemic agents are also the challenges brought about by current hypoglycemic therapy.

1.1.3 Type 2 diabetes mellitus complicated with obesity

With lifestyle changes and the acceleration of the aging, the prevalence of type 2 diabetes and obesity is rapidly increasing, and it has become a global public health issue. Data from the 2010 Chinese Diabetes Epidemiological Survey [using glycosylated hemoglobin A1c (HbA1c) ≥ 6.5% as one of the diagnostic criteria] showed that the prevalence of diabetes in Chinese adults was as high as 11. 6%, and the number of diabetic patients ranks first worldwide [13]. Obesity is closely related to T2DM. The prevalence of diabetes in overweight and obese people in China is 12.8% and 18.5%, respectively [14]; while in diabetic patients, the proportion of overweight is 41% and the proportion of obesity is 24.

3%, abdominal obesity [waist circumference ≥ 90 cm (male) or ≥ 85 cm (female)] patients up to 45. 4% [15]. Although the occurrence of obesity is low compared with Caucasians, body fat tends to accumulate in the abdominal cavity which is more likely to form abdominal obesity [16].

Weight gain is an independent risk factor for the development of T2DM. Increased body weight or waist circumference can aggravate insulin resistance, increase the risk of T2DM, and the difficulty of glycemic control. Compared with patients with obesity alone, it is more difficult for T2DM patients with obesity to lose weight and maintain weight. First, insulin levels are significantly higher in obese patients, while insulin has the effects of inhibiting lipolysis and promoting fat synthesis. Second, obesity itself synergizes with other metabolic abnormalities present in diabetic patients to aggravate insulin resistance in T2DM, while increased visceral fat may be the main cause of insulin resistance in obese patients. Weight loss can improve insulin resistance, reduce blood glucose and improve the risk factors of cardiovascular disease. Overweight and obese T2DM patients lose 3% ~ 5% of body weight, which can produce clinically significant health benefits such as significantly reduced blood glucose, HbA1c, blood pressure and triglyceride (TG), and improve the quality of life. In a certain range, the more weight loss, the benefit [17].

1.2 Study Rationale

Glucagon-like peptide-1 receptor (GLP-1R) agonists promote glucose-dependent insulin secretion by activating peripheral GLP-1 receptors, lowering blood glucose while also decreasing gastric emptying and reducing food intake leading to weight loss. Glucagon is a catabolic hormone that promotes glycogenolysis and gluconeogenesis, resulting in a significant increase in blood glucose.

Oxyntomodulin acts as an endogenous gut-derived peptide hormone that combines the anorectic and glucose-lowering effects of GLP-1R agonists with the GCGR-stimulated increase in energy expenditure [18] [19]. GLP-1R knockout mice (GLP-1R −/−) were found to have a decrease in body weight after slow infusion of OXM, but the decrease was smaller compared with wild-type (WT) mice. This suggests that the weight-loss effect of OXM requires the simultaneous activation of two receptors, GLP-1R and GCGR [27]. Preclinical data from rodents suggest that GLP-1R/GCGR agonists are more effective in weight loss than GLP-1R agonists. Similarly, Lao et al reported that their dual GLP-1R/GCGR agonists showed higher weight loss effects in diet-induced obese rhesus monkeys [28][(29]. Preclinical data with IBI362 have also shown significant weight loss effects (decreased appetite, weight loss) in diet-induced obese mice.

Studies have shown that long-term administration of OXM has a more advantageous weight loss effect than GLP-1R agonists, and the hypoglycemic effect is equivalent [20]. This may be because OXM decreases body weight as well as glucose-dependently increases insulin secretion [21–24]. Activation of GCGR leads to increased blood glucose

levels, but this effect is antagonized by GLP-1R agonists. Basic studies have shown that rapid injection of OXM improves glucose tolerance in mice [22 – 23]. Although glucagon is thought to increase hepatic glucose output and increase blood glucose levels, recent studies have shown that intrahypothalamic glucagon inhibits hepatic glucose production, while it has been shown by pancreatic clamp testing to inhibit hepatic glucose production by stimulating the liver with circulating glucagon. Therefore, activation of GCGR in the central nervous system may improve its systemic glucose metabolism in animal tests after OXM treatment (26). The hypoglycemic effect of OXM may act mainly by lowering body weight, promoting insulin secretion, and inhibiting hepatic glucose production by activating GCGR in the central nervous system [21 – 26].

The study drug IBI362 is an OXM analog (OXM3). It is thought that endogenous OXM activates both GLP-1R and GCGR, which play an important role in regulating blood glucose and reducing body weight. Endogenous OXM has been found to excite GLP-1R less than GCGR during ERK1/2 phosphorylation associated with the cAMP signaling pathway, but the proportion of GLP-1R and GCGR is similar during cAMP-mediated Ca^{2+} influx [30]. [redacted]

This study is planned to assess the efficacy, safety and tolerability of IBI362 in patients with type 2 diabetes who have poor glycemic control with lifestyle intervention or metformin.

[redacted]

[redacted]

1.3 Potential Risks and Benefits of Treatment Options

1.3.1 Potential risks of IBI362 based on preclinical safety studies

[redacted]

[redacted]

[redacted]

1.3.2 Potential risks of IBI362 based on clinical safety studies

[redacted]

[redacted]

[redacted]

System [redacted]	[redacted]							[redacted]
	[redacted]	[redacted]	[redacted]	[redacted]	[redacted]	[redacted]	[redacted]	
[redacted]								
[redacted]					[redacted]	[redacted]	[redacted]	[redacted]
[redacted]						[redacted]	[redacted]	[redacted]
[redacted]						[redacted]		[redacted]
[redacted]					[redacted]			
[redacted]								
[redacted]		[redacted]			[redacted]	[redacted]	[redacted]	[redacted]
[redacted]								
[redacted]							[redacted]	[redacted]
[redacted]								
[redacted]	[redacted]							[redacted]
[redacted]								
[redacted]							[redacted]	[redacted]
[redacted]								
[redacted]					[redacted]			[redacted]
[redacted]	[redacted]							[redacted]

[redacted]

[redacted]

[redacted]

[redacted]

[redacted]

[redacted]	[redacted]					[redacted]
	[redacted]	[redacted]	[redacted]	[redacted]	[redacted]	
[redacted]						
[redacted]	[redacted]		[redacted]	[redacted]	[redacted]	[redacted]
[redacted]						
[redacted]				[redacted]	[redacted]	[redacted]
[redacted]	[redacted]	[redacted]	[redacted]	[redacted]		[redacted]
[redacted]	[redacted]		[redacted]		[redacted]	[redacted]
[redacted]			[redacted]	[redacted]		[redacted]
[redacted]			[redacted]	[redacted]		[redacted]
[redacted]			[redacted]			[redacted]
[redacted]					[redacted]	[redacted]
[redacted]	[redacted]					[redacted]
[redacted]					[redacted]	[redacted]
[redacted]				[redacted]		[redacted]
[redacted]				[redacted]		[redacted]
[redacted]						
[redacted]			[redacted]	[redacted]	[redacted]	[redacted]
[redacted]				[redacted]		[redacted]
[redacted]						
[redacted]	[redacted]				[redacted]	[redacted]

[redacted]	[redacted]					[redacted]
	[redacted]	[redacted]	[redacted]	[redacted]	[redacted]	
					[redacted]	
[redacted]				[redacted]		[redacted]
[redacted]						
[redacted]				[redacted]		[redacted]

[redacted]

[redacted]

[redacted]

[redacted]

[redacted]

[redacted]

2 Study objectives and endpoints

2.1 Study objectives

2.1.1 Primary objective

- To investigate the safety and tolerability of multiple subcutaneous injections of IBI362 in patients with type 2 diabetes inadequately controlled by lifestyle intervention or metformin, and to determine the safe dose range.

2.1.2 Secondary objectives

- To investigate the pharmacokinetic/ pharmacodynamics (PK/PD) parameters of multiple subcutaneous injections of IBI362 in patients with type 2 diabetes inadequately controlled by lifestyle intervention or metformin.

2.2 Study Endpoints

2.2.1 Primary endpoint

- **Safety and tolerability:** During dose escalation, the incidence and severity of adverse events at each dose level, including chief complaint, physical examination, laboratory tests (hematology, blood chemistry, lipid panel, coagulation function, urinalysis, myocardial enzymes, serum amylase and/or lipase, thyroid function, calcitonin, etc.), vital signs (pulse, respiration, blood pressure, body temperature), 12-lead ECG and other examinations; treatment compliance; and record the name, clinical characteristics, severity, start and stop time, supportive care and outcomes of adverse events, correlation with the study drugs;

- **Maximum tolerated dose:** If the dose exploration is stopped when the criteria for stopping dose escalation are met at a certain dose level, the previous dose is the maximum tolerated dose. If the criteria are still not met when the maximum exploratory dose is reached, the maximum tolerated dose is greater than or equal to the maximum escalation dose.

2.2.2 Secondary endpoints

- To evaluate the pharmacokinetic parameters of IBI362 in patients with type 2 diabetes, including but not limited to: time to maximum concentration (T_{max}), maximum concentration (C_{max}), area under the curve (AUC), volume of distribution (Vd), half-life ($T_{1/2}$), clearance (CL), accumulation ratio (AR), etc.;
- To assess the pharmacodynamics parameters of IBI362 after multiple doses: changes of fasting blood glucose, glucagon, insulin, fasting C-peptide, hemoglobin A1c (HbA1c), oxytomodulin (OXM) and GLP-1 after administration;
- Changes of fasting blood glucose, insulin and glucagon at steady state in each dose level during dose escalation;
- Changes from baseline in each parameter of MTT test after multiple dose administration;
- Changes of self-measure blood glucose after multiple doses;
- The incidence of anti-IBI362 antibody (ADA) and neutralizing antibody (NAb) in serum before and after administration

2.2.3 Exploratory Endpoints

- To evaluate the effect of IBI362 on weight loss (changes in fasting body weight, waist circumference and BMI);
- To assess the improvement of the pancreatic function: changes in homeostatic model assessment (HOMA) of β-cell function and insulin resistance (IR).

3 Overall Study Design

3.1 Design Rationale

This study will be the first to evaluate the safety, tolerability and PK/PD of IBI362 administered as multiple injections in Chinese patients with type 2 diabetes. A multicenter, randomized double-blind, placebo-controlled trial design will be used, while dulaglutide is set as an open-label active control.

3.1.1 Dose Selection Principle

IBI362 is proposed to be studied for the tolerability of 3 dosing regimens (1 mg QW4 + 2 mg QW4 + 3 mg QW4; 1.5 mg QW4 + 3 mg QW4 + 4.5 mg QW4; 2 mg QW4 + 4 mg QW4 + 6 mg QW4) in Chinese patients. The dose selection and interval design for

this tolerability study will be primarily based on the following considerations:

1）Safe Induction Dose

[redacted]. The proposed induction doses of 1 mg and 1.5 mg QW4 are the validated safe dose range. The 2-mg serial dosing regimen will be initiated 4 weeks after the start of the 1.5-mg serial dosing regimen with acceptable safety.

2）Inducible Tolerance Potential

[redacted]

3) Mature safety monitoring methods and slow climbing speed

The major adverse effects of OXM-3 targets arise from gastrointestinal reactions and are easily monitored. Both 1.0 mg and 1.5 mg are verified safe doses. The increase is 33.3% in the 2.0-mg dose group compared with previous dose group, and the rate of increase is slow.

4) Potential for better therapeutic benefit

[redacted]

5) [redacted]

[redacted]

3.2 Phase 1b/2 Study Design

3.2.1 Overall Study Design

The study will be planned to include 42 patients with type 2 diabetes who failed to control HbA1c after at least 2 months of lifestyle intervention or stable dose of metformin (≥1000 mg/day or maximum tolerated dose). The study will be divided into three cohorts, which will be Cohort 1 (n = 14), Cohort 2 (n = 14) and Cohort 3 (n = 14). Patients in each cohort will be randomized 8:4:2 to IBI362 group (n = 8), placebo group (n = 4), or dulaglutide 1.5 mg group (n = 2). The active control, dulaglutide, will be administered as 1.5 mg once-weekly for 12 weeks in Cohorts 1, 2 and 3, and the dosing schedules for IBI362 and placebo are described below:

Cohort 1: Patients randomized to IBI362 or placebo initiate treatment with 1.0 mg once-weekly with dose escalations every 4 weeks by 1 mg until the maintenance dose (3.0 mg) will be achieved.

Cohort 2: Patients randomized to IBI362 or placebo initiate treatment with 1.5 mg once-weekly with dose escalations every 4 weeks by 1.5 mg until the maintenance dose (4.5 mg) will be achieved.

If the patient failed tolerate 3.0 mg or 4.5 mg with the met discontinuation criteria, the patient needs to explore the next dose according to Cohort 2 (backup 1) or Cohort 2 (backup 2).

Cohort 2 (backup 1): With the tolerated dose of 3.0 mg, the dose should be reduced to 2.25 mg after 1-week break and treat with it for 2 weeks. After that, if it is well-tolerated, the dose will be increased to 3.0 mg and treat for 4 weeks.

Cohort 2 (backup 2): If the dose of 4.5 mg is intolerable to the patient, the dose should be reduced to 3.75 mg after 1-week break and administered continuously for 2 weeks till the end of the trial.

Cohort 3: Dosing should not be started until the patients in Cohort 2 complete the 4-week tolerability evaluation of 1.5 mg. If 1.5 mg is not well-tolerated, 2.0 mg and higher doses in Cohort 3 will not be explored. Patients randomized to IBI362 or placebo initiate treatment with 2.0 mg once-weekly with dose escalations every 4 weeks by 2.0 mg until the maintenance dose (6.0 mg) will be achieved.

The study will consist of a 3-week screening period, a 12-week double-blind treatment period, and an 8-week follow-up period.

3.2.2 Measures to control bias

To avoid possible bias, a double-blind placebo-controlled design is used in the study. At the same time, objective measurements are used for safety and efficacy endpoints which is hardly lead to bias.

3.2.3 Discontinuation Criteria

If any of the following criteria are met, the investigator and the sponsor should immediately initiate a discussion procedure to decide whether to terminate the dose escalation in each cohort.

- Occurrence of at least one serious treatment-emergent adverse event related to IBI362 (except for expected adverse events related to the pharmacological properties of IBI362, such as hypoglycemia, etc.).
- No less than 4 patients experience symptomatic hypoglycemic events (plasma glucose levels < 3.0 mmol/L) at a specific dose level, and those events are considered to be related to IBI362.
- No less than 2 patients receiving IBI362 experience persistent (more than one week) symptoms of acute pancreatitis.
- No less than 2 patients experience severe IBI362-related, non-serious adverse events at the same dose, regardless of whether the event is associated with the same organ or system.

4 Study Population

Deviations from eligibility criteria are not allowed because they may affect the scientific integrity of the study, regulatory acceptability, and/or patient safety. Therefore, patients must meet protocol-specified criteria.

4.1 Inclusion criteria

Patients will be included in the study if they met all of the following criteria:

1. A diagnosis of type 2 diabetes, as defined by WHO (1999) for at least 6 months.
2. Male or female, aged 18 to 75 years at signing informed consent.
3. Blood glucose will be inadequately controlled by lifestyle intervention alone or with stable dose of metformin (≥ 1000 mg/day or maximum tolerated dose) within 2 months prior to screening.
4. 7.5% ≤HbA1c≤11.0% by local laboratory at screening.
5. 20 ≤ BMI ≤ 35 kg/m 2 (BMI = weight (kg)/height (m^2)) .
6. Maintain a stable diet and regular exercise lifestyle during the study.
7. Willing to sign the Informed Consent Form (ICF) and able to participate in the trial and comply with all trial requirements.

4.2 Exclusion Criteria

Patients will be excluded from the study if they met any of the following criteria:

1. Type 1 diabetes, gestational diabetes, or other types of diabetes.
2. Ketoacidosis or lactic acidosis within 6 months prior to screening.
3. History of severe hypoglycemic episodes within 6 months prior to screening, defined as symptoms of neuroglycopenia that require supportive care from others, or complete ignorance of hypoglycemia or lack of awareness of hypoglycemic symptoms. Patients who are unable to communicate and understand the symptoms of hypoglycemia and appropriate treatment should also be excluded from the trial at the discretion of the investigator .
4. Acute myocardial infarction, unstable angina pectoris, coronary artery bypass grafting, coronary intervention (except diagnostic angiography), transient ischemic attack (TIA), cerebrovascular accident, acute and chronic heart failure within 6 months before screening.
5. Abnormal 12-lead ECG (e.g., QTcF > 450 ms, PR interval < 120 ms, or PR interval > 220 ms, second-degree and third-degree atrioventricular block, ventricular conduction delay, i.e., QRS > 120 ms, right bundle branch block, left bundle branch block, Wolff-Parkinson-White syndrome) at screening that increase the risk of the patient or may cause confusion in the analysis of ECG data (QT); or taking any drug that may affect the QT interval at the discretion of the investigator, e.g., antiarrhythmic drugs class IA and III, cisapride, macrolide antibiotics and psychotropic drugs (phenothiazines [pyridazine, chlorpromazine, pyridazine], butyrophenones [droperidol, haloperidol] and loperamide).
6. Previous diagnosis of long QT syndrome.
7. Uncontrolled blood pressure at screening with systolic blood pressure > 140 mmHg or < 90 mmHg and diastolic blood pressure > 90 mmHg or < 50 mmHg.
8. Heart rate < 50 bpm or > 90 bpm at screening.
9. Active or untreated malignancy within 5 years prior to screening, or in clinical remission of malignancy (except for patients with basal and squamous cell carcinoma of the skin, carcinoma in situ of the cervix, and papillary thyroid carcinoma without postoperative recurrence).
10. Previous history of acute or chronic pancreatitis, or serum lipase/amylase > 2 × upper limit of normal at screening, or fasting triglycerides > 5.65 mmol/L (500 mg/dl). If the patient is on lipid-modifying therapy, the dose must be stable for 30 days prior to screening.
11. Clinically symptomatic liver disease, acute or chronic hepatitis, or transaminases (ALT and AST) and alkaline phosphatase (ALP) > 2 × upper limit of normal and total bilirubin > upper limit of normal at screening.

12. Calcitonin ≥ 15 ng/L at screening.
13. EGFR < 60 mL/min/1.73 m2 at screening, estimated using the modified MDRD formula: eGFR = 175 × [(serum creatinine (μmol/L)/88.4)] -1.234 × [age (years)] -0.179 × 0.79 (female) or × 1 (male).
14. History or presence of psychiatric disorder at screening that would make participation in this study inappropriate at the discretion of the investigator.
15. Have Diagnosed of gastroparesis or received any form of obesity surgical procedure, or had abnormal gastric emptying considered clinically significant by the investigator.
16. Known history of regular drug abuse.
17. Positive human immunodeficiency virus (HIV) antibody and/or HIV infection at screening, or positive syphilis antibody.
18. History of hepatitis B and/or positive hepatitis B surface antigen at screening or positive hepatitis C (HCV) antibody at screening.
19. Previous diagnosis of Gilbert's syndrome.
20. International normalized ratio (INR) of prothrombin time greater than the upper limit of normal at screening.
21. History of medullary thyroid C-cell carcinoma, MEN (multiple endocrine neoplasia) syndrome 2A or 2B, or relevant family history.
22. Diagnosed with autonomic neuropathy, manifested as: urinary retention, resting tachycardia, orthostatic hypotension, and diabetic diarrhea.
23. Significant weight change with a percentage change > 5% within 3 months prior to screening.
24. Donation of ≥ 400 mL of blood or excessive blood loss or bone marrow transplantation within 3 months before screening, or presence of hemoglobinopathy, hemolytic anemia, sickle cell anemia, or hemoglobin < 110 g/L (men) or < 100 g/L (women).
25. Hyperthyroidism or hypothyroidism confirmed by clinical assessment and/or TSH abnormality that, in the opinion of the investigator, may increase the risk of the patient.
26. Use of antidiabetic drugs other than metformin within 2 months prior to screening.
27. Use or planned to use weight-lowering medications such as Liraglutide, Orlistat, Sibutramine HCl, Phenylpropanolamine, Chlorphenindole, Phenylbutamine, Chlorocarbazone HCl, Fintramine, Fintramine/Topyrate, Bupropion, Naltrexone/Bupropion within 3 months prior to screening during the trial.
28. Chronic use of glucocorticoids (cumulative or continuous use > 2 weeks) within 1 year before screening, or use of glucocorticoids (excluding topical, intraocular, intranasal, intra-articular, inhalation) within 4 weeks before screening.

29. Ongoing central nervous stimulant use (e.g., methylphenidate hydrochloride) at screening, with the exception of caffeinated beverages.
30. Known history of hypersensitivity to study drugs or drug components.
31. Participation in a clinical trial of any drug or medical device within 3 months prior to screening (randomized and received medication).
32. Female with childbearing potential, except those who are sterilized or amenorrheic, who are unwilling to inform their sexual partners of their participation in this clinical study and do not take effective contraceptive measures during the study. Male who are unwilling to inform their female sexual partners of the participation in the clinical study and unwilling to take effective contraceptive measures during the study.
33. Pregnant or lactating women or women who are preparing for pregnancy or breastfeeding during the study period
34. Any clinically significant abnormal laboratory value that may interfere with the interpretation of efficacy and safety data at the discretion of the investigator in this study.
35. Patients with any other factors that may affect the their cooperation, efficacy, or safety evaluation at the discretion of the investigator, such as the presence of psychiatric disorders.
36. Average weekly alcohol intake of more than 21 units for men and 14 units for women, or unwillingness to stop drinking 24 hours before the day of medication and throughout the study (1 unit = 360 ml of beer, or 150 ml of red wine, or 45 ml of distilled spirits/liquor).

4.3 Restrictions during the study

- Patients will be abstinent from alcohol throughout the study.
- Smoking will be restricted (< 10 cigarettes/day) during the study and will be prohibited 1 hour before vital signs and ECG assessments.
- The patient donated blood during the study and 8 days after the end of medication;
- Males or females of childbearing potential are required to use 8 g of contraception during the study.

4.4 Patient Discontinuation/Withdrawal

4.4.1 Treatment discontinuation

1) Liver enzyme and other laboratory tests

In any case, discontinuation of study drug should be considered after joint discussion of investigator and the sponsor;
When a patient has baseline ALT/AST < 1.5 ×ULN:

- ALT or AST $\geq$ 5 × ULN

- ALT or AST ≥ 4x ULN for more than 2 weeks
- ALT or AST ≥ 3x ULN and TBL > 2x ULN or international normalized ratio (INR) ≥ 1.5
- ALT or AST ≥ 3x ULN with symptoms of fatigue, nausea, vomiting, right upper quadrant pain/tenderness, fever, rash and/or eosinophilia (> 5%)
- ALP > 3x ULN
- ALP ≥ 2.5 x ULN and TBL > 2 x ULN
- ALP ≥ 2.5x ULN with symptoms of fatigue, nausea, vomiting, right upper quadrant pain/tenderness, fever, rash and/or eosinophilia (> 5%)

When a patient has baseline ALT/AST ≥ 1.5 x and < 2 x ULN:

- ALT or AST ≥ 4 x baseline
- ALT or AST ≥ 3 x baseline for more than 2 weeks
- ALT or AST ≥ 2 x baseline and TBL > 2 x ULN or international normalized ratio (INR) ≥ 1.5
- ALT or AST ≥ 2 x baseline with symptoms of fatigue, nausea, vomiting, right upper quadrant pain/tenderness, fever, rash, and/or eosinophilia (> 5%)

Note: If the patient is found to have ALT/AST at screening, the baseline data will be collected at least 2 weeks apart.

- CK elevation > 5x ULN
- Lipase and/or amylase ≥ 3 x ULN, the possibility of pancreatitis should be considered by the investigator (Table 5)

2) Hyperglycemia/hypoglycemia:
   The study drug should be discontinued due to deterioration in blood glucose control during the trial, fasting blood glucose > 13.3 mmol/L with other excluded causes (e.g., increased blood glucose due to concurrent disease);

The patient had a severe hypoglycemic event or persistent hypoglycemia (see section 8.3.4 for details);

3) The patient was inadvertently withdrawn from the study if the sponsor or investigator determined that the patient did not meet the enrollment criteria.

4.4.2 Withdrawal

1) Participation in other clinical trials during the trial is defined as signing the informed consent form for other trials;

2) The investigator may terminate or withdraw a subject from the study because of AEs. (Considering that gastrointestinal adverse reactions may be related to the efficacy, it is recommended that the investigator may consider to adjust the titration rate at first. Only if the severe adverse events continues after adjustment, the investigator will terminate the patient because of intolerance);
3) Patients with poor compliance who do not receive medication or test before completing the whole trial and cannot insist on completing the trial as planned, including patients who cannot do a good job in diet control, do not take medicine as required, or have other conditions that may affect the efficacy evaluation;
4) Withdrawal of consent by the patient;
5) Patients with blindness due to various reasons;
6) Female patient becomes pregnant;
7) The investigator considers it inappropriate to continue to participate in the trial.

4.4.3 Withdrawal procedures

If the decision is made to permanently discontinue study drug, the patient will be withdrawn from the study and an End of Treatment Visit should be performed. Reasonable efforts should be made to persuade the patient to complete the follow-up visit. These visits are described in Section 6. The date of last dose for study drugs and the reason for withdrawal will be recorded in the eCRF.

4.5 Patients Withdrawal

If the patient discontinues study treatment and actively withdraws consent for collection of follow-up data, then no additional data collection should occur; however, patients may elect to withdraw consent for study treatment but continue to accept follow-up to assess safety/efficacy.

5. Study Drug and Other Therapies

5.1 Treatment Allocation

5.1.1 Treatment allocation

Patients will be divided into 3 treatment cohortss: Cohort 1, Cohort 2, and Cohort 3. Patients in each cohort were randomized to IBI362: placebo: active comparator = 8:4:2.

5.1.2 Randomization and blinding

This study is a randomized, double-blind trial of IBI362 versus placebo within each dose group. At each dose, each patient will be randomized to receive IBI362, placebo, active control treatment in a ratio of 8:4: 2. If a patient is assigned to receive

IBI362 or placebo, the patient, the investigator, and all personnel involved in the clinical evaluation will be blinded to the treatment from the randomization to database locked, as follows:

Different drug numbers corresponded to random numbers after randomization in the IWRS, and the unblinding information remained unknown to patients and investigators throughout the trial. The randomization table will be kept by a designated person.

The study drug and placebo will be packaged, labeled, administered at the same time, and will have the appearance, taste, and smell exactly the same to hide the real situation of the treatment drug.

If necessary for the safety of the subject, emergency unblinding must be performed using the IWRS.

If a patient is randomized to receive active control, the patient will not be blinded.

Sponsor will not be blinded during this study.

5.2 Study Drug

5.2.1 Physical and Chemical Characteristics of Drug Substance

Molecular weight:	4560.32 Dalton
Description:	White to off-white powder
PI:	5.2
Solubility:	Vehicle: 20 mM Tris buffer pH 8.0 and 150 mM sodium chloride.<br>Solubility: Not less than 10 mg/mL at 25℃.<br>Solubility Description: Sparingly or slightly soluble.

5.2.2 Study Drug Strength and Manufacturer

IBI362 is formulated as IBI362 for injection in a strength of 2 mg per vial and consists of 2 mg IBI362 and the inactive ingredients trihydroxymethylaminomethane, mannitol, and sucrose.

The strength of the formulation used in this study is 2 mg/vial; Manufacturer: Supplied by the Sponsor.

Placebo is a lyophilized powder matching IBI362 Manufacturer: Supplied by the Sponsor.

Dulaglutide is supplied as 1.5 mg/0.5 ml single-dose pen manufactured by Vetter Pharma-Fertigung GmbH & Co. KG.

5.2.3 Storage

The drug product will be stored in sealed for 2-8°Crefrigerator.

5.2.4 Mode of administration

IBI362 will be administered by subcutaneous injection once-weekly for 12 consecutive weeks. Each dose must be prepared and injected by the designated nurse .

5.3 Concomitant Therapy

5.3.1 Prohibited Drugs

The following medications and measures are prohibited:

1. Any drug with hypoglycemic effect other than metformin and study drugs is prohibited;
2. Growth hormone and its analogues;
3. Any systemic use of corticosteroids (including intravenous, oral administration) for ≥ 7 days. Corticosteroids: mainly glucocorticoids, including short-acting: hydrocortisone and cortisone; intermediate-acting: prednisone, prednisolone, methylprednisone and triamcinolone; long-acting: dexamethasone and betamethasone;
4. Drugs to control body weight, such as pramlintide, sibutramine hydrochloride, orlistat, zonisamide, topiramate, phenylbutamine, chlorkasaline hydrochloride, finamine, finamine, topiramate, bupropion, naltrexone/bupropion, and some health care products with indications of "weight loss";
5. Drugs that may affect body weight: tricyclic antidepressants, psychiatric drugs, and tranquilizers, such as promizine, amitriptyline, mirtazapine, paroxetine, phenelzine, chlorpromazine, thioridazine, chlorzapine, olanzapine, valproic acid, valproic acid derivatives, lithium;
6. Any drug or herbal medicine known to have common toxic effects on major organs, or any drug that may interfere with the interpretation of efficacy and safety data.

5.4 Administration During Pregnancy, Childbearing Age, or Lactation

5.4.1 Pregnancy

Women who are pregnant cannot be enrolled in this study. Subjects with childbearing potential must use the highly effective contraception

5.4.2 Subjects with Childbearing Potential

For women with childbearing potential who are sexually active with a nonsterilized male partner and nonsterilized male patients who are sexually active with a women with childbearing potential, the patient and their sexual partners must use at least 1 acceptable effective method of contraception within the 8 weeks of the last administration and should discuss the discontinuation of contraception with a responsible physician after that .

Women who have been amenorrheic for 12 months without an alternative medical cause are considered postmenopausal. The requirements according to age are as follows:

- Women ≥ 50 years of age may be considered postmenopausal if they have been amenorrheic for 12 months or more after stopping exogenous hormone therapy and their luteinizing hormone and follicle stimulating hormone levels are in the recognized postmenopausal range. Women < 50 years of age may be considered postmenopausal if they have been amenorrheic for 12 months or more after stopping all exogenous hormone therapy, have had a radiotherapy-induced oophorectomy with amenorrhea occurring > 1 year earlier, have had chemotherapy-induced amenorrhea with > 1 year since the last amenorrhea, or have undergone surgical sterilization (oophorectomy or hysterectomy).

5.4.3 Nursing women

Breastfeeding women cannot be enrolled in this study.

5.5 Treatment Compliance

Study treatment will be administered at the study site, and treatment compliance will be measured using drug receipt and dispensing records, patient's original medical records, and eCRFs.

5.6 Drug Return and Destruction

At the end of the study, all unused study drug will be shipped back to the sponsor or its designee under the supervision of the investigator or designee for destruction according to institutional standard operating procedures. If local procedures require destruction of study drug supplies at the site, study drug supplies should be retained at the site (where local procedures permit) until the study monitor checks the accountability records to assess compliance and accuracy of site accountability records. If a site destroys study drug prior to monitor inspection, the monitor will perform an evaluation based on the records of destruction per site SOPs.

5.7 Documentation of Study Drug

The investigator is responsible for drug accountability at the study site; however, certain drug accountability responsibilities may be assigned to an appropriate pharmacist or other designee. Inventory and accountability records must be maintained and readily available for inspection by the study monitor and all applicable regulatory authorities. The investigator or designee must maintain records of:

1. Study drug delivery records at the study site.
2. Investigational product inventory at the site.
3. Documentation of study drug administration to the patient, including the number of units supplied each time.

The investigational product must be used as specified in the protocol. The investigator will also maintain records adequately documenting that the patient will be provided the study drug specified. These records should include dates, quantities, and any available batch numbers or unique code numbers assigned to the investigational product and study patients.

Completed accountability records are archived at the site. The investigator or designee is expected to collect and retain all used, unused, and partially used containers of study drug until verified by the clinical monitor (unless received the otherwise agreement from the sponsor).

5.8 Complaint handling

The sponsor collects product complaints for the study drug and drug delivery system used in the clinical study to ensure the safety of patients, monitor quality, and facilitate process and product improvements.

All product complaints associated with the packaging, labeling, and release of materials by the sponsor or designee will be reported to the sponsor. All product complaints associated with other study materials will be reported directly to the respective manufacturer.

The investigator or designee is responsible for reporting complete information on a product complaint via email or other written communication to the sponsor contact or manufacturer representative as documented in the packaging information. Any AE associated with a product complaint should be reported as described in Section 8 of this protocol.

If the investigator is requested to return the product for investigation, return a copy of the product complaint form with the product.

6 STUDY ASSESSMENTS AND PROCEDURES

6.1 Inclusion Procedures

6.1.1 Inclusion of patients

The investigator will follow the following steps:

1. Obtain informed consent with the signature of the patient or the patient's legal representative before performing any study-related procedures.

2. the principal investigator or appropriately trained designee formally determines the patient's eligibility after reviewing the inclusion/exclusion criteria.

Patients who do not meet the relevant criteria for this study (screen failure) may be rescreened. If re-screening of a patient is considered, the investigator must contact the sponsor's medical monitor. Each patient may be rescreened once. At re-screening, the patient or the patient's legal representative must re-sign the informed consent form (ICF) and will be reassigned a screening number.

Procedures for Handling Incorrectly Enrolled Patients:

The inclusion criteria must be strictly adhered to. If a patient who does not meet the inclusion and exclusion criteria is found, the sponsor medical monitor and the investigator will discuss and determine whether to continue the patient in the study.

6.1.2 Screening period and Baseline Period

The following study procedures must be completed during the screening period (Day -21 to Day -2) and baseline period (D-1) to ensure that patients are eligible for the study:

- Signed informed consent
- Check inclusion/exclusion criteria
- Record demographics, past medical history and past medications
- Record vital signs, physical examination
- Height, weight, waist circumference
- 12-lead Electrocardiogram
- Hematology, blood biochemistry, blood lipid, coagulation function, urinalysis, myocardial enzyme spectrum, serum amylase and/or lipase, calcitonin
- Thyroid function
- SMG Training, Patient Diary Card and Meter Dispensing
- Glycosylated hemoglobin
- Standard meal test (MTT)
- Seven point fingertip capillary blood glucose (Within 2 Days Prior to D-1 Visit)

- Pregnancy test (women of childbearing potential)
- Infectivity indicators: HIV antibody, hepatitis B (5 items), HCV antibody, syphilis antibody
- Assessment of Adverse Events
- Concomitant medication

6.2 Double-Blind Treatment Period

The following study procedures should be completed for treatment period visits:

- Physical examination
- Record vital signs
- Weight, waist circumference
- 12-lead Electrocardiogram
- Hematology, blood biochemistry, blood lipid, coagulation function, urinalysis, myocardial enzyme spectrum, serum amylase and/or lipase, calcitonin
- Glycosylated hemoglobin
- Seven point fingertip capillary blood glucose (within 2 days prior to D29, D57, D85 visit)
- Pregnancy test (women of childbearing potential)
- Assessment of Adverse Events
- Record concomitant medication
- Study Drug Administration
- Infusion Reactions
- Immunogenicity
- PK/PD sampling
- Standard meal test (D80)

6.3 Safety Follow-up

- Vital Signs
- Follow up and record adverse events
- Follow up and record concomitant medications
- Immunogenicity

6.4 Early Withdrawal Visits

- Physical examination

- Record vital signs
- Weight, waist circumference
- 12-lead Electrocardiogram
- Hematology, blood biochemistry, blood lipid, coagulation function, urinalysis, myocardial enzyme spectrum, serum amylase and/or lipase, calcitonin
- Glycosylated hemoglobin
- Pregnancy test (women of childbearing potential)
- Assessment of Adverse Events
- Record concomitant medication
- PK/PD sampling and immunogenicity

* The end of the study will be defined as the time when the patient completed the last visit.

7 STUDY ASSESSMENTS

7.1 Safety and Tolerability Assessments Metrics

- **Safety and tolerability:** During dose escalation, the incidence and severity of various adverse events at different dose stages will be recorded. The main complaints of patients, physical examination, laboratory tests (blood routine, blood biochemistry, blood lipid, coagulation function, urine routine, myocardial enzyme spectrum, serum amylase and/or lipase, thyroid function, calcitonin, etc.), vital signs (pulse, respiration, blood pressure, body temperature), 12-lead ECG and other examinations; medication compliance analysis will be performed to determine the correlation with the study drug and record the name, clinical characteristics, severity, occurrence and end time, treatment methods and outcomes of adverse events, correlation with the study drug and other information;
- **Maximum tolerated dose:** If the dose exploration is stopped when the criteria for stopping dose escalation are met at a certain dose level, the previous dose is the maximum tolerated dose. If the criteria are still not met when the maximum exploratory dose is reached, the maximum tolerated dose is greater than or equal to the maximum escalation dose;

7.2 Safety and Other Assessments

7.2.1 Laboratory Tests

7.2.1.1 Routine laboratory safety assessments

Specific laboratory procedures/assessments are detailed as below. The total amount of blood/tissue to be collected throughout the trial (from pre-trial to post-trial visits),

including at each visit for each patient and for each specimen type, is described in the Study Procedures Manual. Refer to the Laboratory Assessments section in the Table 2.

7.2.1.2 Laboratory Safety Evaluations

Hematology, blood biochemistry, blood lipid, coagulation function, urinalysis, myocardial enzyme spectrum, thyroid function, viral serology, serum amylase and/or lipase, calcitonin, etc. For laboratory tests see Table 4.

Table 4 Routine laboratory safety assessments

Blood routine	White blood cell count (WBC), red blood cell count (RBC), platelet (PLT), hemoglobin concentration (HGB), hematocrit (HCT), differential white blood cell count (neutrophils, basophils, eosinophils, monocytes, and lymphocytes).
Blood biochemistry	Aspartate aminotransferase (AST), alanine aminotransferase (ALT), total bilirubin (TBIL), direct bilirubin (DBIL), albumin (ALB), total protein, glutamyl transpeptidase (GGT), alkaline phosphatase (ALP), lactate dehydrogenase (LDH), serum potassium, serum sodium, serum calcium, serum chloride, uric acid (UA), urea, creatinine (Cr).
Lipids	Total cholesterol (TC), triglyceride (TG), high-density lipoprotein cholesterol (HDL-C), and low-density lipoprotein cholesterol (LDL-C).
Coagulation function	Prothrombin time (PT), activated partial thromboplastin time (APTT), international normalized ratio (INR)
Urine routine	Urine pH, urine protein, urine glucose, urine red blood cells, urine white blood cells.
Cardiac enzymes	Creatine kinase (CK), creatine kinase isoenzyme (CK-MB)
Thyroid function	Free triiodothyronine (FT3), free thyroxine (FT4), thyroid-stimulating hormone (TSH).
Viral serology	HBsAg, HBsAb, HBcAb, HBeAg, HBeAb, HCV antibody, HIV antibody and syphilis antibody.
Other	Amylase, lipase, calcitonin

HBcAb: hepatitis B core antibody; HBeAb: hepatitis B E antibody; HBeAg: hepatitis B E antigen; HBsAb: hepatitis B surface antibody; HBsAg: hepatitis B surface antigen; HBV: hepatitis B virus; HCV: hepatitis C virus; HIV: human immunodeficiency virus.

7.2.2 Physical examination

7.2.2.1 Complete physical examination

The investigator or other designee will perform a complete physical examination during screening. Complete physical examination includes: general condition, respiratory, cardiovascular, abdominal, skin, head and neck (including ears, eyes, nose and throat), lymph nodes, thyroid, musculoskeletal (including spine and limbs) and neurological assessments. Clinically significant abnormal findings should be recorded as medical history. In addition, a complete physical examination will be performed as specified in

the Table 2. After signing informed consent, newly identified clinically significant abnormal findings will be recorded as AEs.

7.2.2.2 Targeted physical examination

For cycles in which a complete physical examination is not required in the trial, the investigator or qualified designee will perform a targeted physical examination in the presence of clinical findings, scheduled prior to dosing in each treatment cycle. New clinically significant abnormal findings should be recorded as AEs.

7.2.3 Vital Signs

Patients' vital signs (including temperature, pulse, respiration, and blood pressure) will be monitored, recorded, and evaluated according to the trial procedures (see Table 2 for scheduled visits). Any emerging clinically significant abnormalities will be reported as AEs.

Additional monitoring of vital sign assessments may be performed at the discretion of the investigator based on standard clinical practice or as clinically indicated. In the event of an AE/SAE, the investigator may collect additional vital sign values (if applicable) and record them in the original medical records and eCRF, along with the date and time of the measurement.

Before the measurement of blood pressure, pulse and respiratory rate, the patient should sit quietly for at least 5 minutes, and should not drink coffee or do strenuous exercise and empty the bladder within 30 minutes before blood pressure measurement. During each visit, the patient's sitting blood pressure should be measured at least twice in succession with an interval of 1 ~ 2 minutes. The investigator should record the mean value of blood pressure and the measuring time in the original medical records and eCRF. For the same patient, blood pressure should be measured in the same upper arm at each visit.

7.2.4 ECG

It is recommended that a 12-Lead ECG be obtained after the subject has rested for at least 5 minutes in the recumbent position. If not, lie as oblique as possible.

12-lead. Electrocardiogram must include the following examination indicators: heart rate (HR), QRS complex time, QT interval, corrected QT interval (QTc) and PR interval.

All ECGs should be reviewed by the principal investigator or designated study site physician, and any clinically significant abnormality should be reported as an AE. After review and signature, the original ECG tracing map shall be preserved with the original records of patients. When requested by the sponsor, a copy of the original ECG with hidden subject’s information will be sent to Innovent. Additional ECGs may be performed at the discretion of the investigator according to standard clinical practice or clinical needs and recorded in the original medical records and eCRF, with the measuring time recorded

by the investigator in the appropriate section of the original medical records.

7.2.5 Fasting Body Weight Measure

For each patient, their weight measurements should be performed in a uniform manner at each clinic visit using calibrated scales, either mechanically or electronically. Each weight measurement of a patient should be taken after emptying his/her bladder using the same scale. Patients should remove coat/ pants/ caps/ scarves/ necklaces/ belts/ shoes and other weight-increasing clothing. Single clothing (only one jacket and one underwear) is allowed.

1. Before weighing, ensure that the pointer of the scale has been zeroed;
2. Before weighing, ensure that the scale is placed on a firm and smooth surface (do not place carpets or inclined or rough surfaces);
3. Before weighing, the curtain should be drawn to isolate the weighing area and to protect the privacy of patients when changing their clothing;
4. Before weighing, ensure that the patients are not cold after undressing;
5. After urinated, the patient will be guided to the weighing area, the patient is required to take off his/her coat/coat/hat/scarf/necklace/belt and any other weight-increasing clothing, wear only clothes (only one jacket and one underwear), and take off shoes;
6. Patients will be asked to step on the scale with their feet on each side of the scale and stand still with their arms resting on both sides of their body. Their weight will be recorded in kilograms (kg).

7.2.6 Pregnancy Testing

Blood/urine pregnancy test is required for women of childbearing potential before enrollment. If the pregnancy test is positive, the patient is not eligible or must be discontinued from the study. If pregnancy is suspected during the study, a retest should be performed.

7.3 PK/PD Measurements

7.3.1 PK Blood Sampling Points

- Week 1 (intensive PK) PK sampling points: within 1 h before administration, 4 h ± 10 min, 8 h ± 20 min, 12 h ± 30 min, 24 h ± 1 h (Day 2), 48 h ± 2 h (Day 3), 72 h ± 3 h (Day 4), 96 h ± 4 h (Day 5), 120 h ± 5 h (Day 6) after administration, and within 1 h before administration on Day 8.
- Week 4: D22 for 48h ± 2h.
- Sparse sampling in Week 5: within 1 h before administration on Day 29, 4 h ±

10 min, 8 h ± 20 min after administration and within 1 h before administration on Day 36.

- Week 8: D50 48h ± 2h
- Sparse sampling in Week 9: within 1 h before administration on Day 57, 4 h ± 10 min, 8 h ± 20 min after administration and within 1 h before administration on Day 64.
- Sparse sampling at Week 12: within 1h before administration, within 48h ± 2h after administration (D80) and within 168h ± 12h after administration (D85) on D78;
- PK sampling will be collected at the Early Termination Visit if a patient discontinues early due to failure to complete the study.

7.3.2 PD (Insulin/C-peptide/Plasma Glucose/Glucagon/OXM/GLP-1) Blood Collection Points:

- Standard meal test: During the screening period D-1, sampling points include fasting, 30 min, 1h, 2h, 3h and 4h;
- Week 1: within 48 h ± 2 h (Day 3) after administration and within 1 h before administration on Day 8;
- Week 5: Within 1 h before administration on Day 29;
- Week 9: Within 1 h before administration on Day 57;
- Week 12: Within 1 h prior to D78 administration and 168 h ± 12 h after administration (D85);
- Standard meal test: At week 12 on D80, sampling points included fasting, 30 min, 1 h, 2 h, 3 h and 4 h;
- PD samples will be collected at the Early Termination Visit if a patient is withdrawn early due to failure to complete the study. All PD sampling will be performed in the fasted state.

7.3.3 HbA1c Blood Sampling Points:

- Within 1 h prior to dosing on D1 of Week 1;
- Within 1 hour before dosing on D29 in Week 5;

- ➢ Within 1 hour before D57 administration in Week 9;
- ➢ 168h ± 12h on D78 at week 12 (D85);
- ➢ Samples will be collected at the early termination visit if the patient is withdrawn prematurely due to failure to complete the study;
- ➢ Blood samples will be collected under fasting conditions.

7.3.4 PK/PD Measurements

- (3) Kinetic parameters, including but not limited to: Tmax, Cmax, area under the concentration-time curve (AUC), volume of distribution (Vd), half-life (T1/2), clearance (CL), accumulation coefficient (AR);
- Pharmacodynamic parameters: change before and after administration of fasting plasma glucose, glucagon, insulin, fasting C-peptide, HbA1c, endogenous OXM, GLP-1
- MTT assay

7.4 Immunogenicity Assessment Indicators

7.4.1 Immunogenicity Blood Sampling Points

- Within 1 hour prior to initiation of IBI362 dosing in Week 1;
- Week 5 (within 1 h before administration on Day 29);
- Week 9 (within 1 h before administration on Day 57);
- Week 13 (168h ± 12h for Week 12);
- At safety follow-up;
- If a patient withdraws early, an immunogenicity sample should be collected at the early withdrawal visit whenever possible.

7.4.2 Immunogenicity indicators

- Anti-IBI362 antibody (ADA) and neutralizing antibody (NAb) in serum;

8. safety reporting and adverse event management

8.1 Definition of Adverse Events

An adverse event (AE) is defined as any untoward medical occurrence, whether or not there is a causal relationship with the study drug, in a clinical study subject from the time informed consent form is signed through 90 days after the last dose of study drug, and includes, but is not limited to, the following:

- Exacerbation of pre-existing (prior to clinical study) medical conditions/diseases (including worsening of symptoms, signs, laboratory abnormalities);

- Any newly developed adverse medical conditions (including symptoms, signs and newly diagnosed diseases);
- Clinically significant abnormal laboratory values or results.

8.2 Serious Adverse Events Definition of

A serious adverse event is an adverse event that meets at least one of the following criteria:

- Results in death;
- Is life-threatening ("life threatening" is defined as an AE that places the subject at risk of death from the event as it occurred, and does not include an AE that, had it occurred in a more severe form, might have caused death);
- Requires inpatient hospitalization or prolongation of hospitalization, excluding the following:

a) Rehabilitation facilities;

b) Nursing homes;

c) Routine emergency room admission;

d) Same-day surgery (e.g. outpatient/same-day/ambulatory surgery);

e) Hospitalization or prolongation of hospitalization not associated is unrelated to SAE itself. For example, hospitalization due to original diseases and there are were no new adverse events and aggravation of the original diseases (to check for laboratory abnormalities that persisted even before the study trial); hospitalization for management reasons (e.g. annual routine physical examination); hospitalization during the clinical trial as specified in the study protocol (e.g. procedures required according to the study protocol); and hospitalization that is unrelated to adverse events (e.g. elective surgery); scheduled treatment or surgery, which should be documented in the entire trial protocol and/or the baseline data of the patient; hospitalization only to use blood products.

- Results in persistent or significant disability/incapacity;
- Results in a congenital anomaly/birth defect.

Other important medical events: defined as events that jeopardize the subject or require medical intervention to prevent one of outcomes listed above.. For example, major treatment in the emergency room or allergic bronchospasm at home, cachexia or convulsion without hospitalization, drug dependence or addiction.

8.3 Adverse Events of Special Interest (AESI)

The following adverse events are of special interest in this study: allergic

reactions, injection site reactions, elevated transaminases, elevated creatinine, prolonged QTc interval, gastrointestinal reactions (nausea, vomiting, decreased appetite), hypoglycemia, and acute pancreatitis, and severe AEs that are not SAEs related to study drug. In this clinical study, the investigator should pay close attention to whether the patient has the above adverse events and handle them in time.

The above listed AESIs need to be reported to the sponsor within 24 hours of awareness (i.e. drugsafety@innoventbio.com), if the AESI meets the SAE criteria and is also reported according to the SAE reporting process

8.3.1 Gastrointestinal Reactions

Nausea, vomiting, and diarrhea are adverse events of interest and will be recorded as AEs on the eCRF. Each event will be assessed for severity, length (start and stop date), and relationship to study drug or protocol procedure as deemed by the investigator. (Grading of gastrointestinal reactions 4)

Table 4. Grading of Gastrointestinal Reactions (CTCAE Criteria)

**Gastrointestinal Reactions**	**CTCAE Grade**				
	**Level 1**	**Grade 2**	**Grade 3**	**Grade 4**	**Grade 5**
Nausea	Decreased appetite without change in eating habits	Reduced food intake without significant weight loss, dehydration, or malnutrition	(3) Inadequate intake of energy and water; nasogastric feeding, total parenteral nutrition, or hospitalization required	-	-
Vomiting	No intervention required	Outpatient intravenous hydration; medical intervention indicated	Need for nasogastric feeding, total parenteral nutrition or hospitalization	Life Threatening	Death
Diarrhea	Increase in stool frequency < 4 stools per day compared to baseline; mild increase in ostomy discharge	4 to 6 stools per day more frequent than at baseline; moderate increase in ostomy output; limiting activities of daily living with tools	≥ 7 stools per day increase from baseline; hospitalization indicated; severe increase in ostomy output compared with baseline; limiting self-care activities of daily living	Life-threatening; urgent treatment indicated	Death

8.3.2 Acute Pancreatitis

Serum amylase and/or lipase will be monitored at protocol-specified time points, and additional tests may be added at the discretion of the investigator.

When amylase and/or lipase will be ≥ 3 × ULN, further diagnostic evaluation will be required even if the patient will be not symptomatic of acute pancreatitis (Table 5).

Table 5. Patients without symptoms of pancreatitis will be safely monitored by pancreatic enzyme-related indicators1, 2
(Applicable when serum lipase and/or amylase are ≥ 3 times ULN)

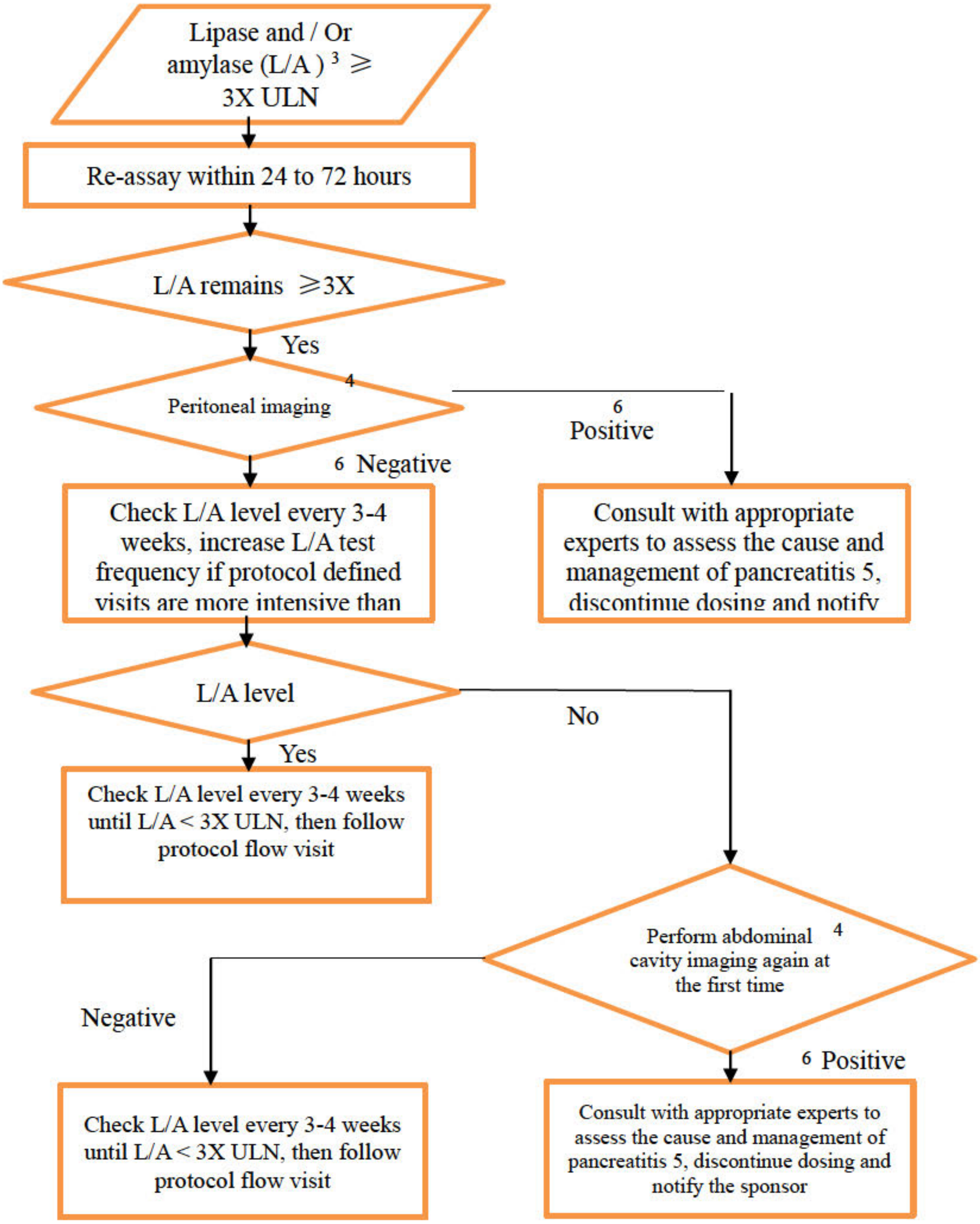

1. Symptomatic mainly refers to abdominal pain associated with pancreatitis, severe nausea, vomiting, and other symptoms can also be considered as related symptoms by the investigator.
2. If the investigator believes that a patient has symptoms of acute pancreatitis any time, regardless of whether based on L/A test results, the investigator should consult a specialist for evaluation, management, assessment of the cause of pancreatitis, discontinuation of dosing and notification of the sponsor.
3. Either or both of lipase and amylase can be used as the evaluation criteria of this algorithm.
4. The optimal time for abdominal imaging is immediately after the enzyme elevation has just been detected. If the investigator or imaging physician judges that it is safe, it is best to use enhanced abdominal CT for examination, or MRI.
5. At a minimum, liver function tests, triglycerides, and ionized calcium levels will be performed and all concomitant medications will be recorded.
6. Negative and positive imaging results are used to indicate acute pancreatitis.

8.3.3 Hepatic dysfunction events

If abnormal AST and/or ALT levels are complicated by abnormal elevation of total bilirubin levels, drug-induced liver injury will be considered if the following conditions are met and there is no other cause for liver injury. Such situations should always be considered important medical events.

Table 6 Hepatic Impairment to be Reported as an SAE

**Baseline Period**	Normal (AST/ALT and total bilirubin)	Abnormal (AST/ALT and total bilirubin)
**Treatment period**	ALT or AST ≥ 3 × ULN<br>With total bilirubin ≥ 2 × ULN<br>AND alkaline phosphatase ≤ 2 × ULN<br>And no hemolysis	AST or ALT ≥ 8 × ULN<br>With total bilirubin increase ≥ 1 × ULN or total bilirubin value ≥ 3 × ULN

Patients should return to the study site for evaluation as soon as possible (preferably within 48 hours) after acknowledge of the abnormal results. Assessments should include laboratory tests, detailed history and physical assessment. The abnormality should be considered the the relationship to liver tumors (primary or secondary).

In addition to repeating AST and ALT, laboratory tests to be performed include albumin, creatine kinase, total bilirubin, direct and indirect bilirubin, γ-glutamyltransferase, prothrombin time/international normalized ratio, and alkaline phosphatase. Detailed history collection should include: history of drinking, acetaminophen, soft drugs, various supplements, traditional Chinese medicines, history of exposure to chemical drugs, family history, occupational exposure, sexual behavior, travel history, history of exposure to jaundiced patients, surgery, blood transfusion,

history of liver or allergic diseases, history of heart disease, history of immune diseases, etc.. Further examinations may also include the detection of acute hepatitis A, B, C and E, liver imaging examination (such as biliary tract), autoantibodies and cardiac ultrasound. If repeated testing still confirms compliance with the laboratory criteria defined in Table 6, the possibility of potential drug-induced liver injury should be considered in the absence of other causes of abnormal liver function tests, without waiting for all etiologic liver function test results. Cases of such potential drug-induced liver injury should be reported as SAEs.

8.3.4 Hypoglycemic Events

Hypoglycemia is classified by severity as follows:

- **Severe hypoglycaemia:** refers to a condition where the patient requires the assistance of another person to obtain carbohydrate, glucagon or other rescue measures. Hypoglycaemic episodes may be accompanied by neuroglycaemic symptoms and, in severe cases, precipitate epileptiform convulsions or coma. Such events may not have been documented by blood glucose measurements, but recovery of neurological symptoms as blood glucose returned to normal is sufficient evidence that the event is caused by hypoglycemia.
- **Documented symptomatic hypoglycemia:** typical symptoms of hypoglycemia with a plasma glucose concentration less than or equal to 70 mg/dL (3.9 mmol/L).
- **Asymptomatic hypoglycaemia:** Asymptomatic hypoglycaemia are not shown with typical symptoms, but with a plasma glucose concentration less than or equal to 70 mg/dL (3.9 mmol/L).
- **Probable symptomatic hypoglycemia:** during the study, the symptoms of hypoglycemia are not accompanied by a plasma glucose determination, but symptoms are presumed to be caused by a plasma glucose concentration less than or equal to 70 mg/dL (3.9 mmol/L).
- **Relative hypoglycaemia:** The typical clinical symptoms of hypoglycaemia reported by a person with diabetes mellitus who considers himself/herself suffering from hypoglycaemia but with a measured blood glucose concentration greater than 70 mg/dL (3.9 mmol/L).

The investigator will determine whether a hypoglycaemic episode is severe or not based on the need for medical assistance in addition to the intended assistance that the patient normally receives. All hypoglycaemic episodes will be recorded in the hypoglycaemic module of the eCRF and all severe hypoglycaemic episodes must be reported as SAEs.

Blood glucose self-monitoring

During the non-inpatient study period, patients will be required to record the self-inspection blood glucose before meals, 2 hours after each meal and at bedtime (7 times in total). These data should be collected and sorted out within 3 days prior to the scheduled clinical visit. The patients will record their self-measured blood glucose daily as instructed, and the complete data of 7 measurements needs to be collected on each day. If the patient fails to complete the data record for one day, the whole data record for 7 times shall be completed in the next day.

8.3.5 Severe adverse events that are related to the study drug and are not serious adverse events, regardless of whether the event is associated with the same organ or system.

8.4 Adverse Events Severity Assessment

The investigator will judge the severity according to the grading criteria for adverse events issued by the National Institute on Aging (NIA).

- Mild: Awareness of a symptom or sign that is easily tolerated and of minor irritation that does not interfere with normal activity, and that does not require treatment or medical evaluation; symptoms and signs are transient;
- Moderate: An event that causes a low degree of inconvenience or concern to the patient and may interfere with daily activities, but usually improves with simple therapeutic measures; moderate adverse events may cause some functional impairment;
- Severe: The event interrupts the patient's normal daily life and usually requires systemic drug therapy or other therapy, and usually results in disability.

8.5 Causal relationship between adverse events and investigational drug

The investigator must assess the causal relationship between the study drug and each AE and answer "yes" or" no" to the question "There is a reasonable possibility that the AE occurred due to the study drug".

When it is determined that there is a "reasonable possibility" that an AE is due to the drug, the following factors should be considered:

- Time course. Exposure to suspect drug. Did the subject actually receive treatment with the suspect drug? Is there a reasonable temporal relationship between the onset of the AE and the suspect drug?
- Consistency of known drug properties. Is the AE consistent with previously reported events for the suspect drug (pharmacology and toxicology) or drugs of the same pharmacological class? Is the occurrence of AEs expected from the

pharmacological properties?

- Dechallenge. Did the AE resolve or improve after stopping or reducing the suspect medication?
- No alternatives. The AE cannot be reasonably explained by another pathology, such as underlying disease, other drugs, other intrinsic or environmental factors.
- Rechallenge. Did the AE reappear after the suspect drug was used after stopping?
- There may be other reasons. Can the adverse event not be explained by an alternative etiology, such as underlying disease, other drugs/vaccines, or other host or environmental factors?

When one or more factors are present, the “reasonable possibility” of the AE needs to be considered.

On the contrary, if the above criteria do not apply, if there is no clear evidence of exposure and a reasonable time course, or if any challenge (if performed) is negative or there is another possible cause of the AE, there may be no "reasonable possibility” for a causal relationship.

Based on the above factors, the relationship between the study drug and the AEs or the role in the adverse event is classified into the following two types by the investigator assessment:

• Related, i.e., consider that there is a reasonable possibility that the study drug is associated with the occurrence of an AE: evidence of exposure to the study drug. The temporal sequence between the onset of the AE and administration of the Sponsor product is reasonable. The AE is more likely to be explained by the study drug than by other causes;

• Unrelated, i.e., it is considered that there is not a reasonable possibility that the study drug is related to the occurrence of AE: for example, the patient does not start the study drug; or there is no reasonable temporal relationship between the occurrence of AE and the exposure of study drug; or other factors that may explain the occurrence of AE compared with the study drug.

8.6 Recording of Adverse Events

Investigators should use medical terminology/concepts to record AEs or SAEs. Use of colloquialisms and abbreviations should be avoided. All AEs (including SAEs) should be recorded on the Adverse Event form of the eCRF.

8.6.1 Timely collection of adverse events

The investigator learns of an adverse event by asking the patient a non-inductive question.

All AEs, including SAEs, whether observed by the investigator or spontaneously reported by the patient, will be collected from the signing of the ICF through the end of the study. After the end of the study, if the investigator learns of a patient's serious adverse event related to the study drug or procedure, it should also be reported to the sponsor.

8.6.2 Follow-up of Adverse Events

Adverse events should be followed until recovery/return to baseline or the investigator considers no further follow-up necessary for reasonable reasons (eg, can not recover or has improved). If the adverse event does not recover, a reasonable explanation is required. The relatives to the study drug, the recovery of AE or SAE and its date should be recorded in eCRF and medical record.

8.6.3 Contents of adverse event records

The investigator should completely record any AE, including diagnosis (if no diagnosis, record the symptoms and signs including laboratory abnormalities), start and stop time (if applicable), severity and changes, whether it is an SAE, actions taken for the study drug, treatment given due to AE and the outcome of the event, and the relationship between the AE and the study drug.

For serious adverse events, the investigator provided the date on which the AE met the criteria for an SAE, the date that the investigator became aware of the SAE, the rationale for determining the AE as an SAE, the date of hospitalization, the date of discharge, the possible cause of death, the date of death, whether an autopsy will be performed, the assessment of causality with the study procedures, the assessment of causality with other drugs, and other possible causes of the SAE. The investigator should provide the basis for judging the correlation and the description of SAE. The description of an SAE should also include the patient's number, age, gender, height and weight; the patient's indications for the investigational drug treatment, disease stage and relevant general conditions; the clinical course of the SAE including its occurrence, development, outcome and results; laboratory test results related to the SAE (the test time, unit and normal range must be provided); past history related to the SAE, concomitant diseases and their occurrence and duration; medication history related to the SAE, concomitant drugs and their start, duration, dosage and administration; and details about the start, duration, dosage and administration of the investigational drug treatment.

Notes regarding recording of AEs are described below:

Diagnosis, Symptoms and Signs

If a diagnosis has been made, the diagnosis should be recorded in the eCRF rather than individual signs and symptoms (e.g., record liver failure rather than jaundice, elevated transaminases, and flapping tremor). If symptoms and signs cannot be determined to be caused by the diagnosis at the time of reporting, they should be recorded

as a separate AE/SAE. If it is determined that the symptoms and signs are caused by the diagnosis, only the diagnosis is reported separately, and the symptoms and signs are included in the diagnosis. The records of symptoms and signs should be deleted for AEs, and follow-up update reports should be sent for SAEs.

Adverse Events Secondary to Other Events

In general, AEs secondary to other events (e.g., caused by other events or clinical sequelae) should have their primary events recorded, unless the secondary events are severe or serious adverse events. However, secondary events with significant clinical significance should be recorded as independent adverse events in eCRF if they occur at a different time from the primary event. If the relationship between the events is unclear, it should be recorded separately in the eCRF.

Ongoing or recurrent adverse events

A persistent AE is one that persists without resolution between evaluations.

A recurrent adverse event will be one that resolved between evaluation time points but subsequently recurred. The occurrence of the event should be recorded separately in the eCRF.

Laboratory Abnormalities

Clinically significant laboratory abnormalities should be reported as AEs. It is the responsibility of the investigator to review all abnormal laboratory findings and make medical judgment as to whether each abnormal laboratory finding should be reported as an AE.

Pre-existing medical conditions

The preexisting symptoms/signs a patient has already presented during the screening period of the trial should be recorded and reported as AEs only when there is worsening in severity, frequency or nature (except for worsening of the medical condition under study) after entry into the trial. Changes from the previous state will be reflected in the record, such as "increased frequency of headache".

8.7 Expedited reporting of SAEs and pregnancies

SAE Reporting:

If a SAE occurs to a patient from the time the patient signs the informed consent form, the investigator must immediately complete, sign, and date the Serious Adverse Event Report Form and immediately report to the Sponsor's Pharmacovigilance Department within 24 hours of the investigator's awareness: Drugsafety@innoventbio.com For death and life-threatening serious adverse events, the investigator should urgently follow up the missing information and provide a complete SAE report.

Meanwhile, after being informed of the occurrence of SAE, the investigator should report to the drug regulatory authorities, health administrative authorities and Ethics Committee according to the regulatory requirements.

After the end of the study, if the investigator learns of a patient's serious adverse event related to the study drug or procedure, it should still be reported to the sponsor.

Pregnancy

All patients of childbearing potential participating in a clinical trial must use effective contraception.

If a female patient exposed to the drug becomes pregnant during the clinical trial, the patient should be withdrawn, and the pregnancy should be reported to the sponsor within 24 hours after the investigator learns of the pregnancy, and Innovent Clinical Trial Pregnancy Report/Follow-up Form should be completed.

If the partner of a male patient exposed to the drug becomes pregnant during the clinical trial, the patient may continue the clinical trial, report the pregnancy to the sponsor within 24 hours after the investigator learns of the pregnancy, and complete Innovent Clinical Trial Pregnancy Report/Follow-up Form.

The investigator should continuously monitor the patients with pregnancy and follow up the pregnancy results until 8 weeks after delivery, and report the results to the sponsor.

If the outcome of pregnancy is still birth, spontaneous abortion, fetal malformation (any congenital anomaly/birth defect) or induced abortion for medical reasons, it should be considered as SAE and reported according to the procedures and time limit for SAE reporting.

If a patient experiences a concurrent SAE during pregnancy, it should be reported according to the SAE reporting procedure.

9 Data analysis/statistical methods

9.1 Statistical Hypotheses

The primary endpoints of this study are the safety and tolerability of IBI362. No formal statistical testing will be performed.

9.2 Estimation of Sample Size

Enrollment is planned in 3 dose cohorts. A total of 14 patients are planned to be enrolled in each cohort and randomized to IBI362, placebo, and active control as 8:4:2. Approximately 42 patients will be enrolled in total.

9.3 Statistical Analysis Population

Safety Set: all enrolled subjects who received at least one dose of study drug.

Efficacy Analysis Set: patients in the Safety Set who experienced at least 1 post-baseline assessment.

PK concentration set: all the patients who have received at least one dose of the study drug and have at least one data of effective serum concentration after administration.

PD analysis set: all the patients who have received at least one dose of the study drug and have at least one dose of study drug and have at least one data of valid post-dose PD parameter.

9.4 Statistical Analysis

9.4.1 General Methods

Measurement data will be described by mean, standard deviation, median, maximum and minimum. Continuous variables will be described by frequency and percentage. Unless otherwise specified, the data of patients in each IBI362 dose group and all patients in placebo and active control groups will be analyzed separately.

All statistical analyses will be completed using SAS 9.2 (or higher).

9.4.2 Efficacy Analysis

All efficacy analyses will be performed on the efficacy analysis set. Efficacy endpoints will be analyzed only descriptively. For continuous efficacy endpoint indicators, the corresponding means and 95% confidence intervals will be calculated for IBI362 g placebo and active control groups, seperately. A two-sample t-test will be used to calculate the p-value of IBI362 versus placebo for each dose group and provide the corresponding point estimate and 95% confidence interval of the difference. LOCF missing values will be used for imputation. For the classified efficacy endpoints, the ratio within each dose group will be calculated and the 95% confidence interval within the group will be calculated using Clopper-Pearson; the difference between IBI362 placebo groups will be compared using the chi-square test and the corresponding 95% confidence interval will be calculated. Missing values for grouped endpoints will be imputed using the default non-response method.

9.4.3 Safety Analysis

Safety analyses will be performed in a secure set.

Adverse events:

The number of patients with each AE will be coded by Medical Dictionary for Regulatory Activities (MedDRA) system organ class, MedDRA preferred term, and adverse event grade. In addition, the severity and relationship of TEAEs to study drug will also be summarized by SOC, PT, and arm.

Laboratory tests:

For hematology, blood biochemistry, blood lipid, coagulation function, urinalysis, myocardial enzyme spectrum, thyroid function, serum amylase and/or lipase, calcitonin and vital signs (pulse, respiration, blood pressure and body temperature), the measured values and change values before and after treatment will be described using mean ± standard deviation, maximum, minimum and median, and cross classification table will be used to describe normal and abnormal changes before and after treatment.

Urinalysis: cross classification table will be used to describe normal and abnormal changes before and after treatment.

Exploratory analyses of ECG parameters will be performed.

The proportion of patients with clinically significant abnormality changes will be described, and whether the abnormality is clinically significant will be judged by the investigator.

Other:

Details of death will be listed.

Describe the measurements and changes in ECG therapy. Cross-classification tables will be used to describe normal and abnormal changes before and after treatment. Changes in vital signs will be summarized descriptively.

9.4.4 Immunogenicity

Anti-drug antibodies (ADA) and neutralizing antibodies (NAb) will be summarized using descriptive statistics. Antibody levels in positive subjects will be listed.

9.4.5 Analysis of kinetic and pharmacodynamic parameters

Each PK parameter, including but not limited to T_{max}, C_{max}, area under the curve (AUC), volume of distribution (Vd), half-life (T1/2), clearance (CL), and accumulation coefficient (AR), will be summarized for each dose cohorts separately.

Pharmacodynamics parameters will be summarized, including: fasting blood glucose, glucagon, insulin, fasting C-peptide, HbA1c, endogenous OXM, and GLP-1 measurements at baseline and at various time points after drug administration, and changes from baseline at each time point will be summarized.

9.4.6 Exploratory Analysis

Changes from baseline in HOMA-Beta, HOMA-IR, weight in fasting, waist circumference, and BMI will be summarized at each time point after treatment.

9.5 Multiplicity Comparison

Not applicable.

10 Quality assurance and quality control

Each study site will implement internal quality management for the study process,

data and biological sample collection, document archiving and completion. Individualize the quality management plan for each institution.

The quality control (QC) step starts with the data entry system and data QC checks will be run on the generated database. If any missing or abnormal data is found, the study site will be notified for clarification or judgment.

The monitor will verify that the study is conducted in compliance with the protocol, International Conference on Harmonisation Good Clinical Practice (ICH GCP) and applicable regulatory requirements [e.g., Good Laboratory Practice (GLP), Good Manufacturing Practice (GMP)], including the study process, generation/collection, archiving (recording), reporting of data and biological samples, etc., according to written Standard Operating Procedures (SOPs).

The site will provide direct access to all study-related units, source data/documents, and reports for activities such as sponsor monitoring, audits, or inspection by local regulatory authorities.

11 Data management and storage

The study staff will be responsible for data collection. The investigator should monitor it and ensure the accuracy, completeness, readability, and timeliness of the reported data.

All source documents should be kept clear and tidy to ensure accurate identification of the data.

A permanent copy of the study visit record will be considered the source document to record the data for enrolled patients. Data recorded on the electronic case report form (eCRF) should be derived from source documents and be consistent with the source data.

In regions where International Conference on Harmonisation (ICH) regulations apply, study documentation should be retained for at least 2 years after the last marketing application is approved. There are no pending or new marketing applications in the region; or for at least 2 years after the formal discontinuation of study intervention. These documents may be retained for a longer period if required by local regulations. If applicable, no materials may be destroyed without the written consent of the sponsor. It is the responsibility of the sponsor to inform the investigator of the termination and storage date of the above documents.

12 Ethics

12.1 Ethics Committee

The sponsor is obliged to obtain approval from the Ethics Committee for the study protocol and its amendments, the patient informed consent form and other relevant

documents, such as recruitment advertisements (if used). All communication records with the Ethics Committee will be kept in the Investigator File. The materials reviewed and approved by the Ethics Committee will be delivered to the Sponsor.

12.2 Ethical Conduct in the Study

This study will strictly comply with the provisions of the study protocol, requirements of laws and regulations as well as the principles of international ethical guidelines, and comply with ICH GCP guidelines and the Declaration of Helsinki.

12.3 Patient Informed Consent

An informed consent detailing the study intervention, study procedures, and risks will be given to the patient, and a written informed consent document will be obtained prior to the start of the study intervention/medication. The following informed consent materials will be submitted with the protocol.

Informed consent should be signed before an individual agrees to participate in the study and continues throughout their participation in the study. The informed consent form will be approved by the EC and the patient will be asked to read and review the document. The investigator is responsible for explaining the study to the patient and answer any questions the patient may have. The investigator will verbally explain to the patient in a manner appropriate to the patient's understanding, including the purpose of the study, the procedures and potential risks, and the rights as a patient. The patient should have ample time to read and ask questions carefully before signing the written informed consent. Patients should have the opportunity to discuss the study with their family or designee or to consider themselves before agreeing to participate. Patients will sign an informed consent form prior to performing any procedures specific to the study. Patients must be informed that participation is voluntary and that they can withdraw from the study at any time without prejudice. The investigator will provide the patient with a copy of the informed consent document for retention. The informed consent process should be conducted before the patient has undergone any procedures for the study, and the process (including dates) of informed consent should be documented in the source documents, as well as the retention of the signed informed consent form. In order to ensure the rights and welfare of the patients, the investigator must specifically inform the patients that "if they refuse to participate in this study, their quality of medical care will not be adversely affected".

12.4 Protocol Violations

A protocol deviation is any noncompliance with the clinical trial protocol, International Conference on Harmonisation Good Clinical Practice (ICH GCP), or Manual of Operations. Non-compliance may arise from the subject, investigator, or site staff. Corrective actions shall be taken and completed in a timely manner in response to the deviation.

13 Publication policy

All data generated from this study are confidential information of the Sponsor. The Sponsor has the right to publish the study results. Information regarding the publication policy between the Sponsor and the investigator will be described in the clinical trial agreement.

All information about this trial (not limited to the following documents: protocol, investigator's brochure) must be kept strictly confidential. The investigator must recognize that the scientific or medical information derived from this trial may be of commercial value to the Sponsor. The investigator shall keep confidential the information and data related to this trial. In order to publish the information related to this trial or the conclusions drawn from the trial, the investigator shall negotiate with the Sponsor in advance and obtain the written consent of the Sponsor. In order to protect its own rights and interests, the Sponsor may require the investigator not to publish information about the trial before the investigational product is approved for marketing.

The Sponsor has the right to publish the information or data related to this trial or submit it to the drug regulatory authority. If the Sponsor needs to include the investigator's name in a publication or advertisement, the consent of the investigator should be obtained.

14 Protocol Revision History

Version No.	DATE	Description of Change
V1.0	03/19/2020	Not applicable for a new protocol
V1.1	2021/01/15	See Protocol Amendment Record

15 REFERENCES

[1] Pocai A. Action and therapeutic potential of oxyntomodulin. Mol Metab. 2013; 3 (3): 241-251.

[2] Tan TM, Field BC, McCullough KA, Troke RC, Chambers ES, Salem V, Gonzalez Maffe J, Baynes KC, De Silva A, Viardot A, Alsafi A, Frost GS, Ghatei MA, SR.Cooration of glucagon-like peptide-1 infusion in humans during results in increased energy expenditure and amelioration of increased energy expenditure Hyperglycemia. Diabetes. 2013; 62 (4): 1131-1138.

[3] Campbell JE, Drucker DJ.Nature ReviewsEndocrinology, 2015, 11 (6): 329-338 ·

[4] Baldissera, F.G., Holst, J.J., Knuhtsen, S., Hilsted, L., & Nielsen, O.V., 1988. Oxyntomodulin (glicentin- (33 – 69)): pharmacokinetics, binding to liver cell membranes, effects on perfused pig pancreas, and secretion from isolated lower intestine of pigs. Regulatory Peptides 21:151 – 166.

[5] Gros, L., Thorens, B., Bataille, D., & Kervran, A., 1993. Glucagon-like peptide-1-(7 – 36) amide, oxyntomodulin, and glucagon interact with a common receptor in a somatostatin-secreting cell line. Endocrinology 133:631 – 638.

[6] Pocai, A., Carrington, P.E., Adams, J.R., Wright, M., Eiermann, G., Zhu, L., et al. 2009. Glucagon-like peptide 1/glucagon dual receptor agonism reverses obesity in mice. Diabetes 58:2258 – 2266.

[7] Baggio, L.L., Huang, Q., Brown, T.J. & Drucker, D.J., 2004. Oxyntomodulin and glucagon-like peptide-1 differentially regulate murine food intake and energy expenditure. Gastroenterology 127:546 – 558.

[8] Jorgensen, R., Kubale, V., Vrecl, M., Schwartz, T.W., & Elling, C.E., 2007. Oxyntomodulin differentially affects glucagon – like peptide – 1 receptor b-arrestin recruitment and signaling through G_ (s). Journal of Pharmacology and Experimental Therapeutics 322:148 – 154.

[9] Schepp, W., Dehne, K., Riedel, T., Schmidtler, J., Schaffer, K., & Classen, M., 1996. Oxyntomodulin: a cAMP-dependent stimulus of rat parietal cell function via the receptor for glucagon-like peptide-1 (7 – 36) NH2. Digestion 57:398 – 405.

[10] Seino Y 1, Rasmussen MF, Zdravkovic M, Kaku K. Dose-dependent improvement in glycemia with once-daily liraglutide without hypoglycemia or weight gain: A randomized, controlled trial in Japanese patients with type 2 diabetes. Diabetes Res Clin Pract. 2008 Aug; 81 (2): 161-168.

[11] Wenying Yang journal, prevalence of Diabetes among Men and Women in China [J], The new england journal o f medicine, 2010, 362, 12:1090-1101

[12] Xu Y, Wang L, He J, et al. Prevalence and control of diabetes in Chinese adults [J]. JAMA, 2013, 310 (9): 948-958

[13] Xu Y, Wang L, He J, et al. Prevalence and control of diabetes in Chinese adults [J]. JAMA, 2013, 310 (9): 948 [J] 959. DOI: 10. 1001/jama. 2013. 168118.

[14] Yang W, Lu J, Weng J, et al. Prevalence of diabetes among men and women in China [J]. N Engl J Med, 2010, 362 (12): 1090 [J] 1101.

[15] Hou X, Lu J, Weng J, et al. Impact of waist circumference and bodymass index on risk of cardiometabolic disorder and cardiovascular disease in Chinese adults: a national diabetes and metabolic disorders survey [J]. PLoS One, 2013, 8 (3): e57319.

[16] Chinese Diabetes Society. Chinese Guidelines for the Prevention and Treatment of Type 2 Diabetes (2013 Edition) [J]. Chinese Journal of Endocrinology and Metabolism, 2014, 30 (10): 893 [J] 942.

[17] Chinese Society of Endocrinology. Expert consensus on comprehensive management of type 2 diabetes mellitus complicated with obesity in China [J]. Chinese Journal of Endocrinology and Metabolism, 2016, 32 (9): 623-627.

[18] Pocai A. Unraveling Oxyntomodulin, GLP1's enigmatic brother. J Endocrinol. 2012; 15:335-346.

[19] Day JW, Ottaway N, Patterson JT, et al. A new glucagon and GLP-1co-agonist eliminates obesity in rodents. Nat Chem Biol. 2009; 5:749-757.

[20] Kosinski JR, Hubert J, Carrington PE, Chicchi GG, Mu J, Miller C, Cao J, Bianchi E, Pessi A, Sinharoy R et al. The 2012 The glucagon receptor is involved in mediating the body weight lowering effects of oxyntomodulin. Obesity 20 1566 – 1571

[21] Karra E & Batteram RL 2010 The role of gut hormones in the regulation of body weight and energy homeostasis. Molecular and Cellular Endocrinology 316 120 – 128.

[22] Maida A, Lovshin JA, Baggio LL & Drucker DJ 2008 The glucagon-like peptide-1 receptor agonist enhances b-cell function but does not inhibit gastric emptying in mice. Endocrinology 149 5670 – 5678.

[23] Parliet ET, Heijbolever AC, Schroder-van der Elst JP, Havekes LM, Romijn JA, Pijl H & Corssmit EP 2008 Oxyntomodulin ameliorates glucose intolerance in fed mice

a high-fat diet. American Journal of Physiology. Endocrinology and Metabolism 294 E142 – E147.

[24] Du X, Kosinski JR, Lao J, Shen X, Petrov A, Chicchi GG, Eiermann GJ & Pocai A 2012 Differential effects of oxyntomodulin and GLP-1 on glucose metabolism. American Journal of Physiology. Endocrinology and Metabolism 303 E265 – E271.

[25] Flamez D, Gilon P, Moens K, Van Breusegem A, Delmeire D, Scrocchi LA, Henquin JC, Drucker DJ & Schuit F 1999 Altered cAMP and Ca2C signaling in mouse pancreatic islets with glucagon-like peptide-1 receptor null phenotype. Diabetes 48 1979 – 1986.

[26] Mighiu PI, Yue JT, Filippi BM & Lam TK 2012 Hypothalamic glucagon signaling regulates glucose production. Diabetes 61 (Suppl 1) A55. Nauck MA 2012 The design of the liraglutide clinical trial programme. Diabetes, Obesity and Metabolism 14 (Suppl 2) 4 – 12.

[27] Kosinski JR, Huber J, Carrington PE, et al. The glucagon receptor is involved in mediating the body weight-lowering effects of oxyntomodulin. Obesity. 2012; 20:1566-1571.

[28] Lao J, Hansen BC, DiMarchi R, et al. Effect of GLP1R/GCGR dual agonist in monkeys. Diabetes. 2013; 62 (suppl 1): A257.

[29] Ralf Elvert, Andreas W. Herling. Running on mixed fuel-dual agonistic approach of GLP-1 and GCG receptors leads to impact on body weight and blood glucose control: A comparative study between mice and non-human primate. Diabetes Obes Metab. 2018; 20:1836 – 1851.

[30] Jorgensen R, Kubale V, Vrecl M, Schwartz TW & Elling CE 2007 Oxyntomodulin differentially affects glucagon – like peptide – 1 receptor β-arrestin recruitment and signaling through Ga (s). Journal of Pharmacology and Experimental Therapeutics 322 148 – 154.

Supplementary Note 2:

Statistical Analysis Plan

Statistical Analysis Plan

A Multiple Dose Tolerability and Pharmacodynamic Study of IBI362 in Chinese Patients with Type 2 Diabetes and Poor Glycemic Control

Protocol No.: CIBI362A101

Version No.:	**1.0**
Version Date:	**June 30, 2021**
Statistician:	**Feng Liqi**

SIGNATURE PAGE

[redacted]

[redacted]

[redacted]

[redacted]

[redacted]

[redacted]

[redacted]

[redacted]

VERSION HISTORY

SAP Version No.	Approval date	Description of Change
1.0	6/30/2021	First version

ABBREVIATION

Abbreviation	English full name
ADA	Anti-drug antibody
AE	Adverse Event
AESI	Adverse Event of Special Interest
ALB	Albumin
ALP	Alkaline phosphatase
ALT	Alanine transaminase
APTT	Activated partial thromboplastin time
AR	Accumulation Ratio
AST	Aspartate amino transferase
AUC	Area Under Curve
CK	Creatine Kinase
CL	Clearance
Cmax	Maximum serum concentration of drug
Cr	Creatinine
DBIL	Direct bilirubin
EC	Ethics Committee
ECRF	Electronic case report form
FPG	Fasting plasma Glucos
FT3	Free Triiodothyronine
FT4	Free Thyroxine
GCGR	Glocagon receptor
GCP	Good clinical practice
GGT	Gamma-Glutamyl transpeptidase
GLP-1	Glucagon-like peptide-1
GLP-1R	Glucagon-like peptide-1 receptor
GMP	Good manufacturing practice
HbA1c	Hemoglobin A1c
HCT	Hematocrit
HCV	Hepatitis C virus
HDL-C	High density liptein cholesterol
HGB	Hemoglobin
HIV	Human Immunodeficiency Virus
HOMA-Beta	Homeostasis model assessment-Beta
HOMA-IR	Homeostasis model assessment-insulin resistance
HR	Heart Rate
ICF	Informed consent form
ICH	International Conference on Harmonisation
IDF	International Diabetes Federation
INR	International normalized ratio
LDH	Lactate dehydrogenase
LDL-C	Low density liptein cholesterol
MedDRA	Medical Dictionary for Regulatory Activities
MTD	Maximum tolerated dose
NAb	Neutralizing Antibody
NIA	National Institute on Aging
OXM	Oxyntomodulin
PLT	Platelet
PT	Prothrombin time
RBC	Red Blood Cell
SAE	Severe Adverse Event

SOPs	Standard Operation Procedure
T2DM	Type 2 diabetes mellitus
TBIL	Total bilirubin
TC	Total cholesterol
TEAEs	Treatment Emergent Adverse Event
TG	Triglyceride
TIA	Transient ischemic attack
TSH	Thyroid Stimulating Hormone
UA	Uric Acid
Vd	Volume of distribution
WBC	White blood cell
WT	Wild type
Abbreviation	English full name
ADA	Anti-drug antibody
AE	Adverse Event
AESI	Adverse Event of Special Interest
ALB	Albumin
ALP	Alkaline phosphatase
ALT	Alanine transaminase
APTT	Activated partial thromboplastin time
AR	Accumulation Ratio
AST	Aspartate amino transferase
AUC	Area Under Curve
CK	Creatine Kinase
CL	Clearance

CONTENTS

SIGNATURE PAGE 2
VERSION HISTORY 3
ABBREVIATION 4
CONTENTS 6
1 INTRODUCTION 8
2 PROTOCOL DETAILS 8
2.1 STUDY OBJECTIVES 8
2.2 STUDY DESIGN 8
2.3 SAMPLE SIZE AND POWER 9
2.4 RANDOMIZATION AND MASKING 9
2.5 CHANGES TO PLANNED STATISTICAL ANALYSES IN THE PROTOCOL 10
2.5.1 Changes to Statistical Analysis in Protocol Amendments 10
2.5.2 Changes to Planned Analyses in the Statistical Analysis Plan 10
3 ENDPOINTS 12
3.1 SAFETY, TOLERABILITY AND MAXIMUM TOLERATED DOSE EVALUATION 12
3.2 IMMUNOGENICITY 12
3.3 PHARMACOKINETIC PARAMETERS 12
3.4 PHARMACODYNAMIC PARAMETERS 12
3.5 OTHER EFFICACY MEASURES 13
3.6 EXPLORATORY MEASURES 13
4 ANALYSIS DATASETS 13
4.1 ANALYSIS DATASETS 13
5 DATA PROCESSING 13
5.1 GENERAL SPECIFICATIONS 13
5.2 EFFICACY DATA HANDLING PRACTICES 13
5.3 MISSING VALUES AND OUTLIERS 13
5.4 TIME POINTS AND VISIT WINDOWS 15
6 STATISTICAL ANALYSIS METHODS 15
6.1 GENERAL PRINCIPLES 15
6.2 SUBJECT DISPOSITION 16
6.3 DEMOGRAPHIC AND OTHER BASELINE CHARACTERISTICS 17
6.3.1 Demographic and Baseline Characteristics 17
6.3.2 Medical History 18
6.3.3 Prior/Concomitant Therapy 19
6.4 EXTENT OF STUDY DRUG EXPOSURE AND COMPLIANCE 20
6.4.1 Extent of Study Drug Exposure 20
6.5 EFFICACY ANALYSIS 20
6.5.1 Analysis of HbA1c/fasting blood glucose/body weight/waist circumference/BMI 20
6.5.2 Analysis of fasting insulin/fasting c-peptide/lipid profile/blood pressure/HOMA-β/HOMA-IR/fasting endogenous OXM/fasting endogenous GLP-1 21
6.5.3 7-point self-measured glucose analysis 22
6.5.4 MTT Standard Meal Parameter Analysis 22
6.6 SAFETY ANALYSIS 22
6.6.1 Adverse events 22
6.6.2 Laboratory Inspection 24

6.6.3 Vital Signs *24*
6.6.4 Electrocardiogram *24*
6.6.5 Physical examination *24*
6.7 IMMUNOGENICITY ANALYSIS 24
6.8 PARMACOKINETIC ANALYSIS 24
6.9 ANALYSIS OF PHARMACODYNAMIC PARAMETERS 25
6.10 EXPLORATORY ANALYSES 25
6.11 INTERIM ANALYSES AND DATA MONITORING MEETINGS 25

7 REFERENCES 25

APPENDICES 26

APPENDIX 1 POTENTIALLY CLINICALLY SIGNIFICANT VITAL SIGNS AND ECG ABNORMALITIES 26

1 Introduction

This is the Statistical Analysis Plan (SAP) for the study CIBI362A101 "A Multiple Dose Tolerability and Pharmacodynamic Study of IBI362 in Chinese Patients with Type 2 Diabetes and Inadequate Glycemic Control" (Protocol No.: CIBI362A101, Version 1.1, Date 2021-01-15). This SAP summarizes the design and objectives of the study and is intended to provide a detailed definition of the endpoint measures in the protocol and a detailed description of the planned statistical analyses. The final SAP will be approved and signed prior to database lock.

2 Protocol Details

2.1 STUDY OBJECTIVES

- Primary objective
    - To investigate the safety and tolerability of multiple subcutaneous injections of IBI362 in patients with type 2 diabetes inadequately controlled by lifestyle intervention or metformin, and to determine the safe dose range for its clinical use.
- Secondary objectives
    - To investigate the pharmacokenetic/pharmacodynamic (PK/PD) parameters of multiple subcutaneous injection of IBI362 in patients with type 2 diabetes inadequately controlled by lifestyle intervention or metformin.

2.2 STUDY DESIGN

The study was planned to include 42 patients with type 2 diabetes who failed to achieve HbA1c control after at least 2 months of lifestyle intervention or stable dose of metformin (≥ 1000 mg/day or maximum tolerated dose). The study was divided into three cohorts, which were Cohort 1 (n = 14), Cohort 2 (n = 14), and Cohort 3 (n = 14), and patients in each cohort were randomly divided into IBI362 treatment group (n = 8), placebo group (n = 4), and dulaglutide 1.5 mg treatment group (n = 2) in an 8:4: 2 ratio. The active comparator drug, dulaglutide, was administered at 1.5 mg QW for 12 weeks in Cohorts 1, 2, and 3, and the dosing schedules for IBI362 and placebo are described below:

Cohort 1: The initial dose is 1.0 mg once weekly for 4 weeks. If well tolerated, the dose should be increased to 2.0 mg once weekly. After 4 weeks of treatment, the subject's tolerance should be evaluated again. If well tolerated, the dose should be increased to 3.0 mg once weekly for 4 weeks (escalation by 1 mg every 4 weeks to the target dose).

Cohort 2: The initial dose of the subject is 1.5 mg, once weekly for 4 weeks. If well tolerated, the dose will be increased to 3.0 mg, once weekly for 4 weeks. After that, the subject's tolerance will be evaluated again. If 3.0 mg cannot be tolerated according to the intolerance criteria, the subject need to initiate the next dose according to Cohort 2 (backup 1); if 3.0 mg is well tolerated, the dose will be increased to 4.5 mg, once weekly for 4 weeks. If 4.5 mg can not be tolerated according to the intolerance criteria, the subject need to initiate the next dose according to Cohort 2 (backup 2) (escalation by 1.5 mg every 4 weeks to the target dose).

Cohort 2 (backup 1): If 3.0 mg cannot be tolerated, the dose should be reduced to 2.25 mg after 1-week break and continued to be increased to 3.0 mg after 2-week treatment. If well tolerated, the dose should be increased to 3.0 mg and administered for 4 weeks.

Cohort 2 (backup 2): If 4.5 mg cannot be tolerated, the dose should be reduced to 3.75 mg after 1-week break and administered for 2 weeks till the end of the trial.

Cohort 3: Subjects should not start dosing until subjects in Cohort 2 complete the initial 4-week evaluaion at 1.5 mg dose. If subjects in Cohort 2 cannot tolerate 1.5 mg, 2.0 mg and higher doses will not be explored in Cohort 3. The initial dose of subjects in this cohort was 2.0 mg once weekly for 4 weeks. If well tolerated, the dose will be increased to 4.0 mg once weekly. After 4 weeks of administration, the subject's tolerance will be evaluated again. If well tolerated, the dose will be increased to 6.0 mg once weekly for 4 weeks (escalation by 2 mg every 4 weeks to the target dose).

The trial consisted of a 3-week screening period, a 12-week double-blind treatment period, and an 8-week safety follow-up period.

2.3 SAMPLE SIZE AND POWER

42 patients were enrolled in 3 dose cohorts. A total of 14 subjects were enrolled in each cohort at a ratio and randomized 8:4:2 to IBI362, placebo, and active control drug.

2.4 RANDOMIZATION AND MASKING

This is a randomized, double-blind trial of IBI362 versus placebo within each dose group. At each dose, subjects were randomized to receive IBI362, placebo, active control treatment in a ratio of 8:4:2. If a subject is assigned to receive IBI362 or placebo, the subject, the investigator, and all medical personnel involved in the treatment or clinical evaluation will be blinded to the treatment allocation from the time of randomization until the database is locked, as follows:

Different drug numbers were linked to random numbers in the central randomization system, and the blind base of drug numbers and random numbers remained masked to subjects and investigators throughout the trial. The randomization table was kept by a designated person.

The study drug and placebo will be packaged, labeled, administered at the same time, and will have the appearance, taste, and smell exactly the same to mask the real situation of the treatment drug.

Unblinding can only be performed in case of emergency and at the end of the study.

If a subject is randomized to receive active control, the subject will not be blinded.

2.5 CHANGES TO PLANNED STATISTICAL ANALYSES IN THE PROTOCOL

2.5.1 Changes to Statistical Analysis in Protocol Amendments

There were no statistical analysis changes in this protocol amendment.

2.5.2 Changes to Planned Analyses in the Statistical Analysis Plan

**Protocol**	**Statistical Analysis Plan**	
**Statistical description in the protocol**	**Statistical description in statistical analysis plan**	**Reason for change**
Efficacy Analysis Set: Subjects in the Safety Set who had at least 1 postbaseline assessment.	Efficacy Analysis Set: Subjects in the Safety Set who had at least 1 postbaseline assessment (glycated hemoglobin).<br>Anti-drug Antibody Analysis Set: includes subjects who have taken at least one dose of study treatment and have at least one post-dose valid ADA test result. Used for immunogenicity analysis.	Add the specific description of anti-drug antibody analysis set and refine the definition of efficacy analysis set.
To assess the pharmacodynamic parameters of IBI362 after multiple doses: changes in fasting blood glucose, glucagon, insulin, fasting C-peptide, HbA1c, endogenous oxyntomodulin (OXM) and GLP-1 before and after administration;	To assess the pharmacodynamic parameters of IBI362 after multiple doses: changes in fasting blood glucose, insulin, fasting C-peptide, HbA1c, endogenous oxyntomodulin (OXM) and GLP-1 before and after administration;	Analysis of glucagon was not done due to lack of data.
Changes in fasting blood glucose, insulin and glucagon at steady state in each dose phase during dose escalation;	Changes in fasting blood glucose and insulin at steady state in each dose phase during dose escalation;	Analysis of glucagon was not

Protocol	Statistical Analysis Plan	
**Statistical description in the protocol**	**Statistical description in statistical analysis plan**	**Reason for change**
		done due to lack of data.
All efficacy analyses were performed on the efficacy analysis set. Efficacy endpoints will be analyzed only descriptively. For continuous efficacy endpoint indicators, the corresponding means and 95% confidence intervals will be calculated for IBI362 subjects in each dose group, all placebo subjects and all active control subjects, respectively. A two-sample t-test will be used to calculate the p-value of IBI362 versus placebo for each dose group and provide the corresponding point estimate and 95% confidence interval of the difference. LOCF missing values will be used for imputation. For the categorical efficacy endpoints, the ratio within each dose group will be calculated and the 95% confidence interval within the group will be calculated using Clopper-Pearson; the difference between IBI362 placebo groups will be compared using the chi-square test and the corresponding 95% confidence interval will be calculated. Missing values for grouped endpoints will be imputed using the default non-response method.	Analysis of HbA1c was performed in the efficacy analysis set, analysis of pharmacodynamic parameters was performed in the PD analysis set, and other efficacy endpoint indicators were performed in the safety analysis set unless otherwise specified. For continuous efficacy endpoint indicators, mixed model repeated measures (MMRM)/ANCOVA model was used to estimate the point estimate and 90% confidence interval of each treatment group, and the point estimate and 90% confidence interval of the difference between each IBI362 treatment group/dulaglutide group and placebo. LOCF missing values will be used for imputation. Nominal p-values are provided for comparisons. No multiplicity adjustment will be made in this trial.	The primary endpoints of this trial are safety and tolerability. The number of subjects in each treatment group is small. 90% confidence interval will be provided. No multiplicity adjustment will be made. Only nominal p value will be provided.

3 Endpoints

3.1 SAFETY, TOLERABILITY AND MAXIMUM TOLERATED DOSE EVALUATION

- Safety and tolerability: During dose escalation, the incidence and severity of adverse events at each dose level, including chief complaint, physical examination, laboratory tests (hematology, blood chemistry, lipid panel, coagulation function, urinalysis, myocardial enzymes, serum amylase and/or lipase, thyroid function, calcitonin, etc.), vital signs (pulse, respiration, blood pressure, body temperature), 12-lead ECG and other examinations; treatment compliance; and record the name, clinical characteristics, severity, start and stop time, supportive care and outcomes of adverse events, correlation with the study drugs;

- Maximum tolerated dose: If the dose exploration is stopped when the criteria for stopping dose escalation are met at a certain dose level, the previous dose is the maximum tolerated dose. If the criteria are still not met when the maximum exploratory dose is reached, the maximum tolerated dose is greater than or equal to the maximum escalation dose.

3.2 IMMUNOGENICITY

- The incidence of anti-IBI362 antibody (ADA) and neutralizing antibody (NAb) in before and after dosing;

3.3 PHARMACOKINETIC PARAMETERS

- To evaluate the kinetic parameters of IBI362 in patients with type 2 diabetes, including but not limited to: time to peak plasma concentration (T_{max}), peak plasma concentration (C_{max}), area under the concentration-time curve (AUC), volume distribution (Vd), half-life ($T_{1/2}$), clearance (CL), accumulation ratio (AR), etc.;

3.4 PHARMACODYNAMIC PARAMETERS

- To assess the pharmacodynamic parameters of IBI362 after multiple doses: changes in fasting blood glucose, insulin, fasting C-peptide, HbA1c, endogenous OXM and GLP-1 before and after administration;

- Changes in fasting blood glucose and insulin at steady state in each dose phase during dose escalation;

- The change of each parameter of MTT test from baseline after multiple dose administration;

3.5 OTHER EFFICACY MEASURES

- Self-measeared blood glucose;

3.6 EXPLORATORY MEASURES

- To evaluate the effect of IBI362 on weight loss (changes in body weight, waist circumference and BMI);
- To assess the improvement of islet in IBI362: changes in islet β function (HOMA-Beta) and insulin resistance (HOMA-IR).

4 Analysis Datasets

4.1 ANALYSIS DATASETS

Safety Set: subjects who signed informed consent and received at least one dose of the study drug.

Efficacy Analysis Set: Subjects in the Safety Set who had at least 1 postbaseline assessment (glycated hemoglobin).

PK analysis set: including all the subjects who receive at least one dose of the investigational drug and have at least one effective concentration data of the detected components after medication.

PD analysis set: including all subjects who receive at least one dose of the study drug and have valid test results at baseline and after at least one dose.

Anti-drug Antibody Analysis Set: includes subjects who have taken at least one dose of study treatment and have at least one post-dose valid ADA test result. Used for immunogenicity analysis.

5 Data processing

5.1 GENERAL SPECIFICATIONS

5.2 EFFICACY DATA HANDLING PRACTICES

In this trial, efficacy data will be analyzed as it is and, if not otherwise specified, missing values will not be imputed.

5.3 MISSING VALUES AND OUTLIERS

- **Handling of Related Variables Calculated if Baseline Data Date is Missing**

  Without affecting the logic, impute as follows:

  - If day/month/year are all missing, no imputation will be done;
  - If the day and month are missing, impute it to July 1 unless contradicts with other dates;
  - If the date is missing, impute day to 15 unless contradicts with other dates.

- **Handling of completely/partially missing values for prior/concomitant/non-drug therapy dates**

If the start/end dates or time of prior/concomitant medication/non-drug therapy are completely or partially missing, no imputation is needed. Completely or partially missing situations where it is impossible to determine prior or concomitant will be treated as concomitant.

In the analysis of duration of specified concomitant medication, if the end time is missing, impute it to the data cutoff date, death date or withdrawal date, whichever is earlier.

- **Handling of Completely/Partially Missing Adverse Event Dates**

If the AE start date is completely or partially missing, and it is not clear whether the data already collected in the eCRF is before or after the first dose of study treatment, the AE is considered to be a treatment-emergent AE. If it is necessary to calculate the duration of adverse event or mark the cycle number of adverse events, the completely/partially missing start/end dates will be imputed as follows without affecting the logic:

  - If day/month/year of the start date are all missing, impute it to the date of first study treatment;
  - If month and day of the start date are missing and it is in the same year as the first study treatment date, unless contradict with other collected data (such as end date, etc.), impute it to the first study treatment date, otherwise July 1st,
  - If only the day is missing and there is no contradiction with other collected data (such as end date, etc.), and it is in the same year and month as the first study treatment date, impute it to the first study treatment date, otherwise impute it to day 15,
  - If the imputed start date is after the end date, it should be set to the end date,
  - If day/month/year of the end date are all missing, it shall be considered as a continued adverse event;
  - If the month and day of the end date are missing (or only day is missing), then impute it to the last day of the known year (or month and year).

- **Treatment-emergent adverse event designation when start date/time of first study treatment is missing**

Adverse events that occurred on or after randomization will be considered treatment-emergent.

The imputation rule of completely/partially missing dates will only be used to flag or derive variables for the analysis, and the original dates as recorded on the eCRF will be presented in the listings.

- **Missing Adverse event relationship to study drug**

An adverse event will be considered related if its relationship to study drug is missing.

- **Missing Adverse Event Severity**

In the analysis by severity grade, adverse events with missing severity grade will be counted in the number/frequency of severe cases.

Subjects with missing baseline values will be classified as "missing" in the cross-tabulation of baseline vs. worst post baseline results.

- **Handling of outliers**

Outlier will be analyzed as it is.

- **Other Data Handling Considerations**

If the reported value of a laboratory parameter cannot be used in the statistical summary tables (e.g., the value of a numeric parameter is reported as a string), the coded value will be determined appropriately and used in the statistical analysis. Values higher than the upper limit of the test will be added 0.0001, e.g. "> 100" will be treated as "100.0001"; values lower than the lower limit of the test will be subtracted by 0.0001, e.g. "< 100" will be treated as "99.9999". The actual values reported in the database will still be presented in the listings.

5.4 TIME POINTS AND VISIT WINDOWS

For the statistical analysis summarized by visit, only the scheduled visit was used.

- Use of data from unscheduled visits:

Unscheduled visit data will be used for baseline value. The unscheduled visit data of laboratory tests, vital signs, ECG and other safety indicators are not used for by-visit summary analysis, but should be included for last visit and worst value record (PCSA abnormal value).

6 Statistical Analysis Methods

6.1 GENERAL PRINCIPLES

All statistical analyses will be performed using SAS 9.4 (or later version). This trial is mainly based on descriptive statistics, and in principle, no comparison will be made.

All data collected in this study will be presented in data listings and summarized in tables by treatment group, unless otherwise specified. Subjects receiving placebo in different cohorts will be combined and analyzed in summary tables.

Categorical data were presented as frequency (percentage). Unless otherwise specified, the number of subjects in each treatment group in the corresponding study population will be the denominator for the percentages. Percentages will be displayed to 1 decimal place.

Continuous data will be shown as mean ±standard deviation or median (minimum, maximum). The mean and median will be displayed to 1 more decimal place than the original data, the standard deviation will be displayed to 2 more decimal places than the original data, and the maximum and minimum values will be displayed to the same decimal place as the original data. For PK parameters, geometric means and coefficients of variation will be displaued to the first decimal place, unless otherwise specified.

Baseline value: defined as the last available measurement prior to revieving the first dose of study treatment.

The following notation will be used in the tables of statistical analysis:

**Treatment Group in Protocol**	**Treatment Groups in Statistical Analysis**
**Cohort 1, treated with IBI362**	IBI362 – Cohort 1
**Cohort 2, treated with IBI362**	IBI362 – Cohort 2
**Cohort 3, treated with IBI362**	IBI362 – Cohort 3
**Cohort 1, Cohort 2, Cohort 3, Placebo Treated**	Placebo
**Cohort 1, Cohort 2, Cohort 3, treated with dulaglutide 1.5 mg**	Dulaglutide 1.5 mg

6.2 SUBJECT DISPOSITION

- **Subject Disposition**

  Summary of subject disposition will include the following information:

  - Number of subject screened and reasons for screen failure
  - The number and percentage of subjects who were randomized, treated, prematurely discontinued study drug, and prematurely discontinued from the study. Subjects who withdrew early from the study will be listed.

    Premature discontinuation of study drug is defined as failure to receive the last scheduled dose of study drug.

- The number and percentage of subjects in each analysis population (ie, safety analysis set, efficacy analysis set, PK analysis set, PD analysis set, and antidrug antibody analysis set) will be summarized by treatment group. The inclusion or exclusion of each subject from the analysis population and the reason for exclusion will be presented in the listings.

- **Protocol Deviations**

In the safety analysis set, the number and percentage of subjects with major protocol deviations during the study will be summarized by treatment group and type of protocol deviation, and subjects with protocol deviations will be listed along with their detail description.

6.3 DEMOGRAPHIC AND OTHER BASELINE CHARACTERISTICS

6.3.1 Demographic and Baseline Characteristics

Descriptive statistics for demographic data and baseline characteristics will be summarized by treatment group in the safety analysis set.

The following continuous variables will be summarized using number of cases, mean, standard deviation, median, Q1 and Q3, minimum and maximum:

- Age (years) = (date of informed consent – date of birth + 1)/365.25, rounded off at the lowest integer;
- Height (cm)
- Weight (kg)
- Body mass index (kg/m^2)
- Waist circumference (cm)
- Fasting plasma glucose (mmol/L)
- Total cholesterol (mmol/L)
- Triglycerides (mmol/L)
- Low-density lipoprotein (mmol/L)
- High density lipoprotein (mmol/L)
- Glycosylated hemoglobin (%)
- Alanine aminotransferase (ALT) (IU/L)
- Aspartate aminotransferase (AST) (IU/L)
- Duration of T2DM history (year) = (date of informed consent – date of T2DM diagnosis + 1)/365.25

The number and percentage of subjects will be summarized for the following categorical variables:

- Gender: Male, Female
- Ethnicity: Asian, Other
- Ethnic group: Han, others
- Main method of glycemic control in the previous two months: lifestyle intervention, stable dose of metformin therapy

Other efficacy variables with baseline measurements (insulin, fasting C-peptide, OXM, GLP-1, etc.) and safety variables (laboratory tests, vital signs, ECG, etc.) will be analyzed together with post-baseline measurements in the relevant sections.

Demographic data and baseline characteristics of subjects will be listed.

6.3.2 Medical History

- **Disease History**

Descriptive statistics for history of diagnosis of the study disease were summarized by treatment group in the safety analysis set.

The following continuous variables were summarized by number of cases, mean, standard deviation, median, Q1, Q3, minimum, and maximum:

- Time on T2DM with medical history (years) = (Date of informed consent – Date of diagnosis of T2DM + 1)/365.25

Incomplete dates of diagnosis of type 2 diabetes were imputed according to rules in Section 5.3.

The number and percentage of subjects will be summarized for the following categorical variables:

- Glycemic control methods in the previous two months

A summary of study disease history was presented in the subject's baseline characteristics table.

Subjects' medical history of type 2 diabetes was tabulated.

- **History of non-study disease**

The MedDRA dictionary (version 24.0 or later) will be used to code the preferred term (PT) and system organ class (SOC), respectively, for medical history including surgical history. In the safety analysis set, the number and percentage of subjects with a medical history will be summarized by treatment group, SOC and PT. If a subject had multiple medical or allergy histories in the same SOC or PT category, the subject will be counted only once in that SOC and PT category.

The history of the disease under study and the history of non-study diseases will be listed.

6.3.3 Prior/Concomitant Therapy

- **Prior/concomitant medications**

The WHO-DD Dictionary (Version March 2021 or newer) will be used to code the Anatomical Therapeutic Chemical (ATC) Level 1-4 terms for prior/concomitant medications and the medication name (PN), respectively. In the safety analysis set, the number and percentage of subjects with prior/concomitant medications will be summarized by ATC level 2, ATC level 4, and PN as well as by treatment group. If a subject had multiple prior/concomitant medications in the same ATC Level 2, ATC Level 4, or PN category, the subject will be counted only once in that ATC Level 2, ATC Level 4, or PN category.

Prior medications in defined as medications that have started before receiving the first dose of study treatment and that have stopped before receiving the first dose of study treatment.

Concomitant medication is defined as medications that were taken from the day of the first dose of study treatment through 56 days after the last dose of study treatment, including medications that started before the first study treatment and were ongoing after the first study treatment, and medications that started from the day of the first study treatment through 56 days after the last dose.

- **Prior/Concomitant Non-Drug Therapies**

The MedDRA dictionary (version 24.0 or later) will be used to code the preferred term (PT) and system organ class (SOC) for prior and concomitant non-drug therapies, respectively. In the safety analysis set, the number and percentage of subjects with prior and concomitant non-drug therapies will be summarized by treatment group, SOC and PT. If a subject had multiple prior and concomitant non-drug therapies in the same SOC or PT category, the subject will be counted only once in that SOC and PT category.

Prior non-drug therapy is defined as receiving a non-drug therapy that had started before the first dose of study treatment and had stopped before the first dose of study treatment.

Concomitant nondrug therapy is defined as nondrug therapy that started or was continuing from the day of first dose of study treatment through 56 days after the last dose, including those that started before the first dose of study treatment and were continuing after the first dose of study treatment, and those that started from the day of first dose of study treatment through 56 days after the last dose.

Prior/concomitant medications and non-drug therapies will be tabulated.

6.4 EXTENT OF STUDY DRUG EXPOSURE AND COMPLIANCE

The extent of exposure to study drug was assessed and summarized in the safety analysis set.

6.4.1 Extent of Study Drug Exposure

Assessments of the extent of exposure to study drug include cumulative exposure to study drug, relative dose intensity and weeks of exposure.

Cumulative exposure, relative dose intensity and weeks of exposure to study drug will be summarized by treatment group with the following statistics: number of subjects, mean, standard deviation, median, Q1, Q3, minimum, and maximum.

Relative dose intensity (%) = (cumulative drug exposure/ total planned dose) × 100%, the total planned dose is the sum of the planned doses administered throughout the treatment period.

Exposure weeks are calculated as (date of last dose – date of first dose + 7 days)/7 days.

Exposure of study treatment will be listed.

6.5 EFFICACY ANALYSIS

Unless otherwise specified, the analysis of HbA1c will be performed in the efficacy analysis set, the efficacy analysis of PD parameters will be performed in the PD analysis set, and the analysis of other efficacy indicators will be performed in the safety analysis set.

6.5.1 Analysis of HbA1c/fasting blood glucose/body weight/waist circumference/BMI

Baseline and different post-baseline visits were descriptively analyzed by treatment group (Week 5, Week 9 and Week 13) for the following efficacy measures: HbA1c, fasting blood glucose, body weight, waist circumference, and BMI and their change values from baseline, and the mean values of HbA1c and fasting blood glucose and their change from baseline over time will be plotted. Percent change from baseline over time will be plotted for body weight. Change from baseline over time will be plotted for waist circumference and BMI.

For HbA1c and fasting blood glucose, a mixed model repeated measures (MMRM) model with baseline variables will be used to calculate the point estimate and 90% confidence interval of the change from baseline after treatment, and the point estimate and 90% confidence interval of the difference between each IBI362 cohort and placebo. Nominal p-values for comparisons are provided. The SAS program for the MMRM model references the following:

```
Proc mixed data = indata;
```

```
Class treatment (ref ="placebo") usubjid time;
Model chg = base treatment time treatmenttime/solution;
Repeated time/type = un sub = usubjid (treatment) rcorr;
Lsmeans treamenttime/diff = all alpha = 0.1;
Ods output lsmeans = datm diffs = datd;
Run;
```

Where treatment is the treatment group, time is each visit, chg is the change from baseline of analysis indicators, and base is the corresponding baseline value. The unstructured correlation was used to estimate the correlation coefficient matrix of change from baseline at each visit for each subject in the MMRM model. The likelihood of the model was constructed using the REML method (SAS proc mixed default likelihood method).

If necessary, MMRM model will be used to estimate the point estimate and 90% confidence interval of each treatment group, and the point estimate and 90% confidence interval of the difference between each IBI362 treatment group/polaglutide group and placebo for other repeated measures efficacy endpoints. Nominal p-values are provided for comparisons.

Results were tabulated.

6.5.2 Analysis of fasting insulin/fasting c-peptide/lipid profile/blood pressure/HOMA-β/HOMA-IR/fasting endogenous OXM/fasting endogenous GLP-1

The following efficacy indicators after baseline and at D85 visit were descriptively analyzed by treatment group: fasting insulin, fasting c-peptide, blood lipids (triglycerides, total cholesterol, low-density cholesterol and high-density cholesterol), blood pressure (systolic blood pressure, diastolic blood pressure), HOMA-β, HOMA-IR, fasting endogenous OXM and fasting endogenous GLP-1 and their changes from baseline.

If necessary, ANCOVA model will be used to calculate the point estimate and 90% confidence interval of each treatment group and the point estimate and 90% confidence interval of the difference between each IBI362 treatment group/dulaglutide group and placebo. Missing values will be imputed using LOCF in the ANCOVA model and the nominal p-values for the comparisons were provided.

The SAS program for the ANCOVA model can be found at:

```
Proc mixed data = indata method = type3;
Class treatment (ref ="placebo");
```

```
Model chg = base treatment;
Lsmeans treatment/diff = control ("placebo") alpha = 0.1;
Ods output lsmeans = datmean diffs = datdiff;
Run;
```

Where treatment is the treatment group, chg is the change from baseline value of the analysis indicator, and base is the corresponding baseline value.

Lipid panel results were tabulated. Blood pressure test results will be presented in the listing of vital sign test results. Listings of HOMA-β and HOMA-IR results were presented in the listing of pharmacodynamic test results.

6.5.3 7-point self-measured glucose analysis

The blood glucose results at each test point of 7-point fingertip glucose at baseline and D85 visit will be summarized by treatment group, and the change curve of 7-point fingertip glucose over the measurement time point will be plotted.

The 7-point fingerstick glucose test results were tabulated.

6.5.4 MTT Standard Meal Parameter Analysis

The parameters for each test point of the MTT standardized meal test were summarized descriptively by treatment group at baseline and D80 visit: insulin, C-peptide, plasma glucose, endogenous OXM, and endogenous GLP-1. MTT standard meal baseline and D80 visit were plotted over time for each parameter. If necessary, calculate the area under the curve (AUC0-4h) between two visits for each parameter and calculate the difference, providing the confidence interval.

MTT standard meal test results were tabulated.

6.6 SAFETY ANALYSIS

Safety analysis will be performed in the safety analysis set and grouped by actual treatment received.

6.6.1 Adverse events

Classified by date of event:

- Pre-treatment Adverse Events: Adverse events that occurred from signing of informed consent until the first dose of study treatment.
- Treatment-emergent adverse events (TEAEs): Adverse events that occurred or became serious during the first dose of study treatment through 56 days after last dose.
- Post-treatment adverse events: Adverse events that occurred or became serious after 56 days following the last dose until the end of the trial.

If the AE start date is completely/partially missing, impute the date according to Section 5.3 before assigning pre-treatment/on-treatment/post-treatment adverse events.

The incidence (frequency) of all AEs, TEAEs, severe TEAEs, TEAEs leading to permanent treatment discontinuation, drug-related TEAEs (TRAEs), severe TRAEs, TRAEs leading to permanent treatment discontinuation, AESIs, severe AESIs, AESIs leading to permanent treatment discontinuation, SAEs, SAEs related to study drug, and SAEs leading to permanent treatment discontinuation will be summarized by treatment group.

The following categories of adverse events will be summarized by SOC, PT, and SOC, PT, and severity from MedDRA coding:

- TEAEs
- TEAE leading to permanent discontinuation
- TRAE
- TRAEs leading to permanent discontinuation
- AESI
- AESIs leading to permanent discontinuation
- For gastrointestinal reactions, the number of events with gastrointestinal reactions was also provided. Gastrointestinal reactions including adverse reactions with preferred terms of nausea, vomiting, diarrhea, dyspepsia, abdominal distension, abdominal discomfort, and gastroesophageal distress

TEAEs will also be summarized by PT in MedDRA and the number of events of TEAEs will be calculated.

Treatment-emergent SAEs, treatment-emergent SAEs related to study drug, and treatment-emergent SAEs leading to permanent discontinuation will be summarized by SOC and PT.

Hypoglycemic events will be summarized by treatment group.

The above summary tables are sorted according to the incidence of AEs in all subjects who received IBI362.

Subjects who discontinued treatment due to AEs, subjects who experienced SAEs, and subjects who died will be listed (at least include AE start date, end date, severity, relationship with drug, action taken, and outcome). All AEs will be listed.

6.6.2 Laboratory Inspection

For each laboratory tests, a cross-tabulation of baseline and the worst post-baseline result will be summarise in the following categories: normal or abnormal and presence or absence of clinical significance.

Laboratory test results will be listed, and pregnancy test results and/or virological tests during the trial will be listed separately.

6.6.3 Vital Signs

Vital signs (including body temperature, pulse and respiratory rate) and their changes from baseline will be listed and summarized by visit. Worst results for potentially clinically significant postbaseline vital signs will be summarized according to the PCSA criteria in Appendix 1.

Vital sign results will be listed.

6.6.4 Electrocardiogram

ECG parameters to be analyzed included heart rate, QRS duration, QT interval, QTCF, and PR interval.

The results of ECG examination and changes from baseline will be summarized at each evaluation time point. ECG parameters were plotted over time. Change from baseline in heart rate was plotted over time. Worst results of potentially clinically significant post-baseline ECGs were summarized according to PCSA criteria in Appendix 1.

ECG findings will be listed.

6.6.5 Physical examination

Provide a listing of all physical examinations for each subject.

6.7 IMMUNOGENICITY ANALYSIS

The positive rates of neutralizing GLP-1 and GCGR against anti-drug antibody (ADA) and neutralizing antibody (NAb) in subjects receiving IBI362 will be summarized by treatment group.

Antibody levels and immunogenicity result will be listed for ADA positive subjects.

6.8 PARMACOKINETIC ANALYSIS

For subjects who received IBI302, PK parameters will be summarized descriptively by treatment group. PK parameters include: T_{max}, C_{max}, area under the concentration-time curve (AUC), volume of distribution (Vd), half-life ($T_{1/2}$), clearance (CL), and accumulation coefficient (AR). Detailed PK analyses are provided in a separate PK analysis report.

Plasma concentrations of IBI362 will be listed for subjects using IBI362.

6.9 ANALYSIS OF PHARMACODYNAMIC PARAMETERS

Pharmacodynamic measures include HbA1c, fasting c-peptide, fasting plasma glucose, insulin, OXM, and GLP-1. Relevant analyses are described in the efficacy analysis sections 6.5.1 and 6.5.2.

Pharmacodynamic test results will be listed.

6.10 EXPLORATORY ANALYSES

Exploratory measures included fasting weight, waist circumference, BMI, HOMA-β, and HOMA-IR. The relevant analyses are described in 6.5.1 and 6.5.2.

6.11 INTERIM ANALYSES AND DATA MONITORING MEETINGS

There is no planned interim analysis and data monitoring meetings for this trial.

7 References

1 China State Food and Drug Administration. Good Clinical Practice (GCP). 2013.

2 China State Food and Drug Administration. Guidelines for the Structure and Content of Clinical Study Reports of Chemical Drugs. March 2005.

3 China State Food and Drug Administration. Guidelines for Biostatistics of Drug Clinical Trials (Draft for Comments). June 3, 2016.

4 ICH. ICH E9 Guideline: Statistical Principles for Clinical Trials, 1998. Available at https://www.ich.org/fileadmin/Public_Web_Site/ICH_Products/Guidelines/Efficacy/E9/Step4/E9_Guideline.pdf

5 ICH. ICH E3 Guideline: Structure and Content of Clinical Study Reports Questions & Answers, 2012. Available at http://www.ich.org/fileadmin/Public_Web_Site/ICH_Products/Guidelines/Efficacy/E3/E3_QAs_R1_Step4.pdf

APPENDICES

APPENDIX 1 POTENTIALLY CLINICALLY SIGNIFICANT VITAL SIGNS AND ECG ABNORMALITIES

**Vital Signs**	**Standard**	**Remarks**
Pulse	≤ 50 bpm and decrease from baseline ≥ 20 bpm<br>≥ 120 bpm and increase from baseline ≥ 20 bpm	All positions except standing
Systolic blood pressure	≤ 95 mmHg and decrease from baseline ≥ 20 mmHg<br>≥ 160 mmHg and increase from baseline ≥ 20 mmHg	All positions except standing
Diastolic	≤ 45 mmHg and decrease from baseline ≥ 10 mmHg<br>≥ 110 mmHg and ≥ 10 mmHg increase from baseline	All positions except standing
Weight	≥ 5% increase from baseline<br>≥ 5% reduction from baseline	
**Electrocardiogram**	**Standard**	Remarks
HR	≤ 40 bpm and decrease from baseline ≥ 20 bpm<br>≥ 100 bpm and increase from baseline ≥ 20 bpm	Categories are summarized as cumulative
PR	≥ 220 ms	Categories are summarized as cumulative
QRS	≥ 120 ms	Categories are summarized as cumulative
QT	Absolute (ms)<br><br>> 450 ms and ≤ 480 ms<br>> 480 ms and ≤ 500 ms<br>> 500 ms<br><br>Increase from Baseline<br>[30-60] ms<br>> 60 ms	Categorical summaries of absolute values are cumulative

**Vital Signs**	**Standard**	**Remarks**
QTcF	Absolute (ms)<br><br>> 450 ms and ≤ 480 ms<br>> 480 ms and ≤ 500 ms<br>> 500 ms<br><br>Increase from Baseline<br>[30-60] ms<br>> 60 ms	Categorical summaries of absolute values are cumulative<br><br>QTc > 480 ms and ΔQTc > 60 ms are the 2 PCSA categories to be identified in individual subjects/patients listings.
Source: ICH E14 guidance (2005) and E14 QampA (2020), and Cardiac Safety Research Consortium White Paper on PR and QRS (Nada et al. Am Heart J. 2013; 165 (4): 489-500)		
