# Supplementary material for: A phase 1b randomised controlled trial of a glucagon-like peptide-1 and glucagon receptor dual agonist IBI362 (LY3305677) in Chinese patients with type 2 diabetes
Source: Nat Commun. 2022 Jun 24;13:3613. doi: 10.1038/s41467-022-31328-x (PMC9232612; doi:10.1038/s41467-022-31328-x)
Supplement: Supplementary file 3 — Reporting Summary [file 41467_2022_31328_MOESM3_ESM.pdf]

nature research

Corresponding author(s): Wenying Yang and Lei Qian

Last updated by author(s): May 28, 2022

Reporting Summary

Nature Research wishes to improve the reproducibility of the work that we publish. This form provides structure for consistency and transparency in reporting. For further information on Nature Research policies, see our Editorial Policies and the Editorial Policy Checklist.

Statistics

For all statistical analyses, confirm that the following items are present in the figure legend, table legend, main text, or Methods section.

n/a	Confirmed	
☐	☒	The exact sample size (*n*) for each experimental group/condition, given as a discrete number and unit of measurement
☐	☒	A statement on whether measurements were taken from distinct samples or whether the same sample was measured repeatedly
☐	☒	The statistical test(s) used AND whether they are one- or two-sided<br>*Only common tests should be described solely by name; describe more complex techniques in the Methods section.*
☐	☒	A description of all covariates tested
☐	☒	A description of any assumptions or corrections, such as tests of normality and adjustment for multiple comparisons
☐	☒	A full description of the statistical parameters including central tendency (e.g. means) or other basic estimates (e.g. regression coefficient) AND variation (e.g. standard deviation) or associated estimates of uncertainty (e.g. confidence intervals)
☐	☒	For null hypothesis testing, the test statistic (e.g. *F*, *t*, *r*) with confidence intervals, effect sizes, degrees of freedom and *P* value noted<br>*Give P values as exact values whenever suitable.*
☒	☐	For Bayesian analysis, information on the choice of priors and Markov chain Monte Carlo settings
☒	☐	For hierarchical and complex designs, identification of the appropriate level for tests and full reporting of outcomes
☒	☐	Estimates of effect sizes (e.g. Cohen's *d*, Pearson's *r*), indicating how they were calculated

Our web collection on statistics for biologists contains articles on many of the points above.

Software and code

Policy information about availability of computer code

Data collection	HbA1c, blood glucose, insulin, C peptide, GLP-1 and OXM were analyzed in a central laboratory (KingMed Diagnostics, Guangzhou, China). HbA1c was measured using a Bio-rad D-100 Hemoglobin Testing System (Bio-rad). Blood glucose was measured using a Roche Cobas c702 analyzer (Roche Diagnostics). Insulin and C peptide were measured using a Roche Cobas c602 analyzer (Roche Diagnostics). GLP-1 and OXM were measured using a Tecan Infinite M Plex microplate reader (TECAN).
Data analysis	Standard non-compartmental pharmacokinetics methods were used to analyze IBI362 plasma concentration data using Pkanalix 2020R1 (Lixoft, Antony, France).<br>All statistical analyses were done using SAS version 9.4.

For manuscripts utilizing custom algorithms or software that are central to the research but not yet described in published literature, software must be made available to editors and reviewers. We strongly encourage code deposition in a community repository (e.g. GitHub). See the Nature Research guidelines for submitting code & software for further information.

Data

Policy information about availability of data

All manuscripts must include a data availability statement. This statement should provide the following information, where applicable:

- Accession codes, unique identifiers, or web links for publicly available datasets
- A list of figures that have associated raw data
- A description of any restrictions on data availability

Individual de-identified patient data underlying the results reported in this article will be made available upon reasonable written request to the corresponding authors (WY or LQ) from researchers whose proposed aims has been approved, beginning 9 months and ending 36 months following article publication. Clinical

Study Protocol and Statistical Analysis Plan are provided as Supplementary Note 1 and Supplementary Note 2, respectively. Source data are provided with this paper.

Field-specific reporting

Please select the one below that is the best fit for your research. If you are not sure, read the appropriate sections before making your selection.

☒ Life sciences ☐ Behavioural & social sciences ☐ Ecological, evolutionary & environmental sciences

For a reference copy of the document with all sections, see nature.com/documents/nr-reporting-summary-flat.pdf

Life sciences study design

All studies must disclose on these points even when the disclosure is negative.

Sample size	The sample size was determined based on sample sizes commonly used in previous early phase studies of medications in the same drug class (Ambery, P. D. et. al. Lancet 2018, Tillner J. et al. Diabetes Obes Metab 2018). Eight patients received IBI362 in each cohort to provide preliminary safety, tolerability and pharmacokinetics data of IBI362, while a total of six patients received open-label dulaglutide to provide a reference for safety and efficacy.
Data exclusions	No data was excluded from the analysis.
Replication	This study has three cohorts, with eight patients receiving ascending doses of IBI362 (n = 8 for each dose regimen), four receiving placebo (n = 12 in total) and two receiving open-label dulaglutide (n = 6 in total) in each cohort. This allowed replication and reproducibility of findings across different treatment groups.
Randomization	An interactive web-response system generated identification numbers that were used to randomly assign eligible patients 8:4:2 to receive IBI362, placebo or open-label dulaglutide in each cohort. Randomization schedule was generated by an in-house statistician who was not involved in the clinical operations of the study.
Blinding	IBI362 and placebo were identically labelled and indistinguishable in appearance. As such, the patients, investigators, study site personnel involved in treating and assessing patients and sponsor personnel in each cohort were masked to IBI362 and placebo allocation.

Reporting for specific materials, systems and methods

We require information from authors about some types of materials, experimental systems and methods used in many studies. Here, indicate whether each material, system or method listed is relevant to your study. If you are not sure if a list item applies to your research, read the appropriate section before selecting a response.

Materials & experimental systems

n/a	Involved in the study	
☒	☐	Antibodies
☒	☐	Eukaryotic cell lines
☒	☐	Palaeontology and archaeology
☒	☐	Animals and other organisms
☐	☒	Human research participants
☐	☒	Clinical data
☒	☐	Dual use research of concern

Methods

n/a	Involved in the study	
☒	☐	ChIP-seq
☒	☐	Flow cytometry
☒	☐	MRI-based neuroimaging

Human research participants

Policy information about studies involving human research participants

Population characteristics	This study enrolled Chinese patients diagnosed with type 2 diabetes that inadequately controlled by lifestyle intervention alone or with stable metformin (≥ 1000 mg/day or maximum tolerated dose) within 2 months prior to screening. The ages of enrolled patients ranged from 34 to 66 years old. Twenty-six patients (61.9%) were male. Baseline HbA1c levels of enrolled patients ranged from 6.9% to 10.5%. Baseline fasting plasma glucose levels ranged from 8.11 mmol/L to 16.15 mmol/L. Baseline body mass index of enrolled patients ranged from 21.8 kg/m2 to 32.7 kg/m2.<br>The analyses for change from baseline in HbA1c, FPG, post-MTT glucose AUC0–4 h and body weight were performed using MMRM, with corresponding baseline value, treatment, visit and treatment-by-visit as covariates. In the overall population (n = 42), mean HbA1c level was 8.54% (SD 0.93); mean FPG level was 11.2 mmol/L (SD 2.2); mean post-MTT glucose AUC0–4 h was 3816.3 mmol*hr/L (SD 560.6); mean body weight was 69.5 kg (SD 11.1).
Recruitment	Investigators from nine clinical study centers in China screened and enrolled patients into this study. Patients must meet the all the inclusion criteria and should not meet any one of the exclusion criteria as defined in the clinical study protocol. All patients provided written informed consent before study entry. This was a randomized, double-blind study where the

	patients, investigators, study site personnel involved in treating and assessing patients and sponsor personnel in each cohort were masked to IBI362 and placebo allocation. Possibility for potential self-selection bias or other biases were minimized with the strict adherence to the inclusion and exclusion criteria, and randomization and masking procedures as defined in the protocol.
Ethics oversight	The clinical study protocol, the protocol amendment and informed consent forms were approved by the ethics committee at each participating study center: the ethics committee of Henan University of Science and Technology First Affiliated Hospital (Jinghua Division); the ethics committee of Henan University of Science and Technology First Affiliated Hospital (Kaiyuan Division); the ethics committee of Jinan Central Hospital; the ethics committee of Pingxiang People's Hospital; the ethics committee of China-Japan Friendship Hospital; the ethics committee of Bengbu Medical College First Affiliated Hospital; the ethics committee of Guizhou Medical University Affiliated Hospital; the ethics committee of Shanxi Medical University First Affiliated Hospital; the ethics committee of Tonghua Central Hospital.

Note that full information on the approval of the study protocol must also be provided in the manuscript.

Clinical data

Policy information about clinical studies

All manuscripts should comply with the ICMJE guidelines for publication of clinical research and a completed CONSORT checklist must be included with all submissions.

Clinical trial registration	This study is registered with ClinicalTrials.gov, number NCT04466904.
Study protocol	The full clinical study protocol was supplemented in the Supplemental Information file.
Data collection	Between September 12th, 2020 and May 28th, 2021, patients were enrolled at and data were collected from the following study centers: China-Japan Friendship Hospital, Jinan Central Hospital, Pingxiang People's Hospital, Henan University of Science and Technology First Affiliated Hospital (Jinghua Division), Henan University of Science and Technology First Affiliated Hospital (Kaiyuan Division), Bengbu Medical Collelge First Affiliated Hospital, Guizhou Medical University Affiliated Hospital, Tongnghua Central Hospital, Shanxi Medical University First Affiliated Hospital.
Outcomes	Primary endpoints of the study were safety and tolerability of IBI362. Safety of IBI362 were assessed by incidence and severity of adverse events, physical examinations, laboratory tests (including lipase, amylase and calcitonin level measures), vital signs and 12-lead electrocardiogram.<br>Secondary endpoints included pharmacokinetics, assessed by maximum observed plasma concentration (Cmax), time at which Cmax was observed (Tmax), terminal elimination half-life (t½), and AUC from time zero to 168 h after the first dose (AUC0–168 h) and immunogenicity, assessed by titers of anti-drug antibodies (ADAs) and neutralizing antibodies. Secondary efficacy endpoints included changes from baseline to week 12 in HbA1c, FPG, fasting insulin levels, as well as in post-MTT glucose, insulin, C peptide, GLP-1 and OXM levels.
